# Functionalization of Electron-Rich Secondary Benzyl Alcohols in HFIP: From Thioethers to Trisubstituted Methanes

**DOI:** 10.1021/acs.joc.5c00900

**Published:** 2025-06-28

**Authors:** Martyna Markwitz, Klaudiusz Labrzycki, Kacper Łyczek, Krzysztof Kuciński

**Affiliations:** Faculty of Chemistry, 49562 Adam Mickiewicz University, Poznań, Uniwersytetu Poznańskiego St. 8, Poznań 61-614, Poland

## Abstract

To date, C–S bond formation via dehydration of electron-rich secondary benzylic alcohols has been limited to catalytic approaches. In this work, we developed an efficient method for synthesizing various thioethers by employing hexafluoroisopropanol (HFIP) as a solvent. This approach also enables the functionalization of secondary benzyl alcohols with other important organic molecules, such as allylsilanes, activated arenes, and indoles, leading to various trisubstituted methanes, including 14 novel products in total.

## Graphical abstract

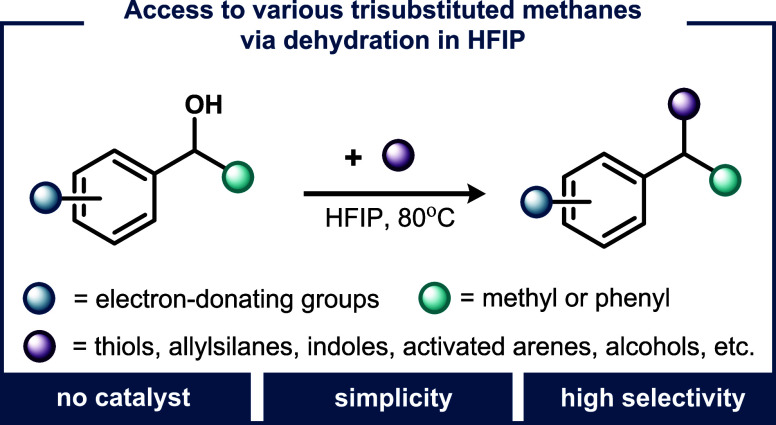

Dehydration reactions typically require additional catalysts, primarily Brønsted or Lewis acids, due to the poor leaving group ability of the hydroxyl group.^1^ An additional, and often essential, factor is elevated temperature. In recent years, a large number of scientific studies have explored the impact of hexafluoroisopropanol (HFIP) on the course of various reactions.^2–11^ HFIP is significantly more acidic than typical alcohols (p*K*_a_ ∼ 9.3 vs ethanol p*K*_a_ ∼ 16) and also has a strong ability to form hydrogen bonds and stabilize charged transition states.^12,13^ Considering all these factors, we hypothesized that dehydration reactions should readily occur using HFIP^14^ as the sole solvent, without the need for additional strong acids. Dehydration represents an efficient and widely utilized approach for the synthesis of thioethers,^15^ which are of considerable interest due to their significance as key biochemicals and building blocks.^16–20^ Previous methods for their synthesis mainly relied on the use of transition metal compounds,^21–25^ and metal triflates.^26,27^ Recent studies in this area, however, have focused on metal-free solutions, where strong triflic acid,^28,29^ ionic liquids^30^ or triaryl-carbenium ion pairs^31^ were used (Scheme 1).

**Scheme 1:**
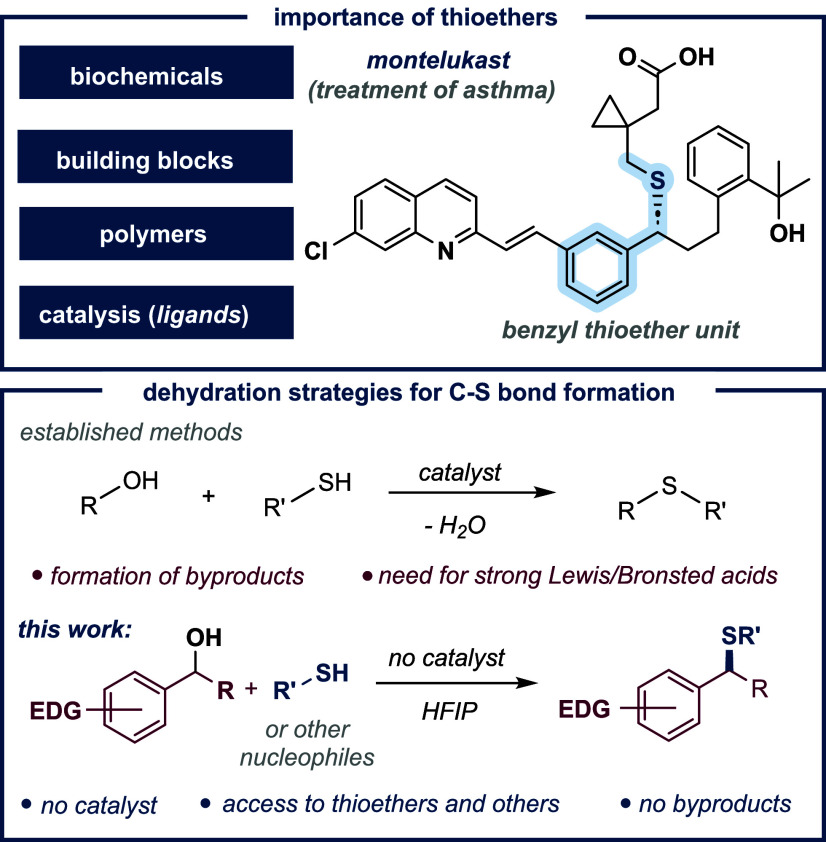
Context of the Investigation

Thus, we set out to investigate the use of HFIP in thioetherification in detail. Initially, we hypothesized that this simple solvent could facilitate the process, eliminating the need for d-block metals or highly acidic conditions. As a result, we developed a highly efficient method for thioether synthesis.

Moreover, this work addresses a key practical concern by offering an alternative strategy for synthesizing various trisubstituted methanes^32^ through nucleophilic substitution, utilizing activated arenes, allylsilanes, indoles, and other alcohol.

## Results and Discussion

In optimization studies, summarized in Table 1, we investigated the reaction of 1-(4-methoxyphenyl)­ethanol (**1a**) with thiophenol (**2a**). We selected **1a** due to its favorable electronic effects and the exceptional stability of its benzylic carbocation.^33^ The resulting carbocation is highly stabilized by resonance, facilitated by the electron-donating para-methoxy (−OCH_3_) group, which enhances charge delocalization and increases reaction efficiency. It should be strongly emphasized that the optimization studies were conducted using new glassware and new stir bars to eliminate the possibility of contamination.^34,35^ The first synthesis attempt was successful, confirming the hypothesis that thioethers can be formed without the presence of any external catalyst. This allowed us to establish the optimal reaction conditions (entry 1), yielding product **3a** with an excellent 99% isolated yield. Notably, no byproducts—such as phenyl disulfide (**3a’**) or the symmetric ether (**3a″**)—were detected. Lowering the temperature by 20 °C completely stopped the reaction, demonstrating the necessity of elevated temperatures (entry 2). The reaction proceeds equally well under an argon atmosphere (entry 3). Interestingly, the reaction also proceeded at 80 °C in other solvents (entries 4–8), including acetonitrile, nitromethane, 2-methyltetrahydrofuran (2-MeTHF), fluorobenzene, and dimethoxyethane (DME). However, these conditions led to significant formation of diphenyl sulfide as a byproduct (even under Ar atmosphere), complicating product isolation by necessitating column chromatography and large quantities of solvents.

**Table 1:**
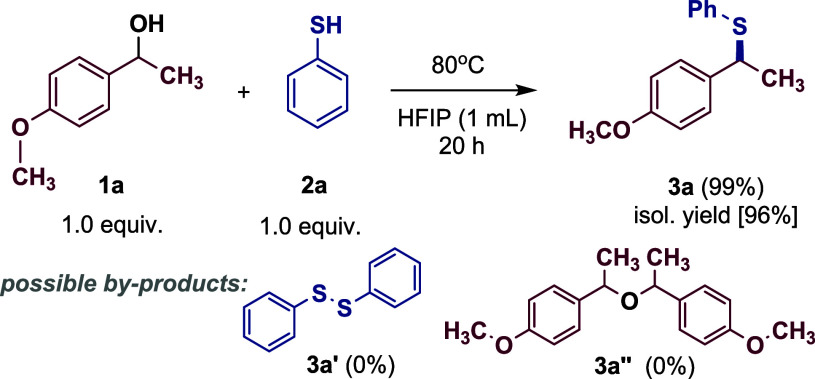
Optimization Studies for a Dehydrative Thioetherification of Alcohols^a^

Entry	Variation of conditions	Conversion of **2a** [%]	Selectivity [**3a**:**3a’**:**3a″**]^b^
1	no change	99	100:0: 0
2	in 60 °C	0	0:5: 0
3	under Ar	99	100:0: 0
4	in acetonitrile	99	86:14:0
5	in nitromethane	84	82:2: 16
6	in 2-MeTHF	99	96:4: 0
7	in fluorobenzene	99	95:5: 0
8	in DME	99	92:8: 0
9	neat	99	90:10:0

a General reaction conditions: **1a** (1.0 equiv., 1 mmol, 0.152 g), **2a** (1.0 equiv., 1 mmol, 0.11 g), **HFIP** (1 mL), under ambient atmosphere, 80 °C, 20 h.

b Ratio determined by GC-MS.

These results indicate that the role of HFIP is not solely due to its acidity, but rather its ability to stabilize reaction intermediates, which may modestly enhance the reaction efficiency. Moreover, the reaction can also proceed under solvent-free conditions (entry 9).

With the optimized reaction conditions established, we explored the scope of this dehydration process. The protocol demonstrated effectiveness across a variety of thiols, with no significant impact of steric or electronic effects on reaction efficiency (Scheme 2, part A). Accordingly, both aromatic (products **3a**–**3k**), heteroaromatic (products **3l** and **3m**) and aliphatic (products **3n**–**3t**) thiols can be used as nucleophiles, with the former being effective regardless of the substituents on the aryl and (het)­aryl rings. For example, product **3a** has so far been selectively obtained *via* dehydration exclusively using catalysts. which highlights the importance of precise optimization and analysis of various factors, such as the type of solvent and reaction temperature. Interestingly, as many as 11 of the obtained products during the scope study for thiols turned out to be previously unknown in the literature.

**Scheme 2:**
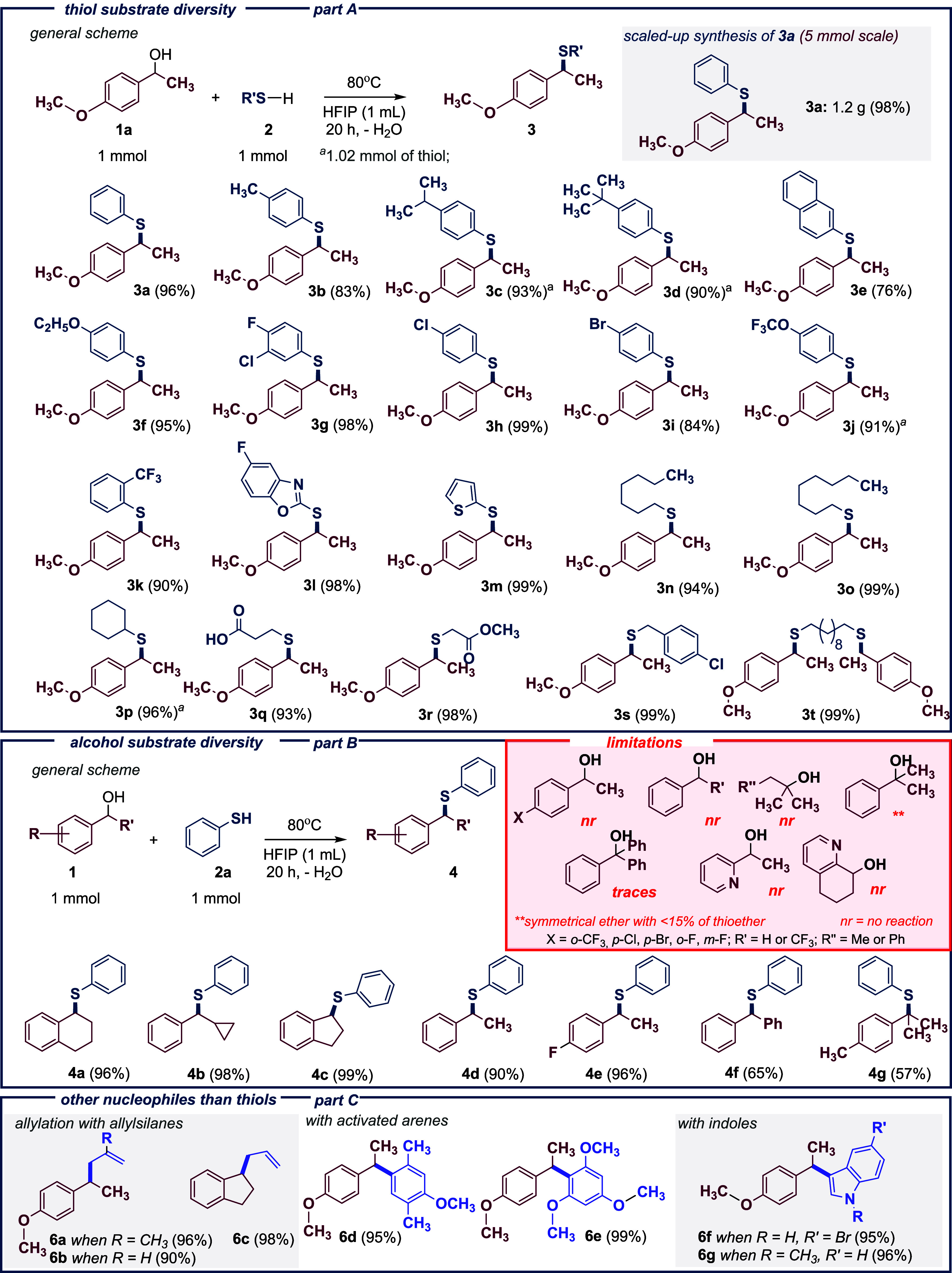
Substrate Scope for Dehydrative Nucleophilic Substitution

The situation is entirely different in the case of the scope study for alcohols (Scheme 2, part B). In this case, only secondary benzyl alcohols with electron-donating substituents can be successfully used for the synthesis of thioethers. The only exception to this rule is 1-(4-fluorophenyl)­ethanol (product **4e**). Fluorine, when substituted on a benzene ring, exhibits both σ-electron withdrawing and π-electron donating effects. This delicate interplay between inductive and resonance effects significantly influences reactivity. Notably, compounds with fluorine in the *para* position display exceptionally high reactivity due to fluorine’s strong ability to stabilize a positive charge at the opposite (*para*) position.^36,37^ In contrast, no reactions were observed for substrates with *meta-* and *ortho*-fluoro-substituted phenyl rings. A lack of reactivity was also observed for other secondary benzyl alcohols bearing chloro, bromo, and trifluoromethyl substituents, as well as for their corresponding pyridine derivatives. Similarly, no desired product was detected in the case of primary benzyl alcohol. In contrast, the reaction of tertiary 2-phenyl-2-propanol led to the formation of a mixture of products, with the symmetric ether being the major component. However, the analogous *p,α,α*-trimethylbenzyl alcohol was more selective and was subsequently transformed efficiently into the corresponding thioether **4g** with a 57% yield. The presence of an aryl ring proved to be essential for reactivity, as evidenced by the lack of reaction with the typically reactive tertiary *tert*-amyl alcohol. On the other hand, indane and tetrahydronaphthalene derivatives exhibited exceptionally high reactivity (products **4a** and **4c**). Moreover, 1-phenylethanol **(4d)**, and benzhydrol (**4f**) can also be transformed into the corresponding thioethers.

Given the formation of a stable secondary benzyl carbocation, we decided to explore the reactivity with other nucleophiles (Scheme 2, part C). To this end, we investigated the reactions of selected alcohols with allylsilanes (products **6a**–**6c**), activated arenes (products **6d** and **6e**), and indoles (products **6f** and **6g**). In each case, trisubstituted methanes (other than previous thioethers) were obtained with high yields. In the allylation process, the same set of reagents was tested using nitromethane instead of HFIP. In contrast to the reactions with thiols, no allylation products were observed in CH_3_NO_2_.

This undoubtedly demonstrates the greater role of HFIP in this transformation, where its enhanced ability to form hydrogen bonds plays a crucial role.^38^ It is worth emphasizing that, to date, reactions involving such substrates have mostly been carried out in the presence of acidic catalysts (e.g., B,^39^ Bi,^40^ In,^41^ *etc.*). To sum up, all these examples illustrate the significant potential of the developed reaction system and highlight the value of continuing future studies using hexafluoroisopropanol as both a solvent and/or a promoter.

Although the reaction mechanism (S_N_1-type nucleophilic substitution)^42^ is unambiguous in this case, we wanted to examine and highlight a few aspects further. First, as previously demonstrated in studies on the reaction scope (Scheme 2, part B), α-cyclopropylbenzyl alcohol undergoes the reaction, forming product **4b**. This reaction serves as an example of a radical clock experiment (Scheme 3, part A). No traces of byproducts indicating the cleavage of the cyclopropane ring were detected in the reaction mixture, which rules out the involvement of any free radicals.^43^ Finally, we considered whether it would also be possible to conduct the reaction of dehydrative etherification via direct coupling between two alcohols (Scheme 3, part B). Very recently, many interesting approaches have been developed, ranging from conventional catalysis^44–48^ to electrochemical methods.^49^ We assumed that, since a stable carbocation is formed, it should also be possible to use suitable alcohols as nucleophiles. To investigate this, we examined the reaction between **1a** and propargyl alcohol. As expected, the desired product **7a** was successfully obtained in the form of an unsymmetrical ether with a yield of 85%. Of course, the presented approach requires a thorough analysis, and the presented example is intended only to outline the subject of future research and demonstrate the great potential of using HFIP in synthesis involving alcohols.

**Scheme 3:**
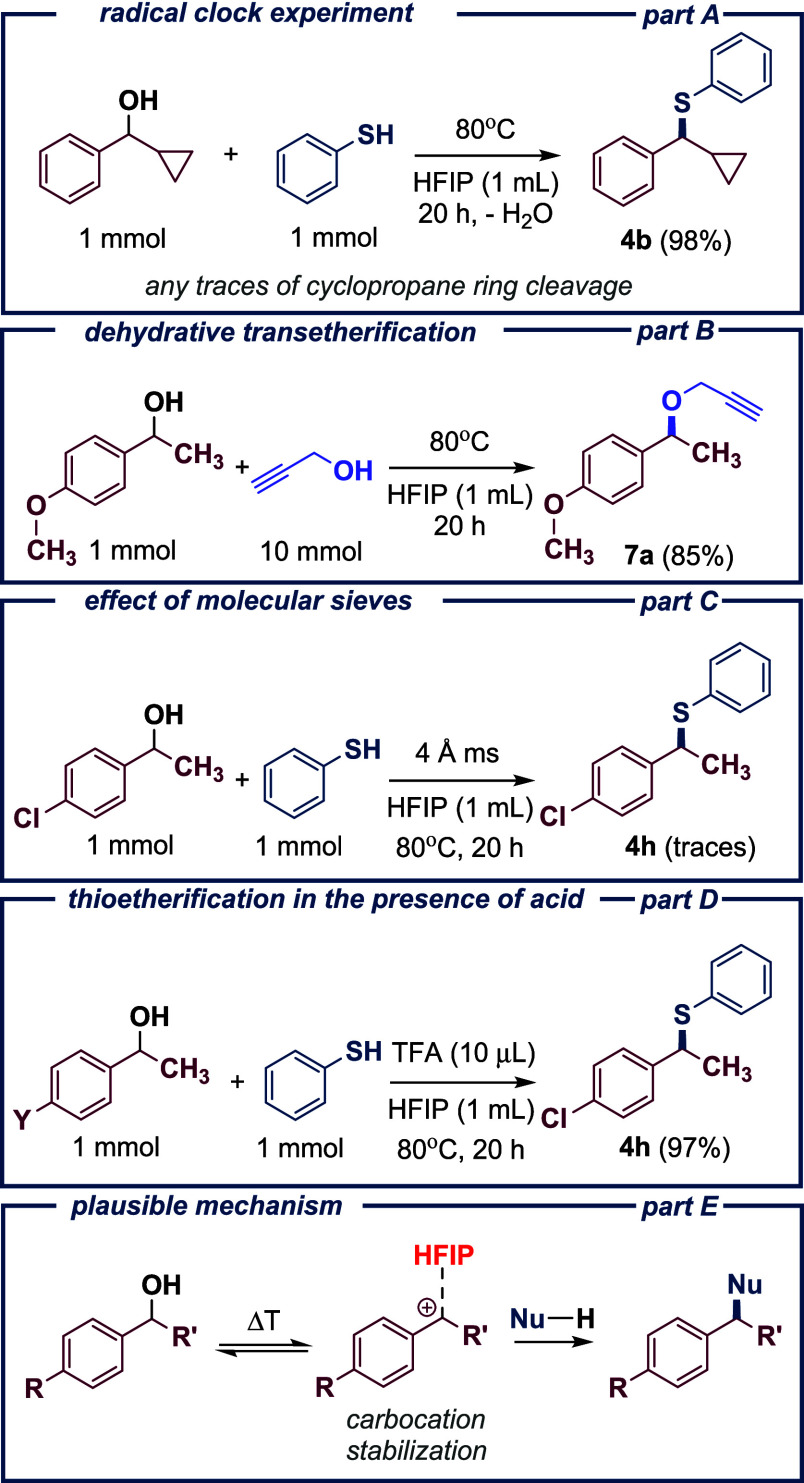
Further Investigations

Next, the reaction between 1-(4-chlorophenyl)­ethanol and thiophenol was reinvestigated in the presence of molecular sieves (Scheme 3, part C). The latter significantly accelerated dehydration due to their ability to adsorb water from the reaction system. However, despite the use of 4Å pore size sieves, only trace amounts of product **4h** were detected (less than 1%).

One of the central findings of this work is that a broad range of electron-rich secondary benzylic alcohols can be efficiently transformed into trisubstituted methanes. Notably, product **3a** was obtained in 96% yield under the optimized conditions, and in 98% yield on a gram scale. In comparison, previous reports employing catalytic systems achieved lower yields 91% using a Lewis acid catalyst (In)^26^ and 90% with a transition metal catalyst (Mo).^22^ Similarly, product **6e**, which had previously been synthesized via a vanadium-catalyzed protocol,^23^ was reported in 89% yield (99% under the presented conditions). These examples underscore the advantages of the present method in terms of eliminating the need for a catalyst, simplifying product isolation, and improving overall yields. Nevertheless, certain limitations of the methodology must be acknowledged. The key requirement for the effectiveness of our method is the ability to generate a stable secondary benzylic carbocation. Steric hindrance also plays a significant role; for example, in the case of benzhydrol (product **4f**), we observed incomplete conversion, which resulted in a lower yield. On the other hand, although tertiary benzylic carbocations are similarly stable, they proved to be significantly less selective, as they also underwent self-coupling reactions, leading to the formation of considerable amounts of symmetrical ethers. Importantly, benzylic alcohols bearing electron-withdrawing substituents were found to be unreactive under the standard conditions. To address this limitation, the effect of Brønsted acid additives was investigated with these less reactive substrates. In our previous work, we demonstrated that the thioetherification reaction proceeds with high selectivity in the presence of triflic acid. To gain further insight and explore more cost-effective alternatives, we decided to test trifluoroacetic acid (Scheme 3, part D). In the case of 1-(4-chlorophenyl)­ethanol, complete conversion (100%) of the alcohol was observed within 30 min of reaction initiation, leading to the formation of product **4h**, bearing electron-withdrawing substituent.

Based on the results obtained from the reaction scope, together with previously reported data, a mechanism involving the formation of a carbocation intermediate via an S_N_1 pathway has been proposed, with additional stabilization of the cationic species in the presence of HFIP (Scheme 3, part E).

## Conclusion

In summary, we have developed a practical and inexpensive method for the synthesis of several trisubstituted methanes including thioethers, allylic compounds, substituted indoles, *etc.* No catalyst is required for this synthesis, and in most cases, isolating the final products involved only the evaporation of volatile components from the reaction mixture. This method further highlights HFIP as an exceptional solvent, enabling the development of a highly efficient process.

## Supplementary Material



## Data Availability

The data underlying this study are available in the published article and its Supporting Information.

## Notes

Experimental section, characterization of the obtained products, their spectra, and the authors have cited additional references within the Supporting Information^50–56^ (PDF)

## References

[1] Estopiñá-Durán S., Taylor J. E. (2021). Brønsted Acid-Catalysed Dehydrative Substitution
Reactions of Alcohols. Chem.–Eur. J..

[2] Trillo P., Baeza A., Nájera C. (2012). Fluorinated
Alcohols As Promoters
for the Metal-Free Direct Substitution Reaction of Allylic Alcohols
with Nitrogenated, Silylated, and Carbon Nucleophiles. J. Org. Chem..

[3] Motiwala H. F., Armaly A. M., Cacioppo J. G., Coombs T. C., Koehn K. R. K., Norwood V. M. I., Aubé J. (2022). HFIP in Organic
Synthesis. Chem. Rev..

[4] Ghosh S., Patra K., Baidya M. (2024). Allure of
HFIP in Unsaturated Carbon–Carbon
Bond Functionalization. Eur. J. Org. Chem..

[5] Pozhydaiev V., Al-Othman D., Moran J., Lebœuf D. (2024). A Povarov-Type
Reaction to Access Tetrahydroquinolines from N-Benzylhydroxylamines
and Alkenes in HFIP. Chem. Commun..

[6] Piejko M., Moran J., Lebœuf D. (2024). Difunctionalization
Processes Enabled
by Hexafluoroisopropanol. ACS Org. Inorg. Au..

[7] Frank N., Leutzsch M., List B. (2025). Bro̷nsted Acid-Catalyzed Reduction
of Furans. J. Am. Chem. Soc..

[8] Van
Hoof M., Mayer R. J., Moran J., Lebœuf D. (2025). Triflic Acid-Catalyzed
Dehydrative Amination of 2-Arylethanols with Weak N-Nucleophiles in
Hexafluoroisopropanol. Angew. Chem., Int. Ed..

[9] Xu H., Luo H., Wang Y., Gao H. (2025). Research Progress of Homogeneous
Catalytic Reactions of Hexafluoroisopropanol (HFIP). Tetrahedron Green Chem..

[10] Zheng Y., Liu Z., Huang Q., Xie Y. (2025). Ipso-Nitration of Boronic Esters
Enabled by Ferric Nitrate Nonahydrate (Fe­(NO_3_)_3_·9H_2_O) in HFIP. Org. Lett..

[11] Das A., Senapati S. K. (2025). Reductive
Alkylation of Azoarenes to N-Alkylated Hydrazines
Enabled by Hexafluoroisopropanol. Chem. Commun..

[12] Colomer I., Batchelor-McAuley C., Odell B., Donohoe T. J., Compton R. G. (2016). Hydrogen
Bonding to Hexafluoroisopropanol Controls the Oxidative Strength of
Hypervalent Iodine Reagents. J. Am. Chem. Soc..

[13] Barp M., Kreuter F., Huang Q.-R., Jin J., Ninov F. E., Kuo J.-L., Tonner-Zech R., Asmis K. R. (2025). Quantifying Hexafluoroisopropanol’s
Hydrogen Bond Donor Ability: Infrared Photodissociation Spectroscopy
of Halide Anion HFIP Complexes. Chem. Sci..

[14] Although HFIP is not classified as highly toxic, appropriate caution should be taken when handling this solvent, as it can be hazardous, particularly to the eyes and skin.

[15] Rzayev R., Niyazova A. A., Karimova N. K., Suleymanova A. E. (2024). Recent
Advances in Transition-Metal-Catalyzed Dehydrative Thioetherification
of Alcohols with Thiols. Chem. Rev. Lett..

[16] Li P., Yang Y., Wang X., Wu X. (2021). Recent Achievements
on the Agricultural Applications of Thioether Derivatives: A 2010–2020
Decade in Review. J. Heterocycl. Chem..

[17] Geiger V.
J., Oechsner R. M., Gehrtz P. H., Fleischer I. (2022). Recent Metal-Catalyzed
Methods for Thioether Synthesis. Synthesis.

[18] Beletskaya I. P., Ananikov V. P. (2022). Transition-Metal-Catalyzed
C–S, C–Se,
and C–Te Bond Formations via Cross-Coupling and Atom-Economic
Addition Reactions Achievements And Challenges. Chem. Rev..

[19] Bellemin-Laponnaz S., Achard T. (2024). Recent Progress in Developing Thioether-Containing
Ligands for Catalysis Applications. Synthesis.

[20] Kang B., Wei L., Jiang H., Qi C. (2025). Cyclic Diphenylchloronium-Salt-Triggered
Coupling of Sulfides with Nucleophiles: Modular Assembly of Pharmaceuticals. Org. Lett..

[21] Sorribes I., Corma A. (2019). Nanolayered Cobalt–Molybdenum
Sulphides (Co–Mo–S)
Catalyse Borrowing Hydrogen C–S Bond Formation Reactions of
Thiols or H_2_S with Alcohols. Chem.
Sci..

[22] Singh R. R., Whittington A., Srivastava R. S. (2020). Molybdenum (VI)-Catalyzed Dehydrative
Construction of CO and CS Bonds Formation via Etherification and Thioetherification
of Alcohols and Thiols. Mol. Catal..

[23] Nishio T., Yoshioka S., Hasegawa K., Yahata K., Kanomata K., Akai S. (2021). Direct Nucleophilic
Substitution of Alcohols Using an Immobilized
Oxovanadium Catalyst. Eur. J. Org. Chem..

[24] Rodenes M., Dhaeyere F., Martín S., Concepción P., Corma A., Sorribes I. (2023). Multifunctional Catalysis of Nanosheet
Defective Molybdenum Sulfide Basal Planes for Tandem Reactions Involving
Alcohols and Molecular Hydrogen. ACS Sustain.
Chem. Eng..

[25] Rodenes M., Oštrić D., Martín S., Concepción P., Corma A., Sorribes I. (2025). Alloying Engineering
of Defective
Molybdenum Sulfide Basal Planes for Enhanced Borrowing Hydrogen Activity
in the Thioetherification of Alcohols. ChemSusChem.

[26] Kuciński K., Hreczycho G. (2017). Highly Efficient
and Chemoselective Tertiary and Secondary
Benzylation of Thiols Catalyzed by Indium­(III) Triflate. Eur. J. Org. Chem..

[27] Xu B., Lin Y., Ye Y., Xu L., Xie T., Ye X.-Y. (2021). Benzyl
Thioether Formation Merging Copper Catalysis. RSC Adv.

[28] Markwitz M., Labrzycki K., Azcune L., Landa A., Kuciński K. (2023). Access to
Thioethers from Thiols and Alcohols via Homogeneous and Heterogeneous
Catalysis. Sci. Rep..

[29] Wang Z., Hu X., Song C., Su H., Xiong C., Chen G., Bao X. (2025). An Alcohol Thioetherification
Method Utilizing a Domino Dual Catalysis
Strategy. Tetrahedron Lett..

[30] Li J., Ma J., Wei C., Zheng Z., Han Y., Wang H., Wang X., Hu C. (2024). Polyoxometalate-Based
Ionic Liquids:
Efficient Reversible Phase Transformation-Type Catalysts for Thiolation
of Alcohols to Construct C–S Bonds. Dalton
Trans..

[31] Zhu Y., Yang C., Lin Q., Li J., Loh T.-P., Chen P., Jia Z. (2025). Rapid C–S Coupling in Water
via Ion-Pair-Catalyzed Dehydration. Org. Lett..

[32] Verma M., Thakur A., Sharma R., Bharti R. (2022). Recent Advancement
in the One-Pot Synthesis of the Tri-Substituted Methanes (TRSMs) and
Their Biological Applications. Curr. Org. Synth..

[33] Santoro F., Mariani M., Zaccheria F., Psaro R., Ravasio N. (2016). Selective
Synthesis of Thioethers in the Presence of a Transition-Metal-Free
Solid Lewis Acid. Beilstein J. Org. Chem..

[34] Pentsak E. O., Eremin D. B., Gordeev E. G., Ananikov V. P. (2019). Phantom Reactivity
in Organic and Catalytic Reactions as a Consequence of Microscale
Destruction and Contamination-Trapping Effects of Magnetic Stir Bars. ACS Catal..

[35] Sau S., Pramanik M., Bal A., Mal P. (2022). Reported Catalytic
Hydrofunctionalizations That Proceed in the Absence of Catalysts:
The Importance of Control Experiments. Chem.
Rec..

[36] Rosenthal J., Schuster D. I. (2003). The Anomalous Reactivity of Fluorobenzene in Electrophilic
Aromatic Substitution and Related Phenomena. J. Chem. Educ..

[37] Carroll T. X., Thomas T. D., Bergersen H., Børve K. J., Sæthre J. L. (2006). Fluorine as a π Donor. Carbon 1s Photoelectron
Spectroscopy and Proton Affinities of Fluorobenzenes. J. Org. Chem..

[38] Panchenko S. P., Runichina S. A., Tumanov V. V. (2011). The Hosomi–Sakurai Allylation
in Hexafluoroisopropanol: Solvent Promotion Effect. Mendeleev Commun..

[39] Estopiñá-Durán S., Mclean E. B., Donnelly L. J., Hockin B. M., Taylor J. E. (2020). Arylboronic
Acid Catalyzed C-Alkylation and Allylation Reactions Using Benzylic
Alcohols. Org. Lett..

[40] Kumar G. G. K. S. N., Laali K. K. (2012). Facile Coupling
of Propargylic, Allylic and Benzylic
Alcohols with Allylsilane and Alkynylsilane, and Their Deoxygenation
with Et_3_SiH, Catalyzed by Bi­(OTf)_3_ in [BMIM]­[BF_4_] Ionic Liquid (IL), with Recycling and Reuse of the IL. Org. Biomol. Chem..

[41] Saito T., Nishimoto Y., Yasuda M., Baba A. (2006). Direct Coupling
Reaction
between Alcohols and Silyl Compounds: Enhancement of Lewis Acidity
of Me_3_SiBr Using InCl_3_. J. Org. Chem..

[42] Emer E., Sinisi R., Capdevila M. G., Petruzziello D., Vincentiis F. D., Cozzi P. G. (2011). Direct Nucleophilic
SN1-Type Reactions
of Alcohols. Eur. J. Org. Chem..

[43] Hikawa H., Mori Y., Kikkawa S., Azumaya I. (2016). A Radical Pathway for
Direct Substitution of Benzyl Alcohols with Water-Soluble Copper Catalyst
in Water. Adv. Synth. Catal..

[44] Das K., Yasmin E., Kumar A. (2022). Pincer-Ruthenium
Catalyzed Oxygen
Mediated Dehydrative Etherification of Alcohols and Ortho-Alkylation
of Phenols. Adv. Synth. Catal..

[45] Miyauchi M., Hiraoka T., Raut V. S., Asao N. (2023). Photocatalytic Dehydrative
Etherification of Alcohols with a Nanoporous Gold Catalyst. Chem. Commun..

[46] Slimi H., Litim Z., Ollevier T., Kraïem J. (2023). Eco-Friendly
Homo- and Cross-Etherification of Benzyl Alcohols Catalyzed by Iron­(II/III)
Chloride in Propylene Carbonate as a Green and Recyclable Solvent. ACS Omega.

[47] Hashimoto T., Matsunaga Y., Okamura Y., Takao S., Hojo M. (2024). Alkoxyhydrosilane-Facilitated
Cross-Etherification Reaction of Secondary Benzyl Alcohol with Aliphatic
Alcohol: Synthesis of Unsymmetrical Dialkyl Ethers. RSC Adv..

[48] Liu C., Liang J., Liang Y., Ouyang L., Li Y. (2025). Adaptive Alcohols-Alcohols
Cross-Coupling via TFA Catalysis: Access of Unsymmetrical Ethers. BMC Chem..

[49] Kiaku C., Kaltenberger S., Raydan D., Morlacci V., Claringbold B., Goodall C. A. I., Palombi L., Poole D. L., Lam K. (2025). eEtherification:
An Electrochemical Strategy toward the Synthesis of Sterically Hindered
Dialkyl Ethers from Activated Alcohols. Org.
Lett..

[50] Yi H., Song C., Li Y., Pao C.-W., Lee J.-F., Lei A. (2016). Single-Electron Transfer between CuX_2_ and Thiols Determined
by Extended X-Ray Absorption Fine Structure Analysis: Application
in Markovnikov-Type Hydrothiolation of Styrenes. Chem.–Eur. J..

[51] Ding Q., Cao B., Yuan J., Liu X., Peng Y. (2011). Synthesis of Thioethers
via Metal-Free Reductive Coupling of Tosylhydrazones with Thiols. Org. Biomol. Chem..

[52] Richard J. P., Jencks W. P. (1984). Reactions of Substituted
1-Phenylethyl Carbocations
with Alcohols and Other Nucleophilic Reagents. J. Am. Chem. Soc..

[53] Lehmkuhl H., McLane R. (1980). Additionen von Organozink
verbindungen an C = C-Bindungen,
III. Anlagerung von Bis­(2-methylallyl)­zink an para - an meta substituierte
Styrole. Liebigs Ann. Chem..

[54] Croft R. A., Mousseau J. J., Choi C., Bull J. A. (2018). Lithium-Catalyzed
Thiol Alkylation with Tertiary and Secondary Alcohols: Synthesis of
3-Sulfanyl-Oxetanes as Bioisosteres. Chem.–Eur.
J..

[55] Radtke M. A., Lambert T. H. (2018). Silylated Cyclopentadienes
as Competent Silicon Lewis
Acid Catalysts. Chem. Sci..

[56] Talluri S. K., Sudalai A. (2005). NBS-Catalyzed Hydroamination
and Hydroalkoxylation
of Activated Styrenes. Org. Lett..

