# Supplementary material for: Functionalization of Electron-Rich Secondary Benzyl Alcohols in HFIP: From Thioethers to Trisubstituted Methanes
Source: J Org Chem. 2025 Jun 28;90(27):9513–8. doi: 10.1021/acs.joc.5c00900 (PMC12261332; doi:10.1021/acs.joc.5c00900)
Supplement: Supplementary file 1 [file jo5c00900_si_001.pdf]

Supporting Information

Functionalization of Electron-Rich Secondary Benzyl Alcohols in HFIP: From Thioethers to Trisubstituted Methanes

Martyna Markwitz,[a] Klaudiusz Labrzycki,[a] Kacper Łyczek,[a] and Krzysztof Kuciński[a,*]

[a]Faculty of Chemistry, Adam Mickiewicz University, Poznań; Uniwersytetu Poznańskiego St. 8, 61-614 Poznań (Poland);

*email: kucinski.k@amu.edu.pl;

Twitter account: @SiRCH_Kucina (KK)

https://www.kucinskilab.com/

ORCID iDs:

0009-0005-3251-6858 (Martyna Markwitz, marmar80@st.amu.edu.pl)

0009-0005-6972-0334 (Klaudiusz Labrzycki, klalab@st.amu.edu.pl)

0009-0006-1549-499X (Kacper Łyczek, kaclyc@st.amu.edu.pl)

0000-0002-9339-6395 (Krzysztof Kuciński, kucinski.k@amu.edu.pl)

Content

GENERAL INFORMATION S6

OPTIMIZATION OF REACTION CONDITIONS S7

Table S1. Optimization studies for a dehydrative thioetherification of alcohols S7

EXPERIMENTAL SECTION S8

Synthesis of compounds **3a**, **3b**, **3e-3i**, **3k-3o**, and **3q-3s** S8

Synthesis of compounds **3c**, **3d**, **3j** and **3p** S8

Synthesis of compound **3t** S8

Synthesis of compounds **4a-4e** S8

Synthesis of compounds **4f-4g** S8

Synthesis of compounds **4h** S9

Synthesis of compounds **6a-6c** S9

Synthesis of compounds **6d** and **6e** S9

Synthesis of compounds **6f** and **6**g S9

Synthesis of compound **7a** S9

Scaled-up synthesis of **3a** S10

CHARACTERIZATION DATA FOR ALL PRODUCTS S11

(1-(4-Methoxyphenyl)ethyl)(phenyl)sulfane (**3a**) S11

(1-(4-Methoxyphenyl)ethyl)(*p*-tolyl)sulfane (**3b**) S11

1-(4-Isopropylphenyl)(1-(4-methoxyphenyl)ethyl)sulfane (**3c**) S11

(4-(*Tert*-butyl)phenyl)(1-(4-methoxyphenyl)ethyl)sulfane (**3d**) S12

(1-(4-Methoxyphenyl)ethyl)(naphthalen-2-yl)sulfane (**3e**) S12

(4-Ethoxyphenyl)(1-(4-methoxyphenyl)ethyl)sulfane (**3f**) S12

(3-Chloro-4-fluorophenyl)(1-(4-methoxyphenyl)ethyl)sulfane (**3g**) S13

(4-Chlorophenyl)(1-(4-methoxyphenyl)ethyl)sulfane (**3h**) S13

(4-Bromophenyl)(1-(4-methoxyphenyl)ethyl)sulfane (**3i**) S13

(1-(4-Methoxyphenyl)ethyl)(4-(trifluoromethoxy)phenyl)sulfane (**3j**) S14

(1-(4-Methoxyphenyl)ethyl)(2-(trifluoromethyl)phenyl)sulfane (**3k**) S14

5-Fluoro-2-((1-(4-methoxyphenyl)ethyl)thio)benzo[d]oxazole (**3l**) S15

2-((1-(4-Methoxyphenyl)ethyl)thio)thiophene (**3m**) S15

Heptyl(1-(4-methoxyphenyl)ethyl)sulfane (**3n**) S15

(1-(4-Methoxyphenyl)ethyl)(octyl)sulfane (**3o**) S16

Cyclohexyl(1-(4-methoxyphenyl)ethyl)sulfane (**3p**) S16

3-((1-(4-Methoxyphenyl)ethyl)thio)propanoic acid (**3q**) S17

Methyl 2-((1-(4-methoxyphenyl)ethyl)thio)acetate (**3r**) S17

(4-Chlorobenzyl)(1-(4-methoxyphenyl)ethyl)sulfane (**3s**) S17

1-(1-(4-Methoxyphenyl)ethyl)thio)-10-(1-(4-methoxyphenyl)ethyl)thio)decane (**3t**) S18

Phenyl(1,2,3,4-tetrahydronaphthalen-1-yl)sulfane (**4a**) S18

(Cyclopropyl(phenyl)methyl)(phenyl)sulfane (**4b**) S18

(2,3-Dihydro-1H-inden-1-yl)(phenyl)sulfane (**4c**) S19
Phenyl(1-phenylethyl)sulfane (**4d**) S19
(1-(4-Fluorophenyl)ethyl)(phenyl)sulfane (**4e**) S19
Benzhydryl(phenyl)sulfane (**4f**) S20
Phenyl(2-(*p*-tolyl)propan-2-yl)sulfane (**4g**) S20
(1-(4-Chlorophenyl)ethyl)(phenyl)sulfane (**4h**) S20
1-Methoxy-4-(4-methylpent-4-en-2-yl)benzene (**6a**) S21
1-Methoxy-4-(pent-4-en-2-yl)benzene (**6b**) S21
1-Allyl-2,3-dihydro-1H-indene (**6c**) S21
1-Methoxy-4-(1-(4-methoxyphenyl)ethyl)-2,5-dimethylbenzene (**6d**) S22
1,3,5-Trimethoxy-2-(1-(4-methoxyphenyl)ethyl)benzene (**6e**) S22
5-Bromo-3-(1-(4-methoxyphenyl)ethyl)-*1H*-indole (**6f**) S22
3-(1-(4-Methoxyphenyl)ethyl)-1-methyl-*1H*-indole (**6g**) S23
1-Methoxy-4-(1-(prop-2-yn-1-yloxy)ethyl)benzene (**7a**) S23
SPECTRA FOR ALL PRODUCTS S24
(1-(4-Methoxyphenyl)ethyl)(phenyl)sulfane (**3a**) S25
Figure S1. ^{1}H NMR (400 MHz, Chloroform-d, 25°C) of (1-(4-methoxyphenyl)ethyl)(phenyl)sulfane (**3a**). S25
Figure S2. ^{13}C{^{1}H} NMR (101 MHz, Chloroform-d, 25°C) of (1-(4-methoxyphenyl)ethyl)(phenyl) (**3a**). S25
(1-(4-Methoxyphenyl)ethyl)(*p*-tolyl)sulfane (**3b**) S26
Figure S3. ^{1}H NMR (400 MHz, Chloroform-d, 25°C) of (1-(4-methoxyphenyl)ethyl)(*p*-tolyl)sulfane (**3b**). S26
Figure S4. ^{13}C{^{1}H} NMR (101 MHz, Chloroform-d, 25°C) of (1-(4-methoxyphenyl)ethyl)(*p*-tolyl)sulfane (**3b**). S26
1-(4-Isopropylphenyl)(1-(4-methoxyphenyl)ethyl)sulfane (**3c**) S27
Figure S5. ^{1}H NMR (400 MHz, Chloroform-d, 25°C) of 1-(4-isopropylphenyl)(1-(4-methoxyphenyl)ethyl)sulfane (**3c**). S27
Figure S6. ^{13}C{^{1}H} NMR (101 MHz, Chloroform-d, 25°C) of 1-(4-isopropylphenyl)(1-(4-methoxyphenyl)ethyl)sulfane (**3c**). S27
(4-(*Tert*-butyl)phenyl)(1-(4-methoxyphenyl)ethyl)sulfane (**3d**) S28
Figure S7. ^{1}H NMR (400 MHz, Chloroform-d, 25°C) of (4-(*tert*-butyl)phenyl)(1-(4-methoxyphenyl)ethyl)sulfane (**3d**). S28
Figure S8. ^{13}C{^{1}H} NMR (101 MHz, Chloroform-d, 25°C) of (4-(*tert*-butyl)phenyl)(1-(4-methoxyphenyl)ethyl)sulfane (**3d**). S28
(1-(4-Methoxyphenyl)ethyl)(naphthalen-2-yl)sulfane (**3e**) S29
Figure S9. ^{1}H NMR (400 MHz, Chloroform-d, 25°C) of (1-(4-methoxyphenyl)ethyl)(naphthalen-2-yl)sulfane (**3e**) S29
Figure S10. ^{13}C{^{1}H} NMR (101 MHz, Chloroform-d, 25°C) of (1-(4-Methoxyphenyl)ethyl)(naphthalen-2-yl)sulfane (**3e**) S29
(4-Ethoxyphenyl)(1-(4-methoxyphenyl)ethyl)sulfane (**3f**) S30
Figure S11. ^{1}H NMR (400 MHz, Chloroform-d, 25°C) of 1(4-ethoxyphenyl)(1-(4-methoxyphenyl)ethyl)sulfane (**3f**). S30
Figure S12. ^{13}C{^{1}H} NMR (101 MHz, Chloroform-d, 25°C) of 1(4-ethoxyphenyl)(1-(4-methoxyphenyl)ethyl)sulfane (**3f**). S30
(3-Chloro-4-fluorophenyl)(1-(4-methoxyphenyl)ethyl)sulfane (**3g**) S31
Figure S13. ^{1}H NMR (400 MHz, Chloroform-d, 25°C) of (3-chloro-4-fluorophenyl)(1-(4-methoxyphenyl)ethyl)sulfane (**3g**). S31
Figure S14. ^{13}C{^{1}H} NMR (101 MHz, Chloroform-d, 25°C) of of (3-chloro-4-fluorophenyl)(1-(4-methoxyphenyl)ethyl)sulfane (**3g**). S31
Figure S15. ^{19}F NMR (377 MHz, Chloroform-d, 25°C) of of (3-chloro-4-fluorophenyl)(1-(4-methoxyphenyl)ethyl)sulfane (**3g**). S32
(4-Chlorophenyl)(1-(4-methoxyphenyl)ethyl)sulfane (**3h**) S33
Figure S16. ^{1}H NMR (400 MHz, Chloroform-d, 25°C) of (4-chlorophenyl)(1-(4-methoxyphenyl)ethyl)sulfane (**3h**). S33
Figure S17. ^{13}C{^{1}H} NMR (101 MHz, Chloroform-d, 25°C) of (4-chlorophenyl)(1-(4-methoxyphenyl)ethyl)sulfane (**3h**). S33
(4-Bromophenyl)(1-(4-methoxyphenyl)ethyl)sulfane (**3i**) S34
Figure S18. ^{1}H NMR (400 MHz, Chloroform-d, 25°C) of (4-bromophenyl)(1-(4-methoxyphenyl)ethyl)sulfane (**3i**). S34
Figure S19. ^{13}C{^{1}H} NMR (101 MHz, Chloroform-d, 25°C) of (4-bromophenyl)(1-(4-methoxyphenyl)ethyl)sulfane (**3i**). S34

(1-(4-Methoxyphenyl)ethyl)(4-(trifluoromethoxy)phenyl)sulfane (**3j**) S35
Figure S20. ^{1}H NMR (400 MHz, Chloroform-d, 25°C) of (1-(4-methoxyphenyl)ethyl)(4-(trifluoromethoxy)phenyl)sulfane (**3j**). S35
Figure S21. ^{13}C{^{1}H} NMR (101 MHz, Chloroform-d, 25°C) of (1-(4-methoxyphenyl)ethyl)(4-(trifluoromethoxy)phenyl)sulfane (**3j**). S35
Figure S22. ^{19}F NMR (377 MHz, Chloroform-d, 25°C) of (1-(4-methoxyphenyl)ethyl)(4-(trifluoromethoxy)phenyl)sulfane (**3j**). S36
(1-(4-Methoxyphenyl)ethyl)(2-(trifluoromethyl)phenyl)sulfane (**3k**) S37
Figure S23. ^{1}H NMR (400 MHz, Chloroform-d, 25°C) of (1-(4-methoxyphenyl)ethyl)(2-(trifluoromethyl)phenyl)sulfane (**3k**). S37
Figure S24. ^{13}C{^{1}H} NMR (101 MHz, Chloroform-d, 25°C) of (1-(4-Methoxyphenyl)ethyl)(2-(trifluoromethyl)phenyl)sulfane (**3k**). S37
Figure S25. ^{19}F NMR (377 MHz, Chloroform-d, 25°C) of (1-(4-Methoxyphenyl)ethyl)(2-(trifluoromethyl)phenyl)sulfane (**3k).** S38
5-Fluoro-2-((1-(4-methoxyphenyl)ethyl)thio)benzo[d]oxazole (**3l**) S39
Figure S26. ^{1}H NMR (400 MHz, Chloroform-d, 25°C) of 5-fluoro-2-((1-(4-methoxyphenyl)ethyl)thio)benzo[d]oxazole (**3l**). S39
Figure S27. ^{13}C{^{1}H} NMR (101 MHz, Chloroform-d, 25°C) of 5-fluoro-2-((1-(4-methoxyphenyl)ethyl)thio)benzo[d]oxazole (**3l**). S39
Figure S28. ^{19}F NMR (377 MHz, Chloroform-d, 25°C) of 5-fluoro-2-((1-(4-methoxyphenyl)ethyl)thio)benzo[d]oxazole (**3l**). S40
2-((1-(4-Methoxyphenyl)ethyl)thio)thiophene (**3m**) S41
Figure S29. ^{1}H NMR (400 MHz, Chloroform-d, 25°C) of 2-((1-(4-methoxyphenyl)ethyl)thio)thiophene (**3m**). S41
Figure S30. ^{13}C{^{1}H} NMR (101 MHz, Chloroform-d, 25°C) of 2-((1-(4-methoxyphenyl)ethyl)thio)thiophene (**3m**). S41
Heptyl(1-(4-methoxyphenyl)ethyl)sulfane (**3n**) S42
Figure S31. ^{1}H NMR (400 MHz, Chloroform-d, 25°C) of heptyl(1-(4-methoxyphenyl)ethyl)sulfane (**3n**). S42
Figure S32. ^{13}C{^{1}H} NMR (101 MHz, Chloroform-d, 25°C) of heptyl(1-(4-methoxyphenyl)ethyl)sulfane (**3n**). S42
(1-(4-Methoxyphenyl)ethyl)(octyl)sulfane (**3o**) S43
Figure S33. ^{1}H NMR (400 MHz, Chloroform-d, 25°C) of (1-(4-methoxyphenyl)ethyl)(octyl)sulfane (**3o**). S43
Figure S34. ^{13}C{^{1}H} NMR (101 MHz, Chloroform-d, 25°C) of (1-(4-methoxyphenyl)ethyl)(octyl)sulfane (**3o**). S43
Cyclohexyl(1-(4-methoxyphenyl)ethyl)sulfane (**3p**) S44
Figure S35. ^{1}H NMR (400 MHz, Chloroform-d, 25°C) of cyclohexyl(1-(4-methoxyphenyl)ethyl)sulfane (**3p**). S44
Figure S36. ^{13}C{^{1}H} NMR (101 MHz, Chloroform-d, 25°C) of cyclohexyl(1-(4-methoxyphenyl)ethyl)sulfane (**3p**). S44
3-((1-(4-Methoxyphenyl)ethyl)thio)propanoic acid (**3q**) S45
Figure S37. ^{1}H NMR (400 MHz, Chloroform-d, 25°C) of 3-((1-(4-methoxyphenyl)ethyl)thio)propanoic acid (**3q**). S45
Figure S38. ^{13}C{^{1}H} NMR (101 MHz, Chloroform-d, 25°C) of 3-((1-(4-methoxyphenyl)ethyl)thio)propanoic acid (**3q**). S45
Methyl 2-((1-(4-methoxyphenyl)ethyl)thio)acetate (**3r**) S46
Figure S39. ^{1}H NMR (400 MHz, Chloroform-d, 25°C) of methyl 2-((1-(4-methoxyphenyl)ethyl)thio)acetate (**3r**). S46
Figure S40. ^{13}C{^{1}H} NMR (101 MHz, Chloroform-d, 25°C) of methyl 2-((1-(4-methoxyphenyl)ethyl)thio)acetate (**3r**). S46
(4-Chlorobenzyl)(1-(4-methoxyphenyl)ethyl)sulfane (**3s**) S47
Figure S41. ^{1}H NMR (400 MHz, Chloroform-d, 25°C) of (4-chlorobenzyl)(1-(4-methoxyphenyl)ethyl)sulfane (**3s**). S47
Figure S42. ^{13}C{^{1}H} NMR (101 MHz, Chloroform-d, 25°C) of (4-chlorobenzyl)(1-(4-methoxyphenyl)ethyl)sulfane (**3s**). S47
1-(1-(4-Methoxyphenyl)ethyl)thio)-10-(1-(4-methoxyphenyl)ethyl)thio)decane (**3t**) S48
Figure S43. ^{1}H NMR (400 MHz, Chloroform-d, 25°C) of 1-(1-(4-methoxyphenyl)ethyl)thio)-10-(1-(4-methoxyphenyl)ethyl)thio)decane (**3t**). S48
Figure S44. ^{13}C{^{1}H} NMR (101 MHz, Chloroform-d, 25°C) of 1-(1-(4-methoxyphenyl)ethyl)thio)-10-(1-(4-methoxyphenyl)ethyl)thio)decane (**3t**). S48
PheSnyl(1,2,3,4-tetrahydronaphthalen-1-yl)sulfane (**4a**) S49
FigSure S45. ^{1}H NMR (400 MHz, Chloroform-d, 25°C) of phenyl(1,2,3,4-tetrahydronaphthalen-1-yl)sulfane (**4a**). S49
FiguSre S46. ^{13}C{^{1}H} NMR (101 MHz, Chloroform-d, 25°C) of phenyl(1,2,3,4-tetrahydronaphthalen-1-yl)sulfane (**4a**). S49
(Cyclopropyl(phenyl)methyl)(phenyl)sulfane (**4b**) S50
Figure S47. ^{1}H NMR (400 MHz, Chloroform-d, 25°C) of (cyclopropyl(phenyl)methyl)(phenyl)sulfane (**4b**). S50
Figure S48. ^{13}C{^{1}H} NMR (101 MHz, Chloroform-d, 25°C) of (cyclopropyl(phenyl)methyl)(phenyl)sulfane (**4b**). S50
(2,3-Dihydro-1H-inden-1-yl)(phenyl)sulfane (**4c**) S51

Figure S49. ^{1}H NMR (400 MHz, Chloroform-d, 25°C) of (2,3-dihydro-1H-inden-1-yl)(phenyl)sulfane (**4c**). S51
Figure S50. ^{13}C{^{1}H} NMR (101 MHz, Chloroform-d, 25°C) of (2,3-dihydro-1H-inden-1-yl)(phenyl)sulfane (**4c**). S51
Phenyl(1-phenylethyl)sulfane (**4d**) S52
Figure S51. ^{1}H NMR (400 MHz, benzene-d$_6$, 25°C) of phenyl(1-phenylethyl)sulfane (**4d**). S52
Figure S52. ^{13}C{^{1}H} NMR (101 MHz, benzene-d$_6$, 25°C) of phenyl(1-phenylethyl)sulfane (**4d**). S52
(1-(4-Fluorophenyl)ethyl)(phenyl)sulfane (**4e**) S53
Figure S53. ^{1}H NMR (400 MHz, Chloroform-d, 25°C) of (1-(4-fluorophenyl)ethyl)(phenyl)sulfane (**4e**). S53
Figure S54. ^{13}C{^{1}H} NMR (101 MHz, Chloroform-d, 25°C) of (1-(4-fluorophenyl)ethyl)(phenyl)sulfane (**4e**). S53
Figure S55. ^{19}F NMR (377 MHz, Chloroform-d, 25°C) of (1-(4-fluorophenyl)ethyl)(phenyl)sulfane (**4e**). S54
Benzhydryl(phenyl)sulfane (**4f**) S55
Figure S56. ^{1}H NMR (400 MHz, Chloroform-d, 25°C) of benzhydryl(phenyl)sulfane (**4f**). S55
Figure S57. ^{13}C{1H} NMR (101 MHz, Chloroform-d, 25°C) of benzhydryl(phenyl)sulfane (**4f**). S55
Phenyl(2-(*p*-tolyl)propan-2-yl)sulfane (**4g**) S56
Figure S58. ^{1}H NMR (400 MHz, Chloroform-d, 25°C) of phenyl(2-(p-tolyl)propan-2-yl)sulfane (**4g**). S56
Figure S59. ^{13}C{^{1}H} NMR (101 MHz, Chloroform-d, 25°C) of phenyl(2-(p-tolyl)propan-2-yl)sulfane (**4g**). S56
(1-(4-Chlorophenyl)ethyl)(phenyl)sulfane (**4h**) S57
Figure S61. ^{13}C{^{1}H} NMR (101 MHz, Chloroform-d, 25°C) of (1-(4-chlorophenyl)ethyl)(phenyl)sulfane (**4h**). S57
1-Methoxy-4-(4-methylpent-4-en-2-yl)benzene (**6a**) S58
Figure S62. ^{1}H NMR (400 MHz, Chloroform-d, 25°C) of 1-methoxy-4-(4-methylpent-4-en-2-yl)benzene (**6a**). S58
Figure S63. ^{13}C{^{1}H} NMR (101 MHz, Chloroform-d, 25°C) of 1-methoxy-4-(4-methylpent-4-en-2-yl)benzene (**6a**). S58
1-Methoxy-4-(pent-4-en-2-yl)benzene (**6b**) S59
Figure S64. ^{1}H NMR (400 MHz, Chloroform-d, 25°C) of 1-methoxy-4-(pent-4-en-2-yl)benzene (**6b**). S59
Figure S65. ^{13}C{1H} NMR (101 MHz, Chloroform-d, 25°C) of 1-methoxy-4-(pent-4-en-2-yl)benzene (**6b**). S59
1-Allyl-2,3-dihydro-1H-indene (**6c**) S60
Figure S66. ^{1}H NMR (400 MHz, Chloroform-d, 25°C) of 1-allyl-2,3-dihydro-1H-indene (**6c**). S60
Figure S67. ^{13}C{^{1}H} NMR (101 MHz, Chloroform-d, 25°C) of 1-allyl-2,3-dihydro-1H-indene (**6c**). S60
1-Methoxy-4-(1-(4-methoxyphenyl)ethyl)-2,5-dimethylbenzene (**6d**) S61
Figure S68. ^{1}H NMR (400 MHz, Chloroform-d, 25°C) of 1-methoxy-4-(1-(4-methoxyphenyl)ethyl)-2,5-dimethylbenzene (**6d**). S61
Figure S69. ^{13}C{^{1}H} NMR (101 MHz, Chloroform-d, 25°C) of 1-methoxy-4-(1-(4-methoxyphenyl)ethyl)-2,5-dimethylbenzene (**6d**). .. S61
1,3,5-Trimethoxy-2-(1-(4-methoxyphenyl)ethyl)benzene (**6e**) S62
Figure S70. ^{1}H NMR (400 MHz, Chloroform-d, 25°C) of 1,3,5-trimethoxy-2-(1-(4-methoxyphenyl)ethyl)benzene (**6e**). S62
Figure S71. ^{13}C{^{1}H} NMR (101 MHz, Chloroform-d, 25°C) of 1,3,5-trimethoxy-2-(1-(4-methoxyphenyl)ethyl)benzene (**6e**). S62
5-Bromo-3-(1-(4-methoxyphenyl)ethyl)-*1H*-indole (**6f**) S63
Figure S72. ^{1}H NMR (400 MHz, Chloroform-d, 25°C) of 5-bromo-3-(1-(4-methoxyphenyl)ethyl)-1H-indole (**6f**). S63
Figure S73. ^{13}C{^{1}H} NMR (101 MHz, Chloroform-d, 25°C) of 5-bromo-3-(1-(4-methoxyphenyl)ethyl)-1H-indole (**6f**). S63
3-(1-(4-Methoxyphenyl)ethyl)-1-methyl-*1H*-indole (**6g**) S64
Figure S74. ^{1}H NMR (400 MHz, Chloroform-d, 25°C) of 3-(1-(4-methoxyphenyl)ethyl)-1-methyl-1H-indole (**5b**). S64
Figure S75. ^{13}C{^{1}H} NMR (101 MHz, Chloroform-d, 25°C) of 3-(1-(4-methoxyphenyl)ethyl)-1-methyl-1H-indole (**5b**). S64
1-Methoxy-4-(1-(prop-2-yn-1-yloxy)ethyl)benzene (**7a**) S65
Figure S76. ^{1}H NMR (400 MHz, Chloroform-d, 25°C) of 1-methoxy-4-(1-(prop-2-yn-1-yloxy)ethyl)benzene (**7a**). S65
Figure S77. ^{13}C{^{1}H} NMR (101 MHz, Chloroform-d, 25°C) of 1-methoxy-4-(1-(prop-2-yn-1-yloxy)ethyl)benzene (**7a**). S65
REFERENCES S66

GENERAL INFORMATION

Hexafluoroisopropanol (HFIP) was obtained from Acros Organics and Fluorochem and used as received, without further purification. Other solvents used in the experiments were obtained from Honeywell, dried over calcium hydride (CaH_2), and purified by distillation. Alcohols, thiols, and other organic reagents were sourced from various suppliers, including Sigma-Aldrich, Ambeed, Fisher, Acros, Fluorochem, Angene, and TCI, and were used as received. Reaction progress – primarily the conversion of alcohols – was monitored by gas chromatography (GC) using a Bruker Scion 460-GC and an Agilent 5977B GC/MSD equipped with an Agilent 8860 GC system. The structures of products were determined by NMR spectroscopy, IR spectroscopy, MS (Mass Spectrometry), and HRMS (ESI-MS, Electrospray Ionization Mass Spectrometry). The ^{1}H NMR (400 or 600 MHz), and ^{13}C NMR (101 or 151 MHz) spectra were recorded on Bruker Avance III HD NanoBay spectrometer, using chloroform-d ($CDCl_3$) as the solvent. Deuterated solvent was purchased from Sigma Aldrich (Merck) ($CDCl_3$ 99.8 atom% D) and used as received. FT- IR spectra were taken on a Nicolet™ iS50 FTIR Spectrometer. High resolution mass spectra (HRMS) were obtained using Impact HD mass spectrometer (Q-TOF type instrument equipped with electrospray ion source; Bruker Daltonics, Germany). The sample solutions (DCM:MeOH) were infused into the ESI source by a syringe pump (direct inlet) at the flow rate of 3 µL/min. The instrument was operated under the following optimized settings: end plate voltage 500 V; capillary voltage 4.2 kV; nebulizer pressure 0.3 bar; dry gas (nitrogen) temperature 200 °C; dry gas flow rate 4 L/min. The spectrometer was previously calibrated with the standard tune mixture.

OPTIMIZATION OF REACTION CONDITIONS

Table S1. Optimization studies for a dehydrative thioetherification of alcohols[a]

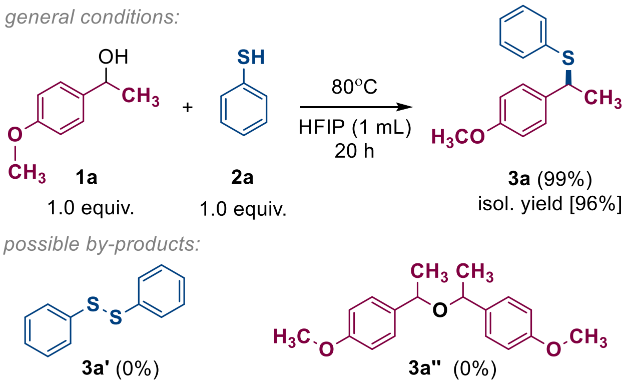

Entry	Variation of conditions	Conversion of **2a** [%]	Selectivity [**3a**:**3a'**:**3a''**][b]
1	no change	99	100 : 0 : 0
2	in 60°C	0	0 : 5 : 0
3	under Ar	99	100 : 0 : 0
4	in acetonitrile	99	86 : 14 : 0
5	in nitromethane	84	82 : 2 : 16
6	in 2-MeTHF	99	96 : 4 : 0
7	in fluorobenzene	99	95 : 5 : 0
8	in DME	99	92 : 8 : 0
9	neat	99	90 : 10 : 0

[a]General reaction conditions: **1a** (1.0 equiv., 1 mmol, 0.152 g), **2a** (1.0 equiv., 1 mmol, 0.11 g), **HFIP** (1 mL), under air atmosphere, 80°C, 20 h. [b]Ratio determined by GC-MS.

EXPERIMENTAL SECTION

Synthesis of compounds **3a**, **3b**, **3e-3i**, **3k-3o**, and **3q-3s**

To a 5 mL vial equipped with a magnetic stirring bar, 1-(4-methoxyphenyl)ethanol (**1a**) was added (0.152 g, 1 mmol). Next, thiol (1 mmol) and hexafluoroisopropanol (1 mL) were added.

Then the mixture of the reagents was stirred for 20 hours at 80°C (heat source: an aluminum heating block). After this time, HFIP was evaporated and the remaining product was dried under reduced pressure. The pure products were identified by ^{1}H NMR, ^{13}C NMR spectroscopy, and mass spectrometry. Additionally, for new compounds IR spectroscopy and HRMS were also used for identification.

Please note: Products **3e** and **3i** were additionally purified via column chromatography (hexane).

Synthesis of compounds **3c**, **3d**, **3j** and **3p**

To a 5 mL vial equipped with a magnetic stirring bar, 1-(4-methoxyphenyl)ethanol (**1a**) was added (0.152 g, 1 mmol). Next, thiol (1.02 mmol) and hexafluoroisopropanol (1 mL) were added.

Then the mixture of the reagents was stirred for 20 hours at 80°C (heat source: an aluminum heating block). After this time, HFIP was evaporated and the remaining product was dried under reduced pressure. The pure products were identified by ^{1}H NMR, ^{13}C NMR spectroscopy, and mass spectrometry. Additionally, for new compounds IR spectroscopy and HRMS were also used for identification.

Synthesis of compound **3t**

To a 5 mL vial equipped with a magnetic stirring bar, 1-(4-methoxyphenyl)ethanol (**1a**) was added (0.304 g, 1 mmol). Next, 1,10-decanedithiol (1 mmol) and hexafluoroisopropanol (1 mL) were added.

Then the mixture of the reagents was stirred for 20 hours at 80°C (heat source: an aluminum heating block). After this time, HFIP was evaporated and the remaining product was dried under reduced pressure. The pure product **3t** was identified by ^{1}H NMR, ^{13}C NMR spectroscopy, and mass spectrometry. Additionally, IR spectroscopy and HRMS were also used for identification.

Synthesis of compounds **4a-4e**

To a 5 mL vial equipped with a magnetic stirring bar, alcohol (**1**) was added (1 mmol). Next, thiophenol **2a** (0.110 g, 1 mmol) and hexafluoroisopropanol (1 mL) were added.

Then the mixture of the reagents was stirred for 20 hours at 80°C (heat source: an aluminum heating block). After this time, HFIP was evaporated and the remaining product was dried under reduced pressure. The pure products were identified by ^{1}H NMR, ^{13}C NMR spectroscopy, and mass spectrometry. Additionally, for new compounds IR spectroscopy and HRMS were also used for identification.

Synthesis of compounds **4f-4g**

To a 5 mL vial equipped with a magnetic stirring bar, alcohol (**1**) was added (1 mmol). Next, thiophenol **2a** (0.110 g, 1 mmol) and hexafluoroisopropanol (1 mL) were added.

Then the mixture of the reagents was stirred for 20 hours at 80°C (heat source: an aluminum heating block). After this time, HFIP was evaporated and the remaining crude product was dried under reduced pressure. Then, the pure product was obtained *via* column chromatography (hexane). The pure products were identified by ^{1}H NMR, ^{13}C NMR spectroscopy, and mass spectrometry.

Synthesis of compounds **4h**

To a 5 mL vial equipped with a magnetic stirring bar, 1-(4-chlorophenyl)ethanol was added (1 mmol, 0.156 g). Next, thiophenol **2a** (0.110 g, 1 mmol), hexafluoroisopropanol (1 mL), and 10 μL of trifluoroacetic acid (TFA) were added.

Then the mixture of the reagents was stirred for 30 minutes at 80°C (heat source: an aluminum heating block). After this time, TFA was neutralized by stoichiometric amount of K_2CO_3. Then, HFIP was evaporated and the remaining crude product was separated via extraction (Et_2O/water), and finally dried under reduced pressure. Then, the pure product was obtained *via* column chromatography (hexane). The product 4h was identified by ^{1}H NMR, ^{13}C NMR spectroscopy, and mass spectrometry.

Synthesis of compounds **6a-6c**

To a 5 mL vial equipped with a magnetic stirring bar, alcohol (**1**) was added (1 mmol). Next, allyltrimethylsilane (2 mmol) or 2-methylallyltrimethylsilane (2 mmol) and hexafluoroisopropanol (1 mL) were added.

Then the mixture of the reagents was stirred for 20 hours at 80°C (heat source: an aluminum heating block). After this time, HFIP was evaporated and the remaining product was separated from all low-boiling residues under reduced pressure. The pure products were identified by ^{1}H NMR, ^{13}C NMR spectroscopy, and mass spectrometry. Additionally, for new compounds IR spectroscopy and HRMS were also used for identification.

Synthesis of compounds **6d** and **6e**

To a 5 mL vial equipped with a magnetic stirring bar, 1-(4-methoxyphenyl)ethanol (**1a**) was added (0.152 g, 1 mmol). Next, an appropriate arene (1 mmol) and hexafluoroisopropanol (1 mL) were added.

Then the mixture of the reagents was stirred for 20 hours at 80°C (heat source: an aluminum heating block). After this time, HFIP was evaporated and the remaining product was dried under reduced pressure. The pure products **6d** and **6e** were identified by ^{1}H NMR, ^{13}C NMR spectroscopy, and mass spectrometry. Additionally, IR spectroscopy and HRMS were also used for identification of compound **6d**.

Synthesis of compounds **6f** and **6**g

To a 5 mL vial equipped with a magnetic stirring bar, 1-(4-methoxyphenyl)ethanol (**1a**) was added (0.152 g, 1 mmol). Next, an appropriate indole (1 mmol) and hexafluoroisopropanol (1 mL) were added.

Then the mixture of the reagents was stirred for 20 hours at 80°C (heat source: an aluminum heating block). After this time, HFIP was evaporated and the remaining product was dried under reduced pressure. The pure products **6f** and **6g** were identified by ^{1}H NMR, ^{13}C NMR spectroscopy, and mass spectrometry. Additionally, IR spectroscopy and HRMS were also used for identification of both compounds.

Synthesis of compound **7a**

To a 5 mL vial equipped with a magnetic stirring bar, 1-(4-methoxyphenyl)ethanol (**1a**) was added (0.152 g, 1 mmol). Next, propargyl alcohol (10 mmol) and hexafluoroisopropanol (1 mL) were added.

Then the mixture of the reagents was stirred for 20 hours at 80°C (heat source: an aluminum heating block). After this time, HFIP was evaporated and the remaining product was dried under reduced pressure. The pure product **7a** was identified by ^{1}H NMR, ^{1}3C NMR spectroscopy, and mass spectrometry.

Scaled-up synthesis of **3a**

To a 5 mL vial equipped with a magnetic stirring bar, 1-(4-methoxyphenyl)ethanol (**1a**) was added (0.76 g, 5 mmol). Next, thiophenol **2a** (0.55 g, 5 mmol) and hexafluoroisopropanol (5 mL) were added.

Then the mixture of the reagents was stirred for 20 hours at 80°C (heat source: an aluminum heating block). After this time, HFIP was evaporated and the remaining product was dried under reduced pressure. The pure product **3a** was obtained in 98% yield (1.2 g).

CHARACTERIZATION DATA FOR ALL PRODUCTS

(1-(4-Methoxyphenyl)ethyl)(phenyl)sulfane (**3a**)

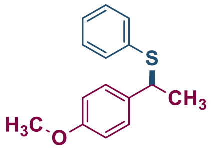

(1-(4-Methoxyphenyl)ethyl)(phenyl)sulfane was obtained in 96% yield as a pale-yellow oil (0.232 g). The title compound was known in the literature, and all spectroscopic data are in agreement.[1]

^{1}H NMR: (400 MHz, $CDCl_3$) δ 7.24 – 7.17 (m, 2H), 7.16 – 7.08 (m, 5H), 6.83 – 6.60 (m, 2H), 4.24 (q, *J* = 7.0 Hz, 1H), 3.68 (s, 3H), 1.51 (dd, *J* = 7.1, 0.9 Hz, 3H).

^{13}C{^{1}H} NMR: (101 MHz, $CDCl_3$) δ 158.7, 135.4, 135.3, 132.5, 128.8, 128.4, 127.1, 113.8, 55.3, 47.5, 22.5.

EI-MS m/z (rel. int.): 135 (100%), 109 (25), 91 (40), 65 (35).

(1-(4-Methoxyphenyl)ethyl)(*p*-tolyl)sulfane (**3b**)

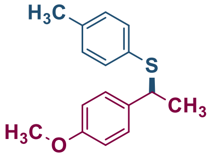

(1-(4-Methoxyphenyl)ethyl)(*p*-tolyl)sulfane was obtained in 83% yield as a white solid (0.214 g). The title compound was known in the literature, and all spectroscopic data are in agreement.[2]

^{1}H NMR: (400 MHz, $CDCl_3$) δ 7.21 – 7.06 (m, 1H), 6.99 – 6.92 (m, 4H), 6.72 (dd, *J* = 8.5, 1.7 Hz, 2H), 4.17 (qd, *J* = 7.0, 1.8 Hz, 1H), 3.69 (s, 3H), 2.21 (s, 3H), 1.50 (dt, *J* = 7.0, 1.2 Hz, 3H).

^{13}C{1H} NMR: (101 MHz, $CDCl_3$) δ 158.7, 137.4, 135.5, 133.3, 131.6, 129.6, 128.5, 113.8, 55.4, 47.9, 22.4, 21.2.

EI-MS m/z (rel. int.): 135 (100%), 123 (20), 105 (15), 91 (70).

1-(4-Isopropylphenyl)(1-(4-methoxyphenyl)ethyl)sulfane (**3c**)

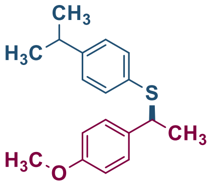

1-(4-Isopropylphenyl)(1-(4-methoxyphenyl)ethyl)sulfane was obtained in 93% yield as a pale-yellow oil (0.266 g). The title compound is a new compound.

^{1}H NMR: (400 MHz, $CDCl_3$) δ 7.25 – 7.11 (m, 4H), 7.09 – 6.98 (m, 2H), 6.80 – 6.69 (m, 2H), 4.32 – 4.14 (m, 1H), 3.81 – 3.64 (m, 3H), 3.06 – 2.70 (m, 1H), 1.73 – 1.42 (m, 3H), 1.29 – 0.98 (m, 6H).

^{13}C{1H} NMR: (101 MHz, $CDCl_3$) δ 158.8, 148.3, 135.6, 133.1, 132.1, 128.5, 127.0, 113.9, 55.4, 47.8, 33.5, 24.0, 21.9.

EI-MS m/z (rel. int.): 135 (100%), 119 (5), 91 (20), 65 (5).

HRMS (ESI-TOF) m/z: $[M + Na]^+$ Calcd for $C_{18}H_{22}OSNa$ 309.1284; Found 309.1280.

IR: (neat) vmax cm^{-1}: 2959, 2926, 2834, 1510, 1284, 1175, 826, 756.

(4-(*Tert*-butyl)phenyl)(1-(4-methoxyphenyl)ethyl)sulfane (**3d**)

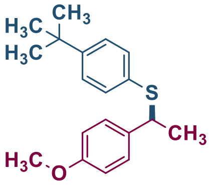

(4-(*Tert*-butyl)phenyl)(1-(4-methoxyphenyl)ethyl)sulfane was obtained in 90% yield as a pale-yellow oil (0.269 g). The title compound was known in the literature, and all spectroscopic data are in agreement.[3]

^{1}H NMR: (400 MHz, $CDCl_3$) δ 7.31 – 7.01 (m, 6H), 6.88 – 6.63 (m, 2H), 4.31 – 4.07 (m, 1H), 3.70 (d, *J* = 1.9 Hz, 3H), 1.72 – 1.42 (m, 3H), 1.24 – 1.17 (m, 9H).

^{13}C{^{1}H} NMR: (101 MHz, $CDCl_3$) δ 158.7, 150.5, 135.5, 133.3, 131.9, 128.5, 125.8, 113.8, 55.4, 47.6, 34.6, 31.4, 22.6.

EI-MS m/z (rel. int.): 300 (M^+, 5%), 151 (15), 135 (100), 91 (30).

(1-(4-Methoxyphenyl)ethyl)(naphthalen-2-yl)sulfane (**3e**)

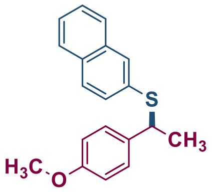

(1-(4-Methoxyphenyl)ethyl)(naphthalen-2-yl)sulfane was obtained in 76% yield as a white solid (0.223 g). The title compound was known in the literature, and all spectroscopic data are in agreement.[4]

^{1}H NMR: (400 MHz, $CDCl_3$) 7.83 – 7.76 (m, 2H), 7.76 – 7.67 (m, 2H), 7.54 – 7.43 (m, 2H), 7.40 (dd, *J* = 8.5, 1.8 Hz, 1H), 7.31 – 7.25 (m, 2H), 6.95 – 6.79 (m, 2H), 4.48 (q, *J* = 7.0 Hz, 1H), 3.79 (s, 3H), 1.67 (d, *J* = 7.0 Hz, 3H).

^{13}C{^{1}H} NMR: (101 MHz, $CDCl_3$) δ 159.1, 135.6, 134.0, 133.3, 132.7, 131.4, 130.2, 128.8, 128.6, 128.1, 127.8, 126.8, 126.4, 114.2, 55.7, 47.7, 22.9.

EI-MS m/z (rel. int.): 294 (M^+, 5%), 160 (10%), 135 (100), (20).

(4-Ethoxyphenyl)(1-(4-methoxyphenyl)ethyl)sulfane (**3f**)

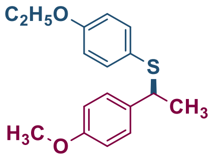

(4-Ethoxyphenyl)(1-(4-methoxyphenyl)ethyl)sulfane was obtained in 95% yield as a colorless oil (0.274 g). The title compound is a new compound.

^{1}H NMR: (400 MHz, $CDCl_3$) δ 7.26 – 6.87 (m, 3H), 6.85 – 6.55 (m, 5H), 4.31 – 4.21 (m, 1H), 3.94 – 3.74 (m, 2H), 3.69 (s, 3H), 1.53 (dd, *J* = 7.0, 2.5 Hz, 3H), 1.29 (td, *J* = 7.0, 1.8 Hz, 3H).

^{13}C{^{1}H} NMR: (101 MHz, $CDCl_3$) δ 158.7, 158.5, 136.3, 135.0, 129.2, 128.1, 124.1, 117.5, 113.6, 113.4, 63.2, 55.0, 47.0, 22.3, 14.6.

EI-MS m/z (rel. int.): 135 (100%), 119 (10), 91 (20), 65 (10).

HRMS (ESI-TOF) m/z: $[M + Na]^+$ Calcd for $C_{17}H_{20}O_2SNa$ 311.1076; Found 311.1074.

IR: (neat) vmax cm^{-1}: 2976, 2834, 1609, 1585, 1442, 1303, 1113, 855.

(3-Chloro-4-fluorophenyl)(1-(4-methoxyphenyl)ethyl)sulfane (**3g**)

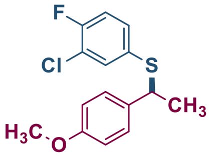

(3-Chloro-4-fluorophenyl)(1-(4-methoxyphenyl)ethyl)sulfane was obtained in 98% yield as a pale-yellow oil (0.290 g). The title compound is a new compound.

^{1}H NMR: (400 MHz, $CDCl_3$) δ 7.24 – 7.18 (m, 1H), 7.12 – 7.04 (m, 2H), 7.06 – 6.97 (m, 1H), 6.88 (t, *J* = 8.7 Hz, 1H), 6.76 – 6.65 (m, 2H), 4.15 (q, *J* = 7.0 Hz, 1H), 3.69 (s, 3H), 1.50 (d, *J* = 7.1 Hz, 3H).

^{13}C{^{1}H} NMR: (101 MHz, $CDCl_3$) δ 158.6 (d, *J* = 9.4 Hz), 156.2, 135.0, 134.3, 133.0 (d, *J* = 7.1 Hz), 131.3, 128.2, 120.7 (d, *J* = 18.1 Hz), 116.42 (d, *J* = 21.3 Hz), 113.6, 55.1, 48.0, 21.8.

^{19}F NMR: (377 MHz, $CDCl_3$) δ -116.5.

EI-MS m/z (rel. int.): 135 (100%), 126 (20), 105 (15), 91 (50).

HRMS (ESI-TOF) m/z: $[M + Na]^+$ Calcd for $C_{15}H_{14}ClFOSNa$ 319.0330; Found 319.0320.

IR: (neat) vmax cm^{-1}: 2965, 2927, 2835, 1609, 1510, 1284, 1175, 1031.

(4-Chlorophenyl)(1-(4-methoxyphenyl)ethyl)sulfane (**3h**)

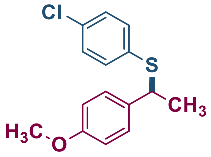

(4-Chlorophenyl)(1-(4-methoxyphenyl)ethyl)sulfane was obtained in 99% yield as a white solid (0.275 g). The title compound was known in the literature, and all spectroscopic data are in agreement.[5]

^{1}H NMR: (400 MHz, $CDCl_3$) δ 7.23 – 7.15 (m, 6H), 6.90 – 6.69 (m, 2H), 4.28 (q, *J* = 7.0 Hz, 1H), 3.79 (s, 3H), 1.60 (d, *J* = 7.0 Hz, 3H).

^{13}C{^{1}H} NMR: (101 MHz, $CDCl_3$) δ 158.8, 135.0, 134.1, 133.8, 133.4, 128.9, 128.5, 113.9, 55.4, 47.8, 22.4.

EI-MS m/z (rel. int.): 143 (20%), 135 (100), 91 (40), 65 (30).

(4-Bromophenyl)(1-(4-methoxyphenyl)ethyl)sulfane (**3i**)

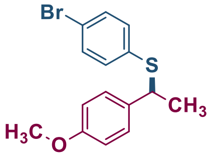

(4-Bromophenyl)(1-(4-methoxyphenyl)ethyl)sulfane was obtained in 84% yield as a pale-yellow oil (0.270 g). The title compound is a new compound.

^{1}H NMR: (400 MHz, $CDCl_3$) δ 7.37 – 7.29 (m, 2H), 7.23 – 7.15 (m, 2H), 7.15 – 7.07 (m, 2H), 6.89 – 6.76 (m, 2H), 4.29 (q, J = 7.0 Hz, 1H), 3.79 (s, 3H), 1.60 (d, J = 7.0 Hz, 3H).

^{13}C{^{1}H} NMR: (101 MHz, $CDCl_3$) δ 158.5, 134.6, 134.2, 133.8, 131.5, 128.1, 121.1, 113.6, 55.1, 47.3, 22.1.

EI-MS m/z (rel. int.): 189 (15%), 135 (100), 108 (25), 91 (50).

HRMS (ESI-TOF) m/z: $[M + Na]^+$ Calcd for $C_{15}H_{15}BrOSNa$ 344.9919; Found 344.9909.

IR: (neat) vmax cm^{-1}: 2963, 2833, 1609, 1509, 1471, 1111, 1007, 829.

(1-(4-Methoxyphenyl)ethyl)(4-(trifluoromethoxy)phenyl)sulfane (**3j**)

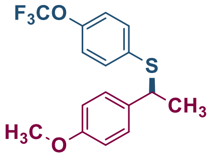

(1-(4-Methoxyphenyl)ethyl)(4-(trifluoromethoxy)phenyl)sulfane was obtained in 91% yield as a colorless oil (0.299 g). The title compound is a new compound.

^{1}H NMR: (400 MHz, $CDCl_3$) δ 7.24 – 7.14 (m, 2H), 7.13 – 7.05 (m, 2H), 7.00 – 6.88 (m, 2H), 6.77 – 6.66 (m, 2H), 4.19 (q, J = 7.0 Hz, 1H), 3.68 (s, 3H), 1.51 (d, J = 7.0 Hz, 3H).

^{13}C{^{1}H} NMR: (101 MHz, $CDCl_3$) δ 158.9, 148.5 (q, J = 1.9 Hz), 134.9, 134.1, 134.1, 129.4, 128.4, 121.2, 113.9, 55.3, 47.9, 22.4.

^{19}F NMR: (377 MHz, $CDCl_3$) δ -57.9.

EI-MS m/z (rel. int.): 192 (30%), 135 (100), 91 (30), 69 (35).

HRMS (ESI-TOF) m/z: $[M + Na]^+$ Calcd for $C_{16}H_{15}F_3O_2SNa$ 351.0637; Found 351.0638.

IR: (neat) vmax cm^{-1}: 2969, 2837, 1610, 1488, 1303, 1246, 1110, 829.

(1-(4-Methoxyphenyl)ethyl)(2-(trifluoromethyl)phenyl)sulfane (**3k**)

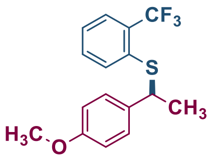

(1-(4-Methoxyphenyl)ethyl)(2-(trifluoromethyl)phenyl)sulfane was obtained in 90% yield as a colorless oil (0.281 g). The title compound is a new compound.

^{1}H NMR: (400 MHz, $CDCl_3$) δ 7.68 – 7.59 (m, 1H), 7.41 – 7.34 (m, 2H), 7.50 – 7.12 (m, 3H), 6.82 (d, J = 8.8 Hz, 2H), 4.43 (q, J = 6.9 Hz, 1H), 3.78 (s, 3H), 1.63 (d, J = 7.0 Hz, 3H).

^{13}C{^{1}H} NMR: (101 MHz, $CDCl_3$) δ 159.2, 135.0, 135.0, 132.0, 128.9, 127.2, 127.1, 127.1, 127.0, 124.2 (q, J = 273.8 Hz), 114.3, 55.7, 48.3, 22.8.

^{19}F NMR: (377 MHz, $CDCl_3$) δ -60.1.

EI-MS m/z (rel. int.): 177 (20%), 157 (20), 135 (100), 91 (20).

HR-ESI-MS (TOF) m/z: calcd.: 335.0688 (adduct [M]Na); found: 335.0685 (adduct [M]Na).

HRMS (ESI-TOF) m/z: $[M + Na]^+$ Calcd for $C_{16}H_{15}F_3OSNa$ 335.0688; Found 335.0685.

IR: (neat) vmax cm^{-1}: 2969, 2837, 1610, 1511, 1310, 1247, 1127, 1031.

5-Fluoro-2-((1-(4-methoxyphenyl)ethyl)thio)benzo[d]oxazole (**3l**)

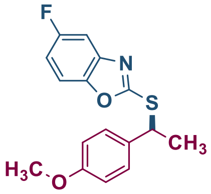

5-Fluoro-2-((1-(4-methoxyphenyl)ethyl)thio)benzo[d]oxazole was obtained in 98% yield as a colorless oil (0.297 g). The title compound is a new compound.

^{1}H NMR: (400 MHz, $CDCl_3$) δ 7.22 (dd, J = 9.0, 0.9 Hz, 2H), 7.14 (dd, J = 8.9, 4.2 Hz, 1H), 6.80 (d, J = 8.8 Hz, 2H), 6.76 (td, J = 9.1, 2.5 Hz, 1H), 6.41 – 6.23 (m, 2H), 3.70 (s, 3H), 1.76 (d, J = 7.2 Hz, 3H).

^{13}C{^{1}H} NMR: (101 MHz, $CDCl_3$) δ 181.6, 159.9 (d, J = 243.1 Hz), 159.9 (s), 143.6 (d, J = 1.8 Hz), 130.8 (d, J = 13.1 Hz), 128.9, 128.5, 114.7, 111.1 (d, J = 3.0 Hz), 110.96 (d, J = 12.6 Hz), 99.59 (d, J = 29.9 Hz), 56.1, 55.6, 15.9.

^{19}F NMR: (377 MHz, $CDCl_3$) δ -115.55 – -115.69 (m).

EI-MS m/z (rel. int.): 135 (100%), 119 (20), 91 (60), 65 (30).

HRMS (ESI-TOF) m/z: $[M + Na]^+$ Calcd for $C_{16}H_{14}FNO_2SNa$ 326.0622; Found 326.0623.

IR: (neat) vmax cm^{-1}: 2970, 2836, 1611, 1513, 1486, 1304, 1102, 1027, 962.

2-((1-(4-Methoxyphenyl)ethyl)thio)thiophene (**3m**)

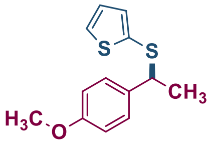

2-((1-(4-Methoxyphenyl)ethyl)thio)thiophenewas obtained in 99% yield as a colorless oil (0.248 g). The title compound is a new compound.

^{1}H NMR: (400 MHz, $CDCl_3$) δ 7.26 – 7.14 (m, 1H), 7.10 – 6.98 (m, 2H), 6.87 – 6.79 (m, 2H), 6.72 (d, J = 8.7 Hz, 2H), 4.07 (q, J = 7.1 Hz, 1H), 3.69 (s, 3H), 1.52 (d, J = 7.0 Hz, 3H).

^{13}C{^{1}H} NMR: (101 MHz, $CDCl_3$) δ 158.9, 135.3, 134.7, 133.2, 130.2, 128.6, 127.4, 113.8, 55.3, 50.5, 21.6.

EI-MS m/z (rel. int.): 135 (100%), 115 (10), 91 (40), 71 (35).

HRMS (ESI-TOF) m/z: $[M + Na]^+$ Calcd for $C_{13}H_{14}OS_2Na$ 273.0378; Found 273.0379.

IR: (neat) vmax cm^{-1}: 2962, 2833, 1609, 1509, 1441, 1303, 1244, 1030, 828.

Heptyl(1-(4-methoxyphenyl)ethyl)sulfane (**3n**)

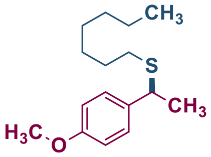

Heptyl(1-(4-methoxyphenyl)ethyl)sulfane was obtained in 94% yield as a colorless oil (0.250 g). The title compound is a new compound.

^{1}H NMR: (400 MHz, $CDCl_3$) δ 7.42 – 7.18 (m, 2H), 6.89 – 6.82 (m, 2H), 3.93 (q, *J* = 7.0 Hz, 1H), 3.80 (s, 3H), 2.38 – 2.20 (m, 2H), 1.55 (d, *J* = 7.1 Hz, 3H), 1.54 – 1.41 (m, 2H), 1.37 – 1.12 (m, 10H), 0.88 (td, *J* = 7.0, 1.4 Hz, 3H).

^{13}C{^{1}H} NMR: (101 MHz, $CDCl_3$) δ 158.9, 136.7, 128.6, 114.1, 55.6, 43.8, 32.1, 31.7, 29.8, 29.3, 29.3, 23.2, 23.0, 14.5.

EI-MS m/z (rel. int.): 266 (M^+, 5%), 135 (100), 119 (10), 91 (25).

HRMS (ESI-TOF) m/z: $[M + Na]^+$ Calcd for $C_{16}H_{26}OSNa$ 289.1597; Found 289.1602.

IR: (neat) vmax cm^{-1}: 2954, 2854, 1610, 1510, 1280, 1034, 830, 543.

(1-(4-Methoxyphenyl)ethyl)(octyl)sulfane (**3o**)

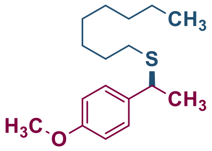

(1-(4-Methoxyphenyl)ethyl)(octyl)sulfane was obtained in 99% yield as a colorless oil (0.277 g). The title compound was known in the literature, and all spectroscopic data are in agreement.[4]

^{1}H NMR: (400 MHz, $CDCl_3$) δ 7.18 (d, *J* = 8.7 Hz, 2H), 6.77 (d, *J* = 8.7 Hz, 2H), 3.84 (q, *J* = 7.0 Hz, 1H), 3.72 (s, 3H), 2.38 – 2.09 (m, 2H), 1.46 (d, *J* = 7.0 Hz, 3H), 1.44 – 1.33 (m, 2H), 1.26 – 1.08 (m, 10H), 0.80 (t, *J* = 6.9 Hz, 3H).

^{13}C{^{1}H} NMR: (101 MHz, $CDCl_3$) δ 158.6, 136.4, 128.4, 113.9, 55.4, 43.5, 31.9, 31.4, 29.5, 29.3, 29.3, 29.1, 22.9, 22.8, 14.2.

EI-MS m/z (rel. int.): 135 (100%), 129 (5), 91 (20), 65 (10).

Cyclohexyl(1-(4-methoxyphenyl)ethyl)sulfane (**3p**)

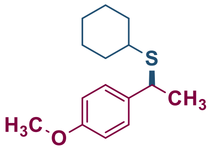

Cyclohexyl(1-(4-methoxyphenyl)ethyl)sulfane was obtained in 96% yield as a colorless oil (0.240 g). The title compound was known in the literature, and all spectroscopic data are in agreement.[4]

^{1}H NMR: (400 MHz, $CDCl_3$) δ 7.27 (d, *J* = 8.7 Hz, 2H), 6.84 (d, *J* = 8.7 Hz, 2H), 4.02 (q, *J* = 7.0 Hz, 1H), 3.80 (s, 3H), 2.45 – 2.32 (m, 1H), 2.03 – 1.89 (m, 1H), 1.79 – 1.61 (m, 4H), 1.52 (d, *J* = 7.0 Hz, 3H), 1.39 – 1.15 (m, 5H).

^{13}C{^{1}H} NMR: (101 MHz, $CDCl_3$) δ 158.9, 137.2, 128.5, 114.2, 55.7, 43.2, 42.3, 34.3, 33.8, 26.4, 26.3, 26.3, 23.7.

EI-MS m/z (rel. int.): 250 (M^+, 10%), 135 (100), 119 (10), 91 (20).

3-((1-(4-Methoxyphenyl)ethyl)thio)propanoic acid (**3q**)

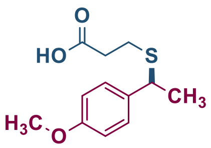

3-((1-(4-Methoxyphenyl)ethyl)thio)propanoic acid was obtained in 93% yield as a colorless oil (0.223 g). The title compound was known in the literature, and all spectroscopic data are in agreement.[3]

^{1}H NMR: (400 MHz, $CDCl_3$) δ 7.23 – 7.13 (m, 2H), 6.85 – 6.71 (m, 2H), 3.88 (q, *J* = 7.0 Hz, 1H), 3.72 (s, 3H), 2.55 – 2.45 (m, 2H), 2.44 – 2.37 (m, 2H), 1.92 (s, 1H), 1.46 (d, *J* = 7.0 Hz, 3H).

^{13}C{^{1}H} NMR: (101 MHz, $CDCl_3$) δ 178.3, 158.7, 135.7, 128.4, 114.1, 55.4, 43.8, 34.5, 25.9, 22.7.

EI-MS m/z (rel. int.): 135 (100%), 105 (10), 91 (20), 65 (15).

Methyl 2-((1-(4-methoxyphenyl)ethyl)thio)acetate (**3r**)

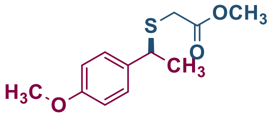

Methyl 2-((1-(4-methoxyphenyl)ethyl)thio)acetate was obtained in 98% yield as a colorless oil (0.235 g). The title compound was known in the literature, and all spectroscopic data are in agreement.[6]

^{1}H NMR: (400 MHz, $CDCl_3$) δ 7.34 – 7.06 (m, 2H), 6.96 – 6.67 (m, 2H), 4.04 (q, *J* = 7.1 Hz, 1H), 3.72 (s, 3H), 3.60 (s, 3H), 3.17 – 2.80 (m, 2H), 1.48 (d, *J* = 7.1 Hz, 3H).

^{13}C{^{1}H} NMR: (101 MHz, $CDCl_3$) δ 171.1, 158.9, 134.7, 128.6, 113.9, 55.3, 52.3, 43.8, 32.8, 22.1.

EI-MS m/z (rel. int.): 240 (M^+, 3%), 135 (100), 119 (20), 91 (50).

(4-Chlorobenzyl)(1-(4-methoxyphenyl)ethyl)sulfane (**3s**)

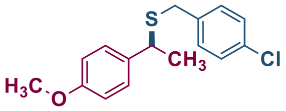

(4-Chlorobenzyl)(1-(4-methoxyphenyl)ethyl)sulfane obtained in 99% yield as a colorless oil (0.289 g). The title compound is a new compound.

^{1}H NMR: (400 MHz, $CDCl_3$) δ 7.30 – 7.21 (m, 4H), 7.19 – 7.09 (m, 2H), 6.97 – 6.81 (m, 2H), 3.82 (s, 3H), 3.77 (q, *J* = 7.1 Hz, 1H), 3.53 – 3.32 (m, 2H), 1.51 (d, *J* = 7.1 Hz, 3H).

^{13}C{^{1}H} NMR: (101 MHz, $CDCl_3$) δ 158.8, 137.2, 135.7, 132.6, 130.3, 128.6, 128.6, 114.0, 55.4, 43.2, 35.2, 22.8.

EI-MS m/z (rel. int.): 292 (M^+, 5%), 135 (100), 125 (25), 91 (20).

HRMS (ESI-TOF) m/z: $[M + Na]^+$ Calcd for $C_{16}H_{17}ClOSNa$ 315.0581; Found 315.0580.

IR: (neat) vmax cm^{-1}: 2962, 2834, 1609, 1509, 1441, 1244, 1111, 1031, 829.

1-(1-(4-Methoxyphenyl)ethyl)thio)-10-(1-(4-methoxyphenyl)ethyl)thio)decane (**3t**)

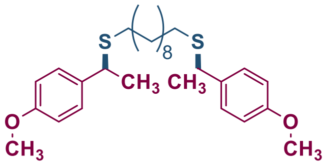

1-(1-(4-Methoxyphenyl)ethyl)thio)-10-(1-(4-methoxyphenyl)ethyl)thio)decane obtained in 99% yield as a colorless oil (0.469 g). The title compound is a new compound.

^{1}H NMR: (400 MHz, $CDCl_3$) δ 7.28 – 7.06 (m, 4H), 6.85 – 6.67 (m, 4H), 3.84 (q, J = 7.0 Hz, 2H), 3.71 (s, 6H), 2.27 – 2.16 (m, 2H), 1.46 (d, J = 7.1 Hz, 6H), 1.41 – 1.06 (m, 18H).

^{13}C{^{1}H} NMR: (101 MHz, $CDCl_3$) δ 158.9, 136.7, 128.6, 114.2, 55.7, 43.8, 31.7, 29.8, 29.8, 29.6, 29.3, 23.2.

EI-MS m/z (rel. int.): 135 (100%), 119 (10), 91 (20), 65 (5).

HR-ESI-MS (TOF) m/z: calcd.: 497.2518 (adduct [M]Na); found: 497.2515 (adduct [M]Na).

HRMS (ESI-TOF) m/z: $[M + Na]^+$ Calcd for $C_{28}H_{42}O_2S_2Na$ 497.2518; Found 497.2515.

IR: (neat) vmax cm^{-1}: 2923, 2851, 1610, 1509, 1280, 1174, 1032, 829.

Phenyl(1,2,3,4-tetrahydronaphthalen-1-yl)sulfane (**4a**)

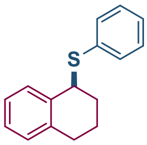

Phenyl(1,2,3,4-tetrahydronaphthalen-1-yl)sulfane obtained in 96% yield as a colorless oil (0.230 g). The title compound was known in the literature, and all spectroscopic data are in agreement.[1]

^{1}H NMR: (400 MHz, $CDCl_3$) δ 7.55 – 7.42 (m, 3H), 7.39 – 7.24 (m, 3H), 7.24 – 7.05 (m, 3H), 4.70 – 4.23 (m, 1H), 3.07 – 2.49 (m, 2H), 2.43 – 2.15 (m, 1H), 2.15 – 1.94 (m, 2H), 1.87 – 1.54 (m, 1H).

^{13}C{^{1}H} NMR: (101 MHz, $CDCl_3$) δ 137.4, 135.9, 135.3, 131.7, 130.3, 129.1, 128.7, 126.9, 126.8, 125.5, 47.4, 28.9, 28.3, 18.4.

EI-MS m/z (rel. int.): 240 (M^+, 5%), 131 (100), 115 (30), 109 (40).

(Cyclopropyl(phenyl)methyl)(phenyl)sulfane (**4b**)

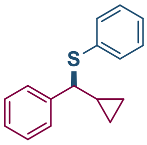

(Cyclopropyl(phenyl)methyl)(phenyl)sulfane obtained in 98% yield as a colorless oil (0.235 g). The title compound was known in the literature, and all spectroscopic data are in agreement.[7]

^{1}H NMR: (400 MHz, $CDCl_3$) δ 7.30 – 7.21 (m, 6H), 7.21 – 7.09 (m, 4H), 3.60 – 3.41 (m, 1H), 1.44 – 1.19 (m, 1H), 0.78 – 0.44 (m, 2H), 0.39 – 0.15 (m, 2H).

^{13}C{^{1}H} NMR: (101 MHz, $CDCl_3$) δ 142.3, 135.0, 133.1, 129.4, 128.6, 128.3, 127.9, 127.6, 127.2, 127.1, 59.0, 17.0, 6.6, 4.5.

EI-MS m/z (rel. int.): 207 (50%), 135 (100), 73 (60), 57 (20).

HRMS (ESI-TOF) m/z: $[M + Na]^+$ Calcd for $C_{16}H_{16}SNa$ 263.0865; Found 263.0864.

IR: (neat) vmax cm^{-1}: 3059, 3002, 1582, 1480, 1216, 1091, 1024, 840.

(2,3-Dihydro-1H-inden-1-yl)(phenyl)sulfane (**4c**)

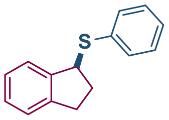

(2,3-Dihydro-1H-inden-1-yl)(phenyl)sulfane obtained in 99% yield as a colorless oil (0.224 g). The title compound was known in the literature, and all spectroscopic data are in agreement.[8]

^{1}H NMR: (400 MHz, $CDCl_3$) δ 7.35 – 7.27 (m, 2H), 7.23 – 6.99 (m, 7H), 4.73 – 4.61 (m, 1H), 3.04 – 2.85 (m, 1H), 2.83 – 2.66 (m, 1H), 2.51 – 2.36 (m, 1H), 2.24 – 2.04 (m, 1H).

^{13}C{^{1}H} NMR: (101 MHz, $CDCl_3$) δ 144.2, 143.3, 136.5, 131.8, 129.3, 128.2, 127.1, 126.9, 125.4, 125.1, 52.3, 34.1, 31.2.

EI-MS m/z (rel. int.): 117 (100%), 109 (15), 91 (10), 65 (10).

Phenyl(1-phenylethyl)sulfane (**4d**)

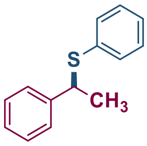

Phenyl(1-phenylethyl)sulfane obtained in 90% yield as a colorless oil (0.193 g). The title compound was known in the literature, and all spectroscopic data are in agreement.[7]

^{1}H NMR: (600 MHz, C6D$_6$) δ 7.29 – 7.23 (m, 2H), 7.21 – 7.17 (m, 2H), 7.09 – 7.04 (m, 2H), 7.01 – 6.97 (m, 1H), 6.96 – 6.86 (m, 3H), 4.14 (q, J = 7.0 Hz, 1H), 1.46 (d, J = 7.0 Hz, 3H).

^{13}C{^{1}H} NMR: (151 MHz, $CDCl_3$) δ 143.8, 136.0, 132.7, 128.9, 128.6, 127.6, 127.3, 127.2, 48.2, 22.6.

EI-MS m/z (rel. int.): 214 (M^+, 15%), 109 (45), 105 (100), 77 (40).

(1-(4-Fluorophenyl)ethyl)(phenyl)sulfane (**4e**)

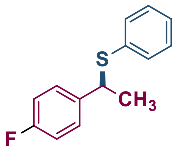

(1-(4-Fluorophenyl)ethyl)(phenyl)sulfane obtained in 96% yield as a colorless oil (0.223 g). The title compound was known in the literature, and all spectroscopic data are in agreement.[7]

^{1}H NMR: (400 MHz, $CDCl_3$) δ 7.26 – 7.08 (m, 6H), 6.90 – 6.79 (m, 2H), 4.22 (qd, J = 7.0, 1.8 Hz, 1H), 1.51 (dd, J = 7.1, 1.2 Hz, 3H).

^{13}C{^{1}H} NMR: (101 MHz, $CDCl_3$) δ 161.9 (d, J = 245.2 Hz), 139.11 (d, J = 3.2 Hz), 134.9, 132.8, 128.9 (d, J = 8.0 Hz), 128.8, 127.4, 115.26 (d, J = 21.3 Hz), 47.4, 22.5.

^{19}F NMR: (377 MHz, $CDCl_3$) δ -115.4.

EI-MS m/z (rel. int.): 232 (M^+, 10%), 123 (100), 109 (40), 96 (20).

Benzhydryl(phenyl)sulfane (**4f**)

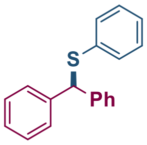

Benzhydryl(phenyl)sulfane obtained in 65% yield as a colorless oil (0.179 g). The title compound was known in the literature, and all spectroscopic data are in agreement.[7]

^{1}H NMR: (400 MHz, $CDCl_3$) δ 7.39 – 7.31 (m, 4H), 7.26 – 7.17 (m, 4H), 7.17 – 7.13 (m, 4H), 7.12 – 7.04 (m, 3H), 5.47 (s, 1H).

^{13}C{^{1}H} NMR: (101 MHz, $CDCl_3$) δ 141.1, 136.3, 130.7, 128.9, 128.7, 128.5, 127.4, 126.7, 57.6.

EI-MS m/z (rel. int.): 276 (M^+, 15%), 167 (100), 152 (25), 108 (10).

Phenyl(2-(*p*-tolyl)propan-2-yl)sulfane (**4g**)

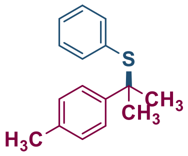

Phenyl(2-(*p*-tolyl)propan-2-yl)sulfane obtained in 57% yield as a colorless oil (0.138 g). The title compound was known in the literature, and all spectroscopic data are in agreement.[1]

^{1}H NMR: (400 MHz, $CDCl_3$) δ 7.26 – 7.21 (m, 2H), 7.16 (d, J = 4.2 Hz, 1H), 7.12 – 7.07 (m, 3H), 7.04 – 6.96 (m, 2H), 2.24 (s, 3H), 1.57 (s, 6H).

^{13}C{^{1}H} NMR: (101 MHz, $CDCl_3$) δ 143.8, 136.9, 136.5, 133.5, 129.0, 128.9, 128.7, 126.9, 51.2, 30.3, 21.4.

EI-MS m/z (rel. int.): 133 (100%), 105 (50), 91 (40), 65 (30).

(1-(4-Chlorophenyl)ethyl)(phenyl)sulfane (**4h**)

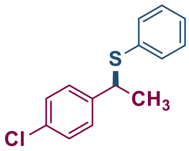

(1-(4-Chlorophenyl)ethyl)(phenyl)sulfane obtained in 97% yield as a colorless oil (0.240 g). The title compound was known in the literature, and all spectroscopic data are in agreement.[1]

^{1}H NMR: (400 MHz, $CDCl_3$) δ 7.50 – 6.53 (m, 8H), 4.21 (q, J = 7.1 Hz, 1H), 1.51 (d, J = 7.1 Hz, 3H).

^{13}C{^{1}H} NMR: (101 MHz, $CDCl_3$) δ 142.0, 134.7, 132.9, 132.8, 128.9, 128.7, 128.6, 127.5, 47.5, 22.3.

EI-MS m/z (rel. int.): 248 (5%), 139 (100), 103 (70), 77 (35).

1-Methoxy-4-(4-methylpent-4-en-2-yl)benzene (**6a**)

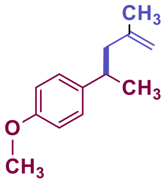

1-Methoxy-4-(4-methylpent-4-en-2-yl)benzene obtained in 96% yield as a colorless oil (0.182 g). The title compound was known in the literature, and all spectroscopic data are in agreement.[9]

^{1}H NMR: (400 MHz, $CDCl_3$) δ 7.25 – 7.11 (m, 2H), 6.93 – 6.81 (m, 2H), 4.84 – 4.59 (m, 2H), 3.81 (s, 3H), 3.02 – 2.80 (m, 1H), 2.42 – 2.16 (m, 2H), 1.72 (s, 3H), 1.23 (d, J = 6.9 Hz, 3H).

^{13}C{^{1}H} NMR: (101 MHz, $CDCl_3$) δ 158.1, 144.6, 140.0, 128.2, 114.1, 112.4, 55.6, 47.5, 37.4, 22.7, 22.3.

EI-MS m/z (rel. int.): 135 (100%), 91 (40), 77 (20), 55 (30).

1-Methoxy-4-(pent-4-en-2-yl)benzene (**6b**)

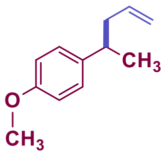

1-Methoxy-4-(pent-4-en-2-yl)benzene obtained in 90% yield as a colorless oil (0.158 g). The title compound was known in the literature, and all spectroscopic data are in agreement.[10]

^{1}H NMR: (400 MHz, $CDCl_3$) δ 7.15 (d, J = 8.8 Hz, 2H), 6.88 (d, J = 8.7 Hz, 2H), 5.86 – 5.64 (m, 1H), 5.15 – 4.89 (m, 2H), 3.82 (s, 3H), 2.79 (q, J = 7.1 Hz, 1H), 2.44 – 2.20 (m, 2H), 1.26 (d, J = 7.0 Hz, 3H).

^{13}C{^{1}H} NMR: (101 MHz, $CDCl_3$) δ 158.2, 139.6, 137.7, 128.3, 116.2, 114.1, 55.6, 43.3, 39.4, 22.1.

EI-MS m/z (rel. int.): 176 ($[M]^+$, 5%) 161 (5), 135 (100), 91 (25).

1-Allyl-2,3-dihydro-1H-indene (**6c**)

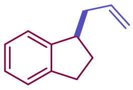

1-Allyl-2,3-dihydro-1H-indene obtained in 98% yield as a colorless oil (0.155 g). The title compound was known in the literature, and all spectroscopic data are in agreement.[11]

^{1}H NMR: (400 MHz, $CDCl_3$) δ 7.64 – 7.25 (m, 2H), 7.24 – 7.04 (m, 2H), 6.17 – 5.74 (m, 1H), 5.33 – 4.98 (m, 2H), 3.62 – 3.23 (m, 1H), 3.11 – 2.81 (m, 2H), 2.74 – 2.53 (m, 1H), 2.44 – 2.24 (m, 2H), 1.88 – 1.64 (m, 1H).

^{13}C{^{1}H} NMR: (101 MHz, $CDCl_3$) δ 147.3, 144.6, 137.6, 126.8, 126.5, 124.9, 124.1, 116.4, 44.8, 39.8, 32.0, 31.7.

EI-MS m/z (rel. int.): 128 (10%), 117 (100), 91 (20), 63 (5).

1-Methoxy-4-(1-(4-methoxyphenyl)ethyl)-2,5-dimethylbenzene (**6d**)

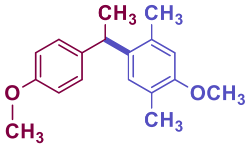

1-Methoxy-4-(1-(4-methoxyphenyl)ethyl)-2,5-dimethylbenzene obtained in 95% yield as a colorless oil (0.257 g). The title compound is a new compound.

^{1}H NMR: (400 MHz, $CDCl_3$) δ 7.16 – 7.09 (m, 2H), 7.04 (s, 1H), 6.90 – 6.80 (m, 2H), 6.65 (s, 1H), 4.24 (q, *J* = 7.2 Hz, 1H), 3.83 (s, 3H), 3.80 (s, 3H), 2.26 (s, 3H), 2.24 (s, 3H), 1.66 – 1.52 (m, 3H).

^{13}C{^{1}H} NMR: (101 MHz, $CDCl_3$) δ 157.5, 155.6, 138.9, 135.8, 134.1, 128.9, 128.4, 123.5, 113.6, 112.2, 55.2, 55.1, 39.3, 22.3, 19.7, 15.9.

EI-MS m/z (rel. int.): 270 (M^+, 30%), 255 (100), 165 (10), 90 (15).

HRMS (ESI-TOF) m/z: $[M + Na]^+$ Calcd for $C_{18}H_{22}ONa$ 293.1512; Found 293.1509.

IR: (neat) vmax cm^{-1}: 2961, 2833, 1610, 1507, 1287, 1175, 1006, 830.

1,3,5-Trimethoxy-2-(1-(4-methoxyphenyl)ethyl)benzene (**6e**)

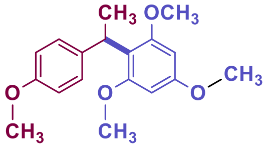

1,3,5-Trimethoxy-2-(1-(4-methoxyphenyl)ethyl)benzene obtained in 99% yield as a colorless oil (0.299 g). The title compound was known in the literature, and all spectroscopic data are in agreement.[3]

^{1}H NMR: (400 MHz, $CDCl_3$) δ 7.17 – 7.04 (m, 2H), 6.74 – 6.61 (m, 2H), 6.04 (s, 2H), 4.61 (q, *J* = 7.0, 5.1 Hz, 1H), 3.69 (d, *J* = 10.0 Hz, 6H), 3.69 (s, 3H), 3.61 (s, 6H), 1.60 – 1.43 (m, 3H).

^{13}C{^{1}H} NMR: (101 MHz, $CDCl_3$) δ 161.7, 159.4, 159.1, 157.1, 139.0, 128.3, 116.2, 113.1, 93.1, 91.6, 55.9, 55.4, 55.4, 55.3, 32.4, 18.2.

EI-MS m/z (rel. int.): 302 ($[M]^+$, 50%), 287 (80), 139 (15), 121 (100).

5-Bromo-3-(1-(4-methoxyphenyl)ethyl)-*1H*-indole (**6f**)

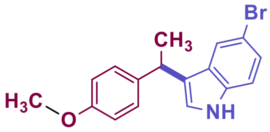

5-Bromo-3-(1-(4-methoxyphenyl)ethyl)-1H-indole obtained in 95% yield as a colorless oil (0.313 g). The title compound is a new compound.

^{1}H NMR: (400 MHz, $CDCl_3$) δ 7.88 (s, 1H), 7.46 – 7.31 (m, 1H), 7.20 – 7.00 (m, 4H), 6.87 (dd, *J* = 2.5, 1.1 Hz, 1H), 6.74 (d, *J* = 2.1 Hz, 1H), 6.72 (d, *J* = 2.1 Hz, 1H), 4.16 (q, *J* = 7.2 Hz, 1H), 3.68 (s, 3H), 1.87 (s, 1H), 1.55 (d, *J* = 7.1 Hz, 3H).

^{13}C{^{1}H} NMR: (101 MHz, $CDCl_3$) δ 157.9, 138.6, 135.4, 128.7, 128.3, 124.9, 122.4, 122.3, 121.6, 113.9, 112.6, 112.6, 55.4, 36.0, 22.6.

EI-MS m/z (rel. int.): 330 ($[M]^+$, 20%), 314 (100), 191 (35), 115 (20).

HRMS (ESI-TOF) m/z: $[M + Na]^+$ Calcd for $C_{17}H_{16}BrNONa$ 352.0308; Found 352.0304.

IR: (neat) vmax cm^{-1}: 3421, 2962, 2834, 2359, 1508, 1239, 1222, 1051, 865.

3-(1-(4-Methoxyphenyl)ethyl)-1-methyl-*1H*-indole (**6g**)

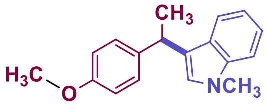

3-(1-(4-Methoxyphenyl)ethyl)-1-methyl-*1H*-indole obtained in 96% yield as a colorless oil (0.254 g). The title compound is a new compound.

^{1}H NMR: (400 MHz, $CDCl_3$) δ 7.32 – 7.26 (m, 1H), 7.20 – 7.03 (m, 4H), 6.95 – 6.85 (m, 1H), 6.75 – 6.70 (m, 3H), 4.24 (q, *J* = 7.2 Hz, 1H), 3.67 (s, 3H), 3.64 (s, 3H), 1.58 (dd, *J* = 7.0, 1.2 Hz, 3H).

^{13}C{^{1}H} NMR: (101 MHz, $CDCl_3$) δ 158.2, 139.6, 137.8, 128.7, 127.7, 127.1, 126.3, 121.9, 120.8, 120.2, 119.0, 114.3, 114.1, 109.5, 55.6, 36.5, 33.1, 23.1.

EI-MS m/z (rel. int.): 265 (20%), 250 (100), 206 (20), 115 (5).

HRMS (ESI-TOF) m/z: $[M + Na]^+$ Calcd for $C_{18}H_{19}NONa$ 288.1359; Found 288.1364.

IR: (neat) vmax cm^{-1}: 2960, 2833, 1675, 1583, 1423, 1368, 1327, 1237.

1-Methoxy-4-(1-(prop-2-yn-1-yloxy)ethyl)benzene (**7a**)

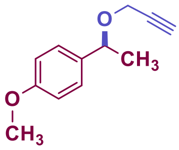

1-Methoxy-4-(1-(prop-2-yn-1-yloxy)ethyl)benzene obtained in 85% yield as a colorless oil (0.161 g). The title compound was known in the literature, and all spectroscopic data are in agreement.[12]

^{1}H NMR: (400 MHz, $CDCl_3$) δ 7.18 (d, *J* = 8.6 Hz, 2H), 6.81 (d, *J* = 8.7 Hz, 2H), 4.53 (q, *J* = 6.5 Hz, 1H), 4.00 – 3.74 (m, 2H), 3.73 (s, 3H), 2.32 (s, 1H), 1.39 (d, *J* = 6.5 Hz, 3H).

^{13}C{^{1}H} NMR: (101 MHz, $CDCl_3$) δ 159.4, 134.4, 127.9, 114.0, 80.2, 76.3, 74.0, 55.4, 55.3, 23.7.

EI-MS m/z (rel. int.): 175 ($[M-CH_3]^+$, 50%), 135 (100), 91 (80), 65 (40).

SPECTRA FOR ALL PRODUCTS

(1-(4-Methoxyphenyl)ethyl)(phenyl)sulfane (**3a**)

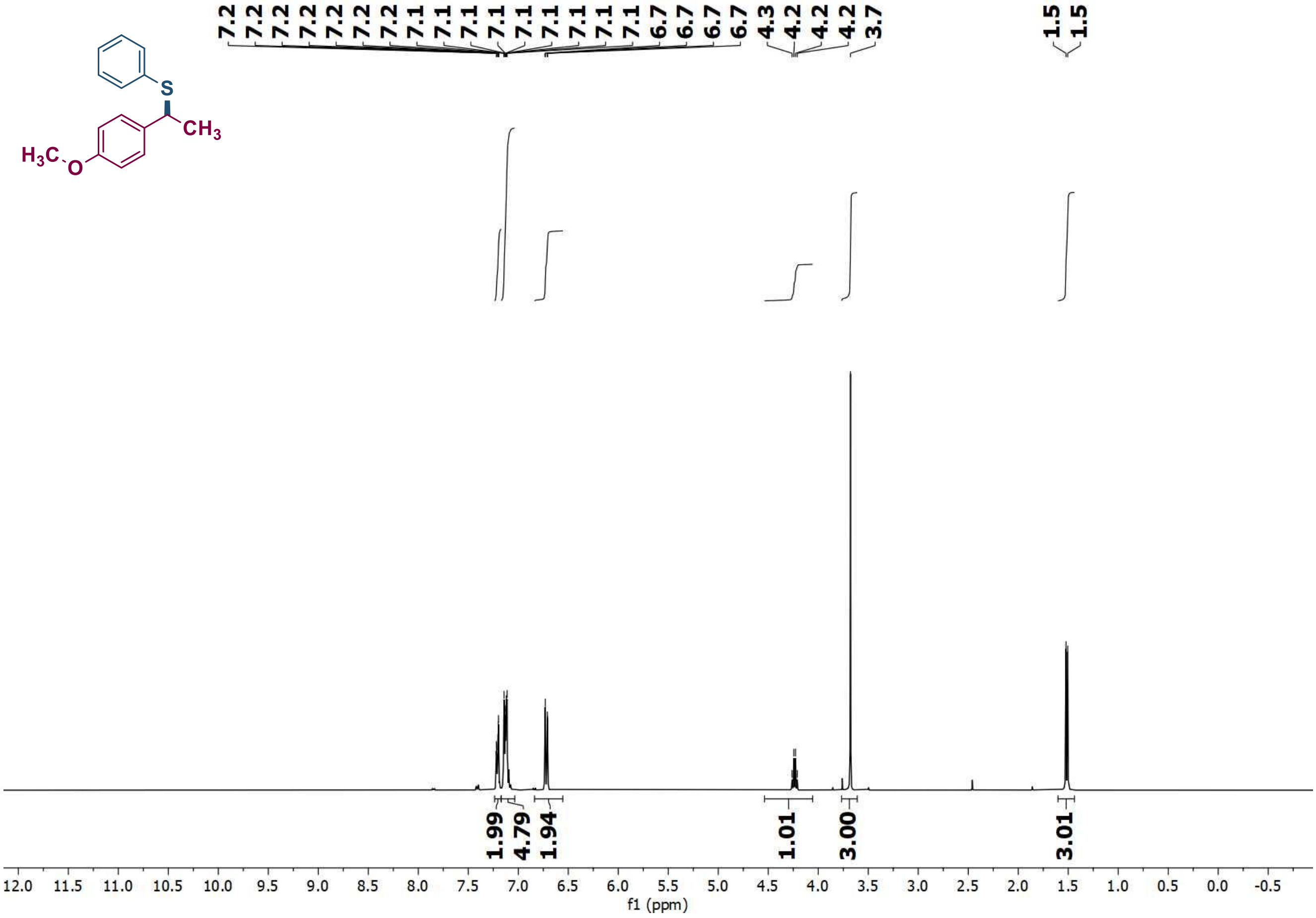

Figure S1. ^{1}H NMR (400 MHz, Chloroform-d, 25°C) of (1-(4-methoxyphenyl)ethyl)(phenyl)sulfane (**3a**).

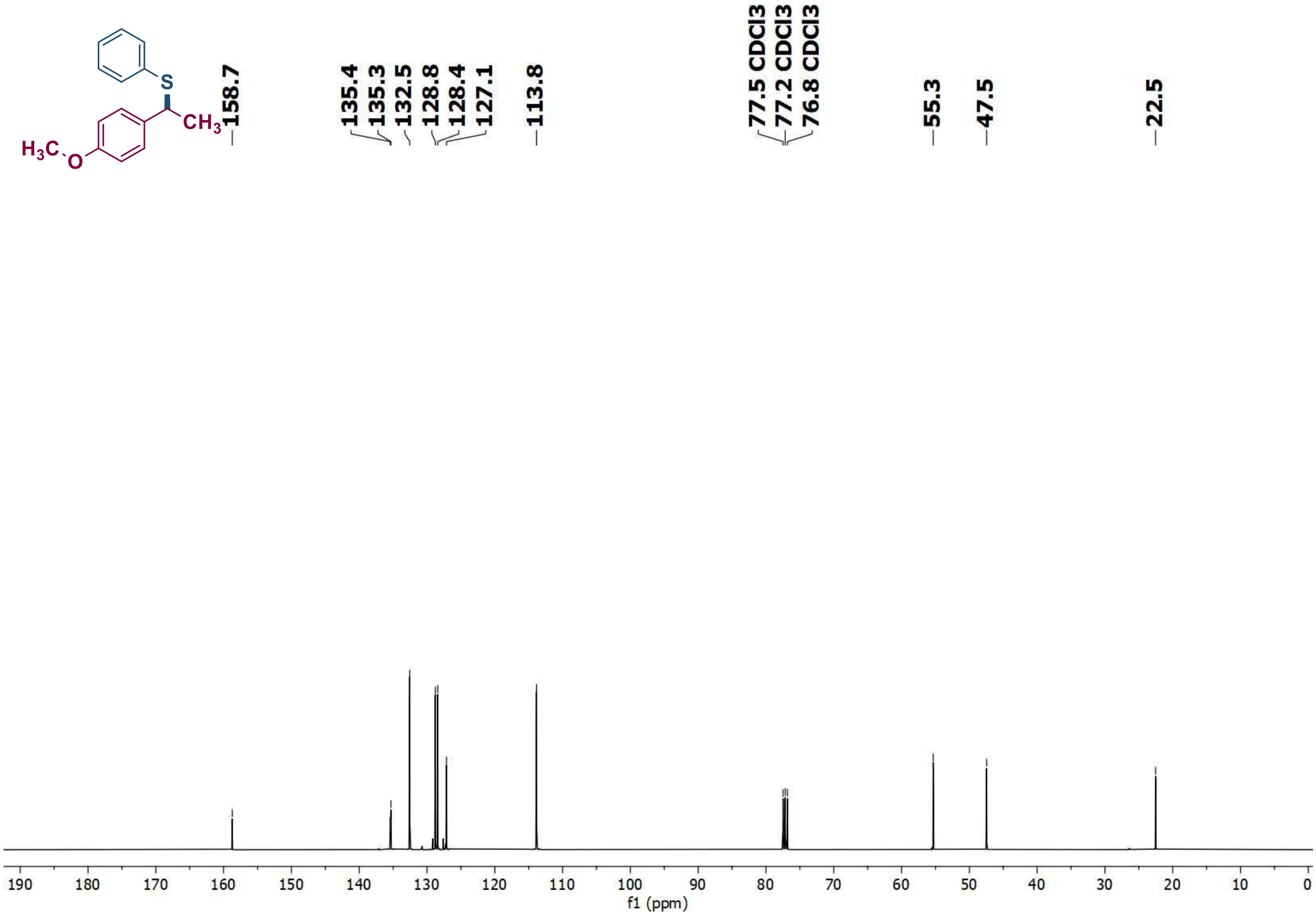

Figure S2. $^{13}C\{^{1}H\}$ NMR (101 MHz, Chloroform-d, 25°C) of (1-(4-methoxyphenyl)ethyl)(phenyl) (**3a**).

(1-(4-Methoxyphenyl)ethyl)(*p*-tolyl)sulfane (**3b**)

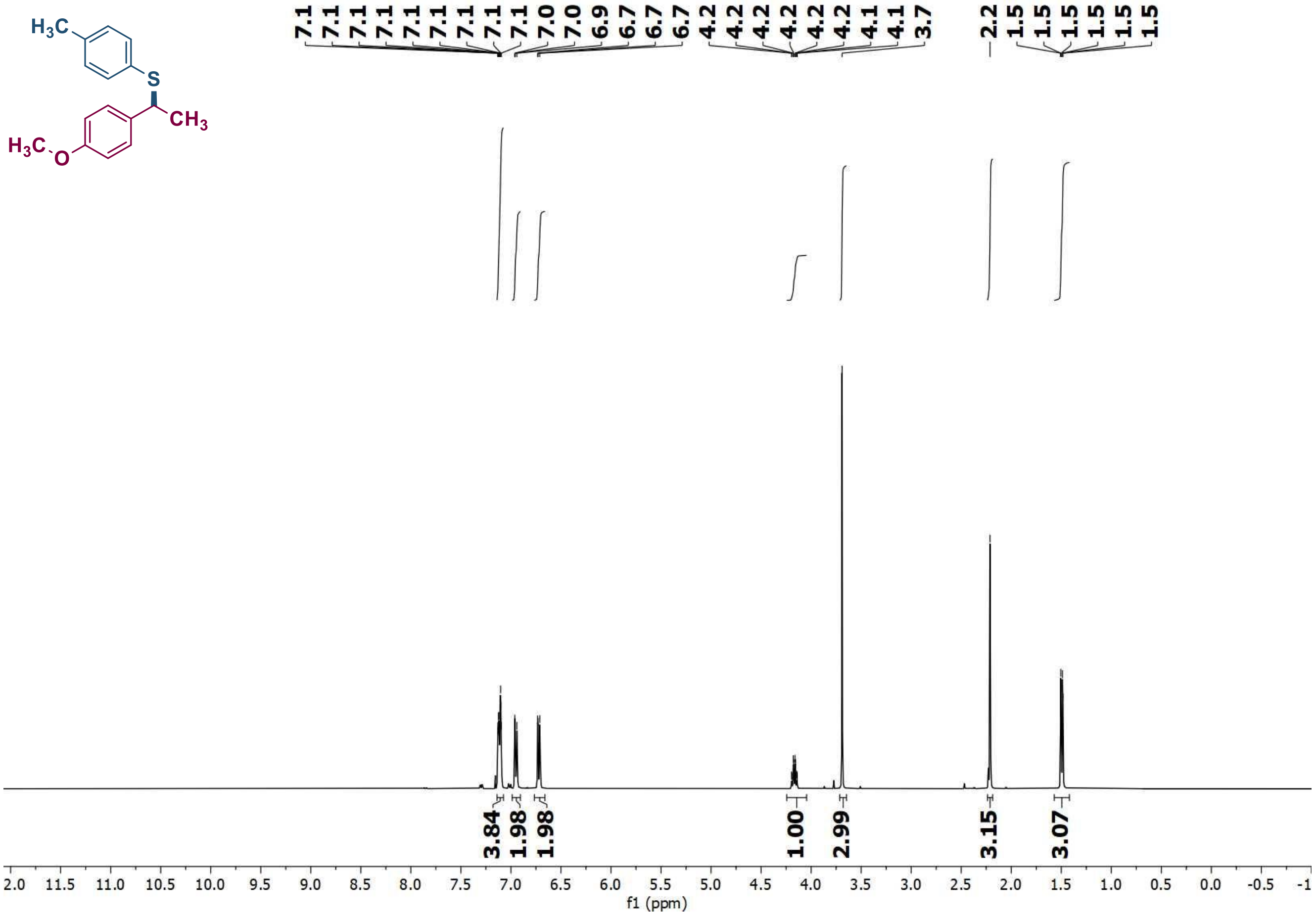

Figure S3. ^{1}H NMR (400 MHz, Chloroform-d, 25°C) of (1-(4-methoxyphenyl)ethyl)(*p*-tolyl)sulfane (**3b**).

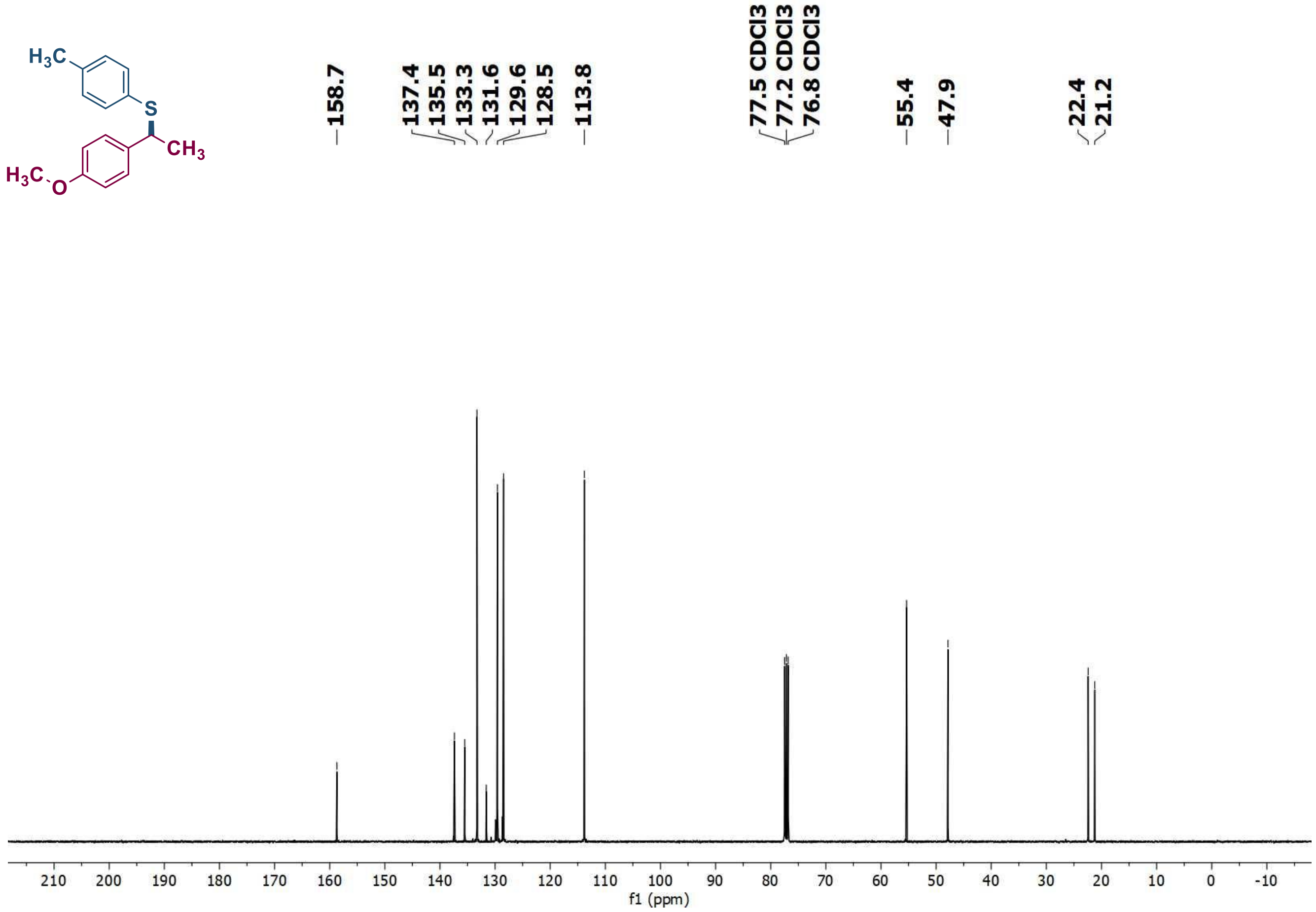

Figure S4. ^{13}C{^{1}H} NMR (101 MHz, Chloroform-d, 25°C) of (1-(4-methoxyphenyl)ethyl)(*p*-tolyl)sulfane (**3b**).

1-(4-Isopropylphenyl)(1-(4-methoxyphenyl)ethyl)sulfane (**3c**)

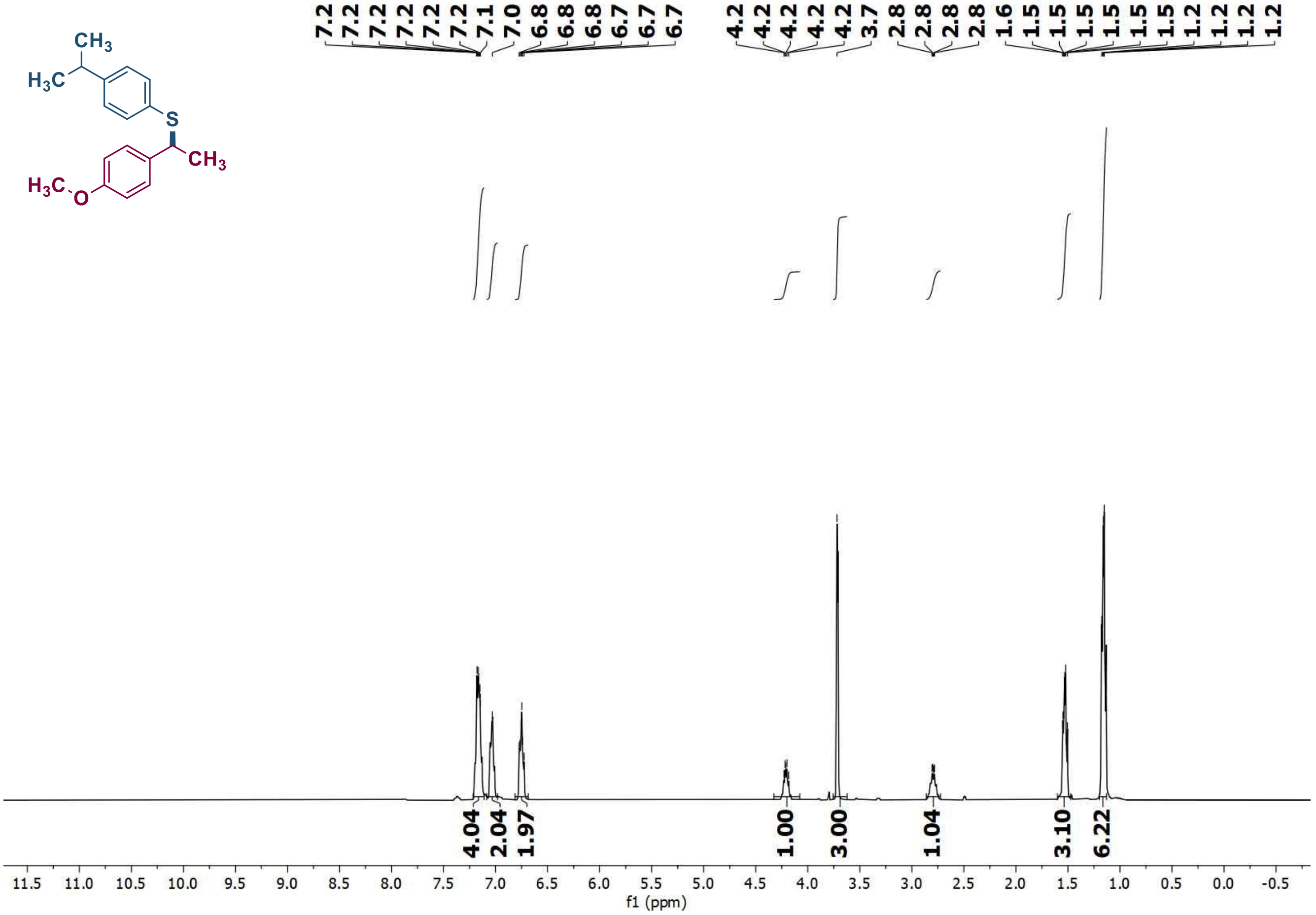

Figure S5. ^{1}H NMR (400 MHz, Chloroform-d, 25°C) of 1-(4-isopropylphenyl)(1-(4-methoxyphenyl)ethyl)sulfane (**3c**).

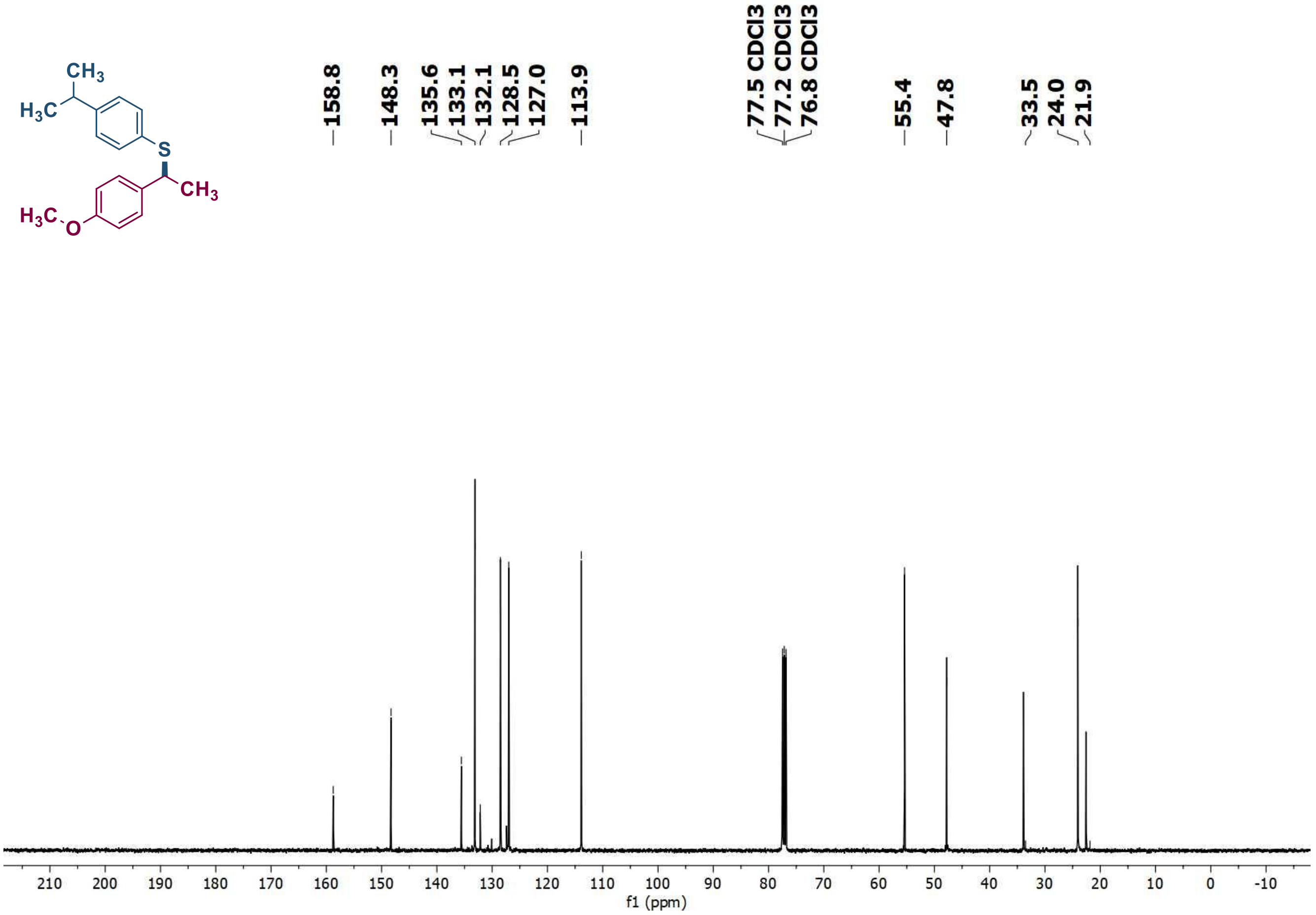

Figure S6. $^{13}C\{^{1}H\}$ NMR (101 MHz, Chloroform-d, 25°C) of 1-(4-isopropylphenyl)(1-(4-methoxyphenyl)ethyl)sulfane (**3c**).

(4-(*Tert*-butyl)phenyl)(1-(4-methoxyphenyl)ethyl)sulfane (**3d**)

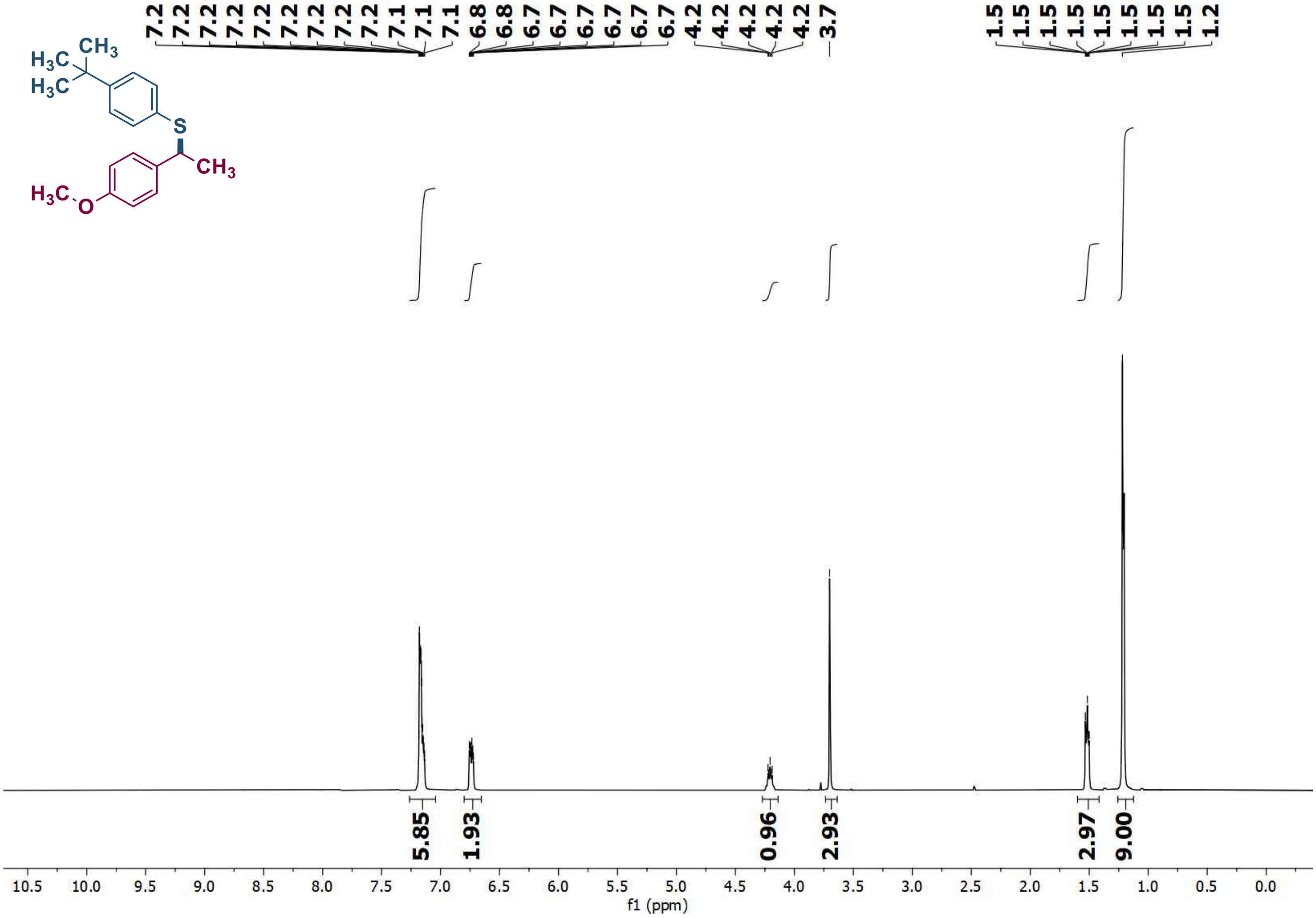

Figure S7. ^{1}H NMR (400 MHz, Chloroform-d, 25°C) of (4-(*tert*-butyl)phenyl)(1-(4-methoxyphenyl)ethyl)sulfane (**3d**).

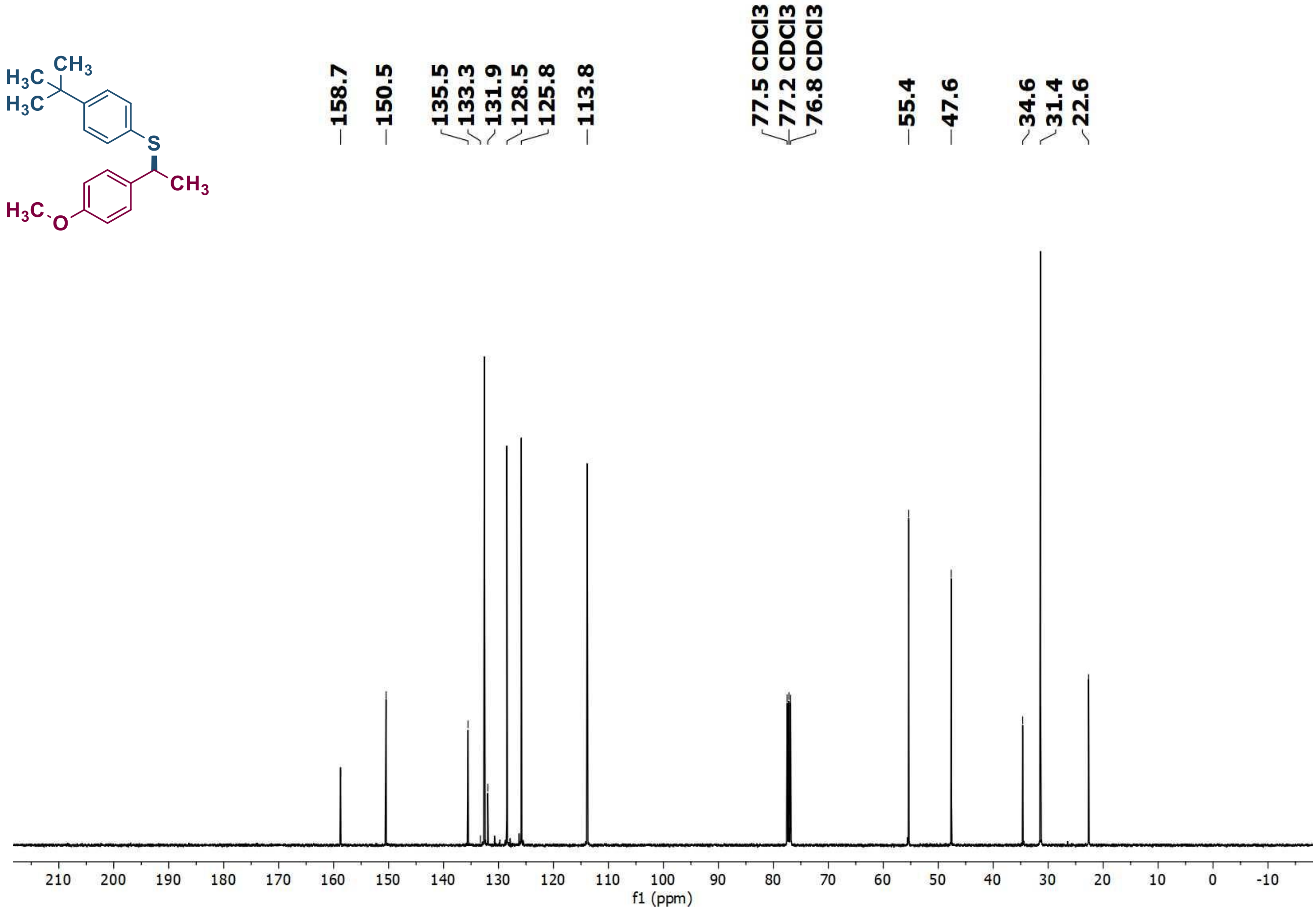

Figure S8. ^{13}C{^{1}H} NMR (101 MHz, Chloroform-d, 25°C) of (4-(*tert*-butyl)phenyl)(1-(4-methoxyphenyl)ethyl)sulfane (**3d**).

(1-(4-Methoxyphenyl)ethyl)(naphthalen-2-yl)sulfane (**3e**)

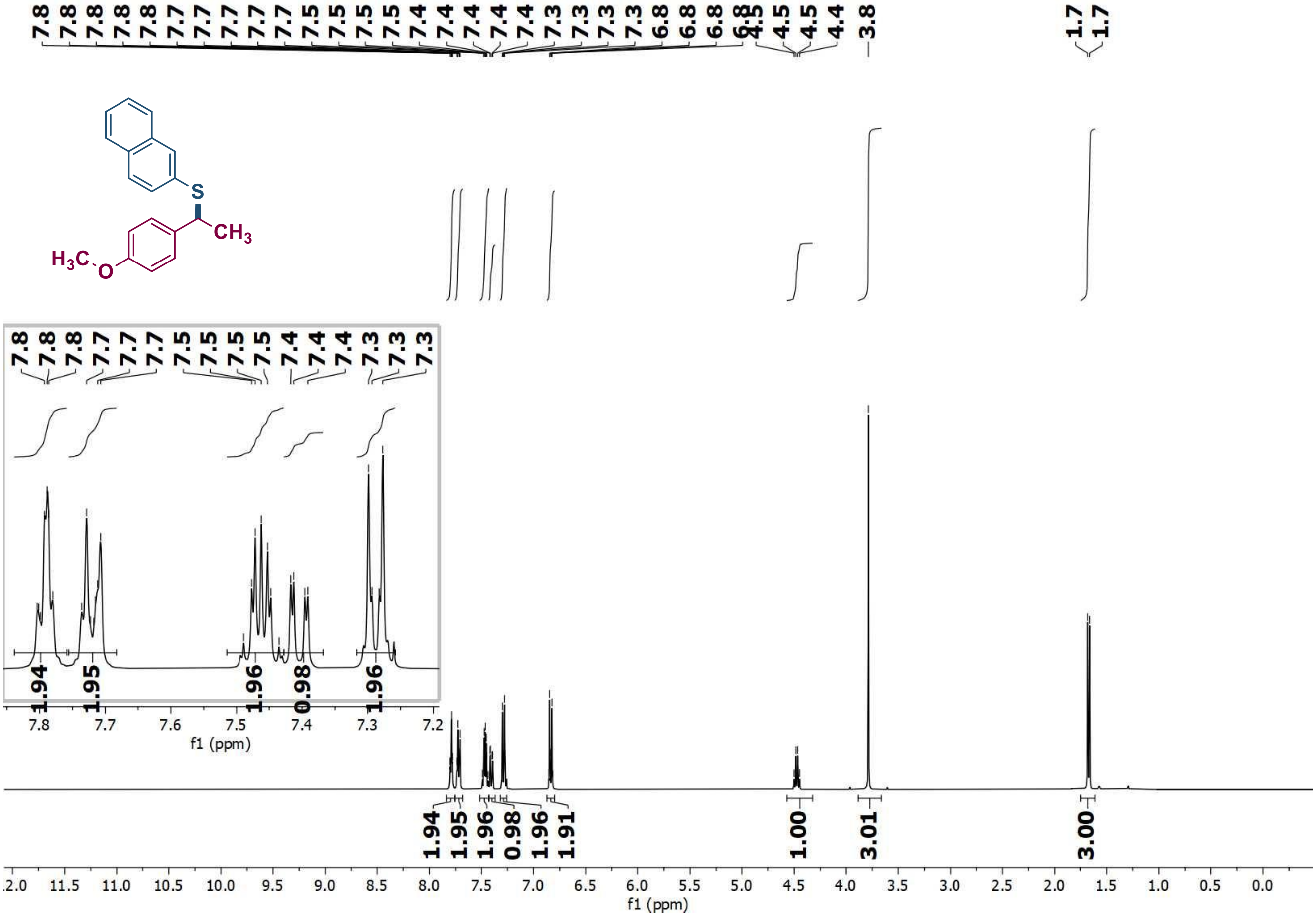

Figure S9. ^{1}H NMR (400 MHz, Chloroform-d, 25°C) of (1-(4-methoxyphenyl)ethyl)(naphthalen-2-yl)sulfane (**3e**)

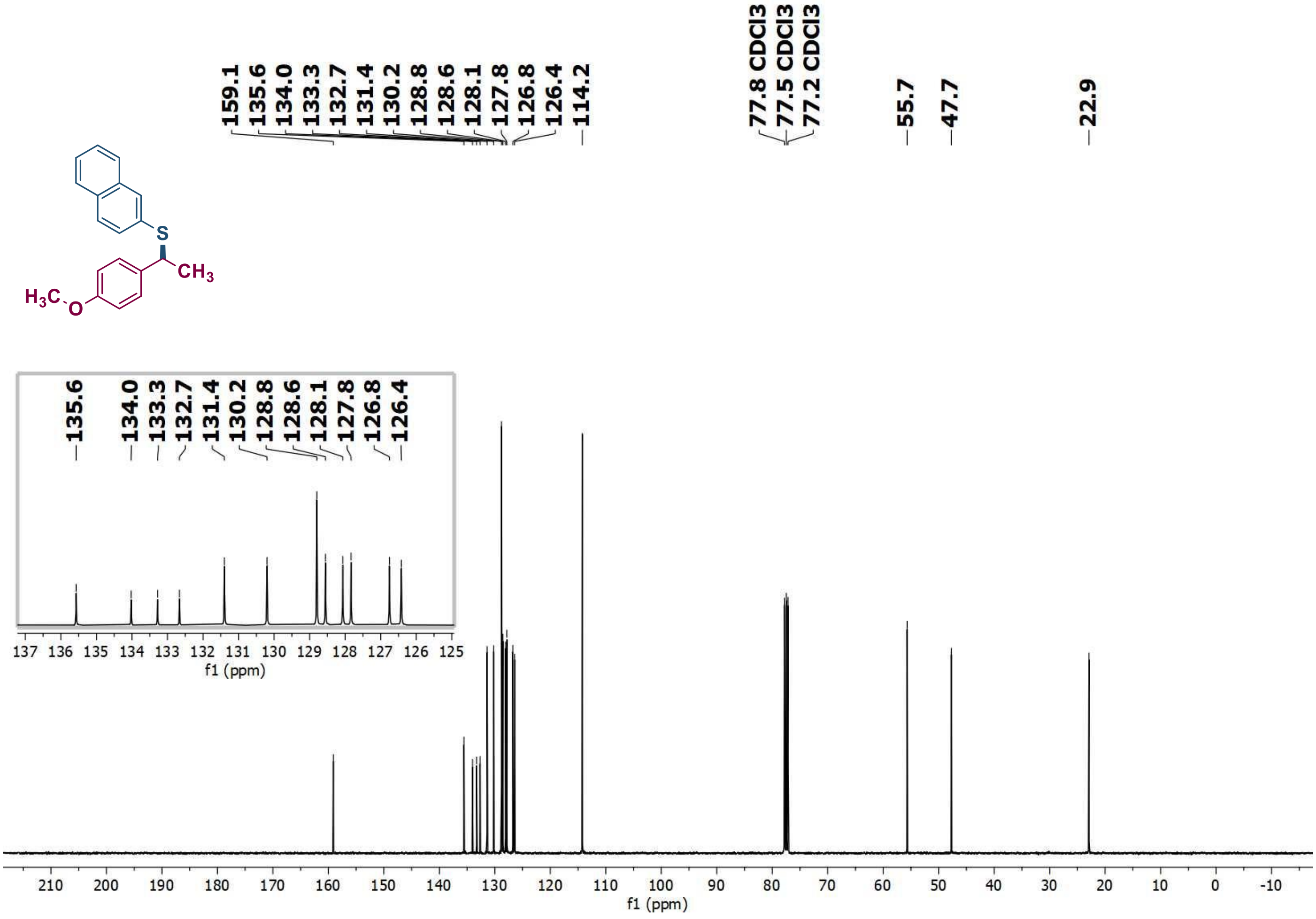

Figure S10. $^{13}C\{^{1}H\}$ NMR (101 MHz, Chloroform-d, 25°C) of (1-(4-Methoxyphenyl)ethyl)(naphthalen-2-yl)sulfane (**3e**)

(4-Ethoxyphenyl)(1-(4-methoxyphenyl)ethyl)sulfane (**3f**)

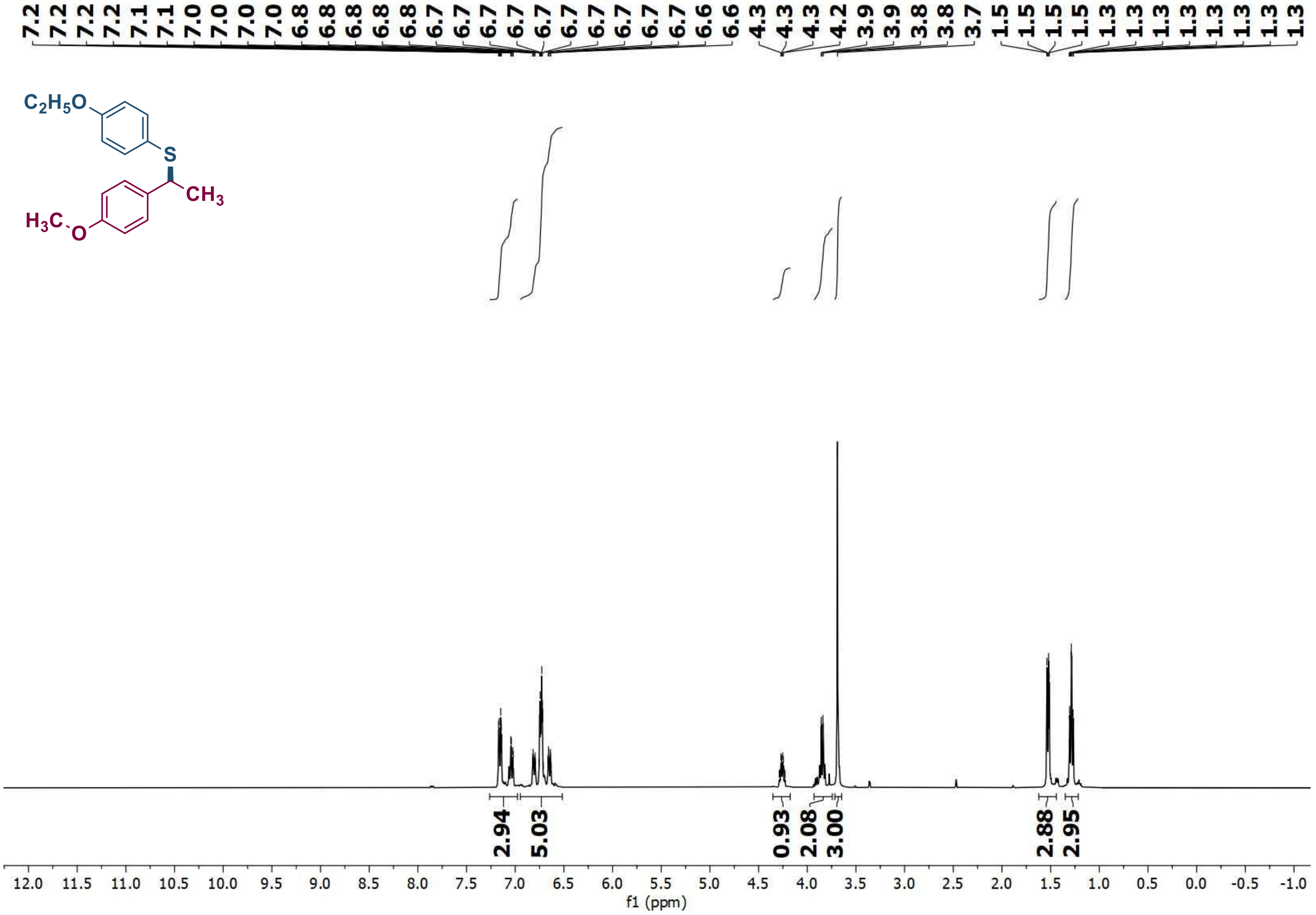

Figure S11. ^{1}H NMR (400 MHz, Chloroform-d, 25°C) of 1(4-ethoxyphenyl)(1-(4-methoxyphenyl)ethyl)sulfane (**3f**).

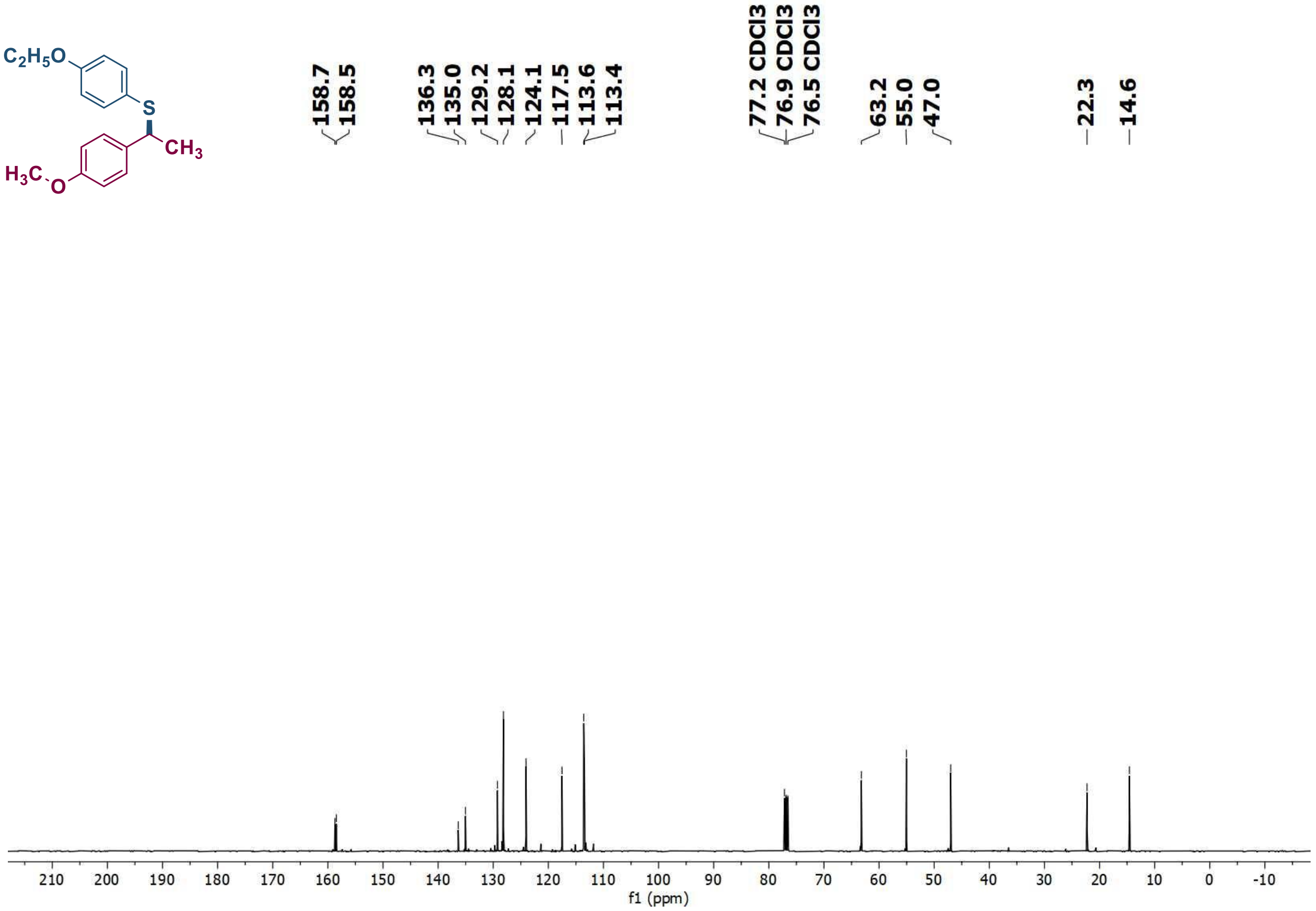

Figure S12. $^{13}C\{^{1}H\}$ NMR (101 MHz, Chloroform-d, 25°C) of 1(4-ethoxyphenyl)(1-(4-methoxyphenyl)ethyl)sulfane (**3f**).

(3-Chloro-4-fluorophenyl)(1-(4-methoxyphenyl)ethyl)sulfane (**3g**)

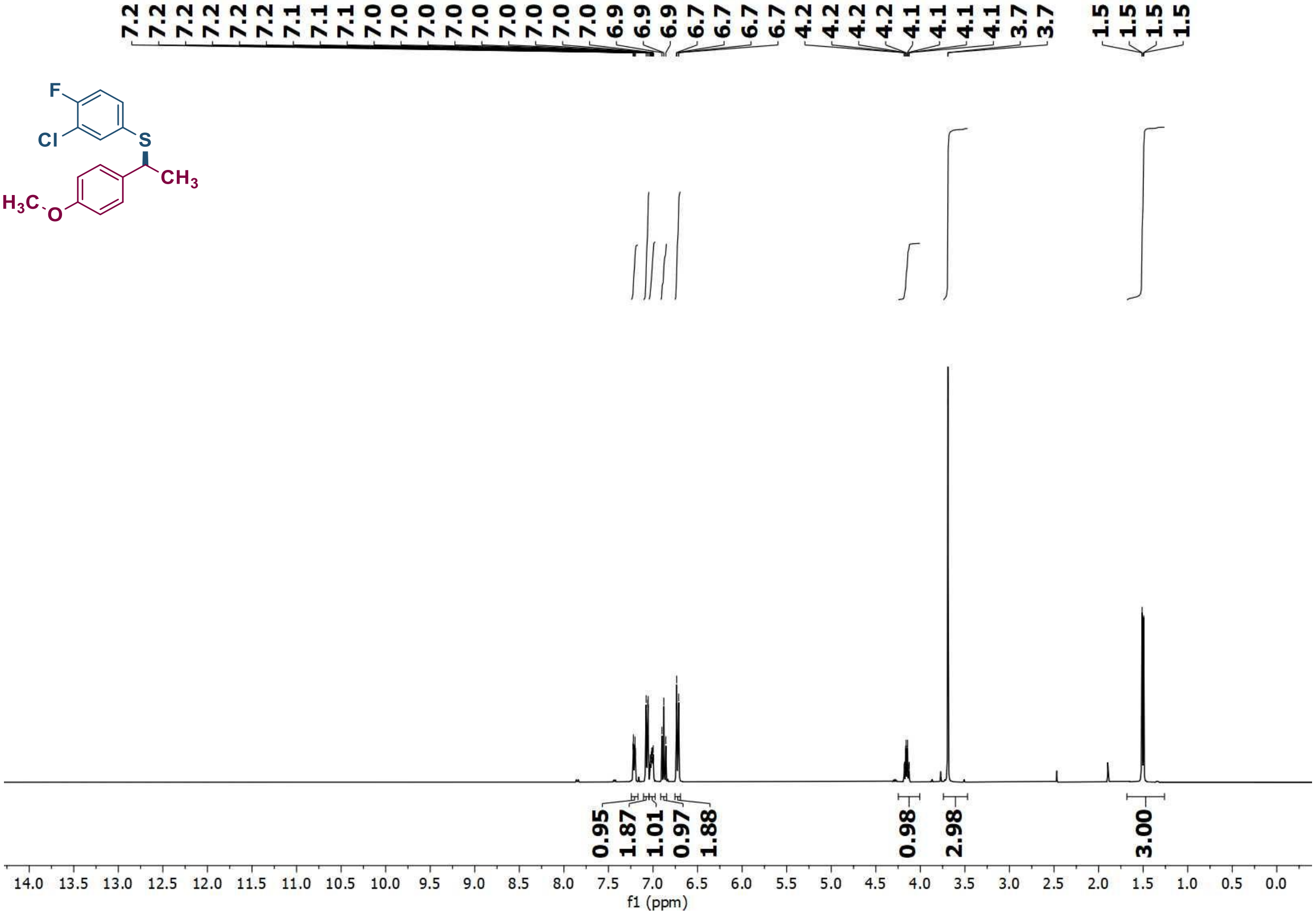

Figure S13. ^{1}H NMR (400 MHz, Chloroform-d, 25°C) of (3-chloro-4-fluorophenyl)(1-(4-methoxyphenyl)ethyl)sulfane (**3g**).

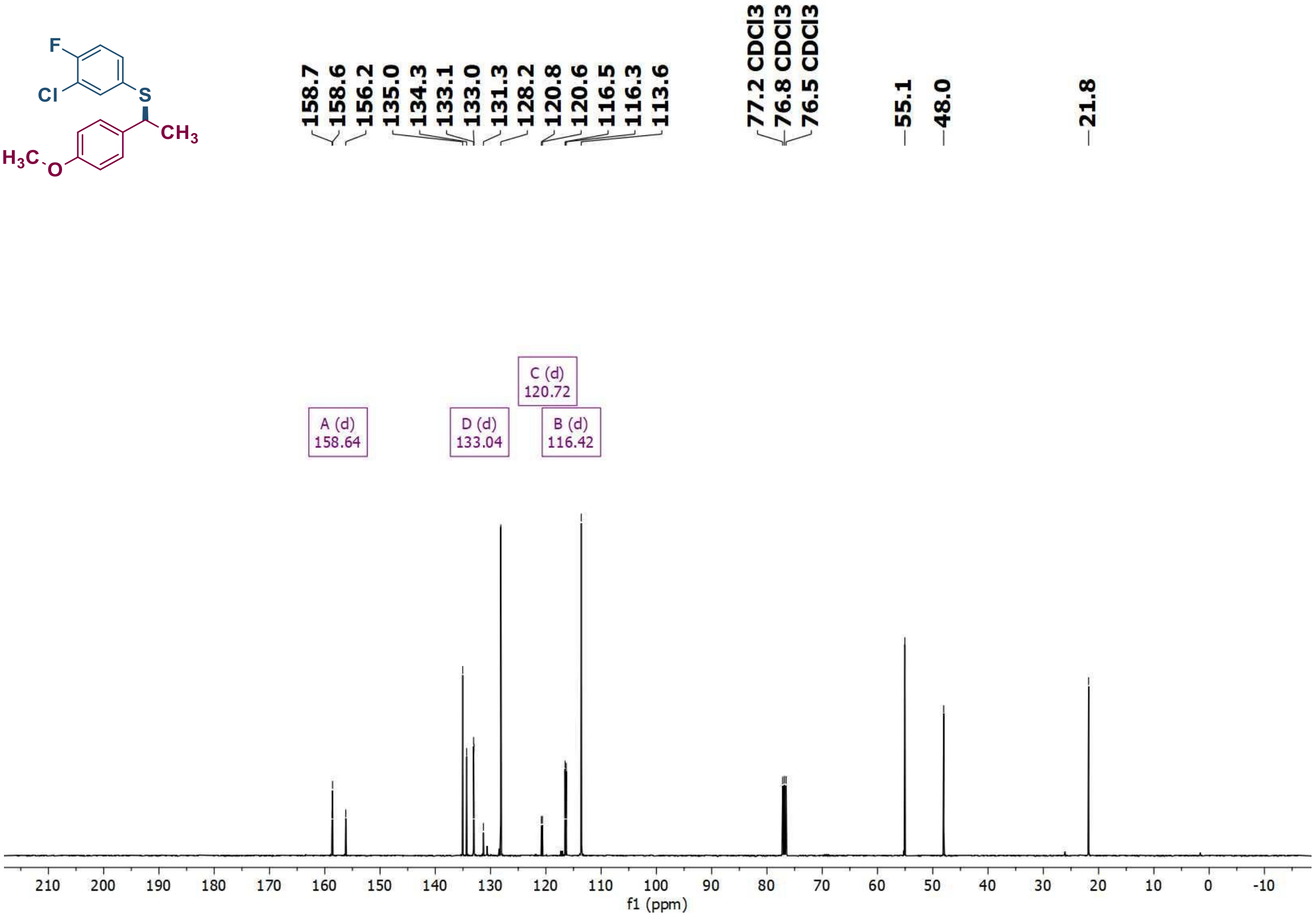

Figure S14. $^{13}C\{^{1}H\}$ NMR (101 MHz, Chloroform-d, 25°C) of of (3-chloro-4-fluorophenyl)(1-(4-methoxyphenyl)ethyl)sulfane (**3g**).

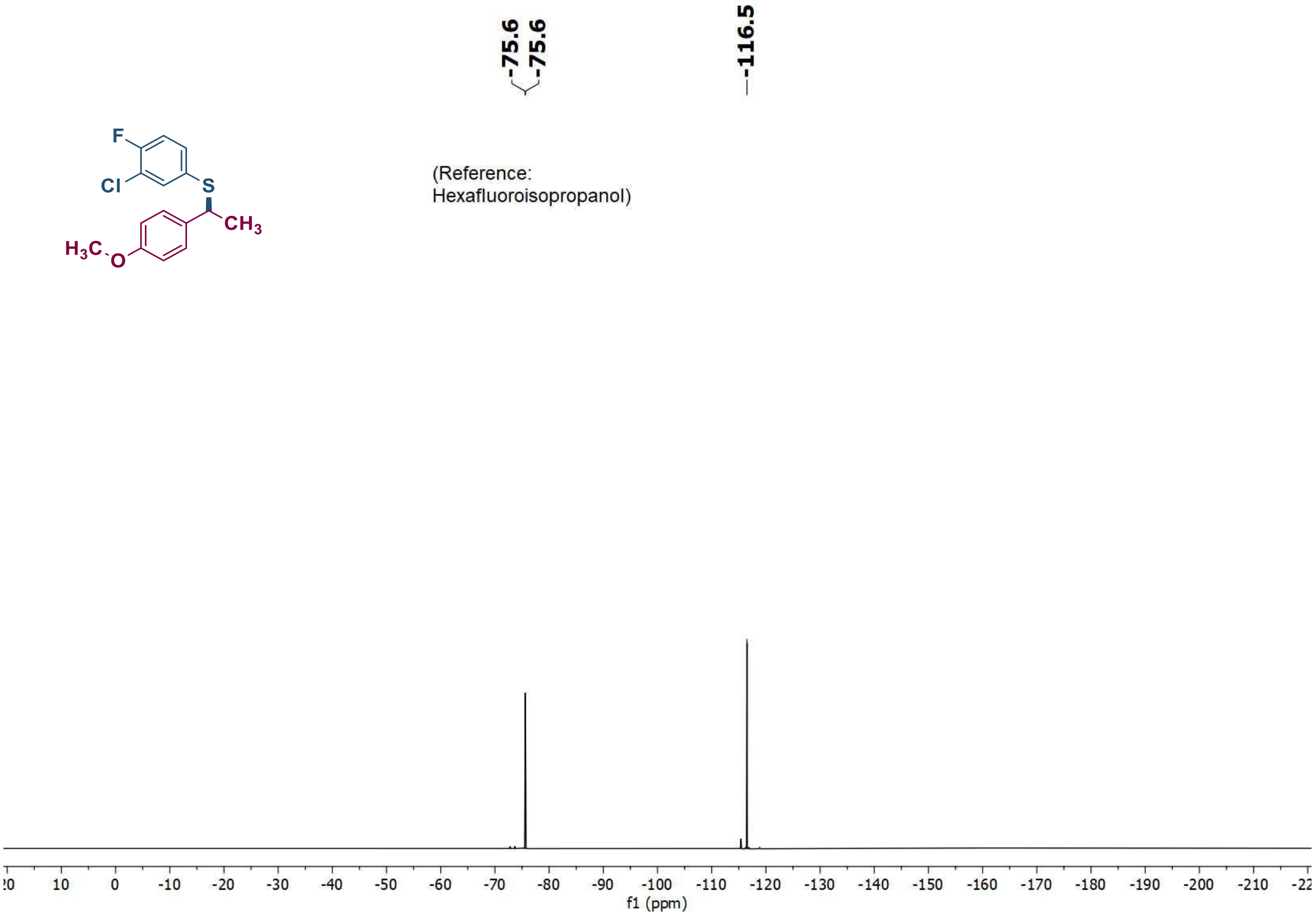

Figure S15. ^{19}F NMR (377 MHz, Chloroform-d, 25°C) of of (3-chloro-4-fluorophenyl)(1-(4-methoxyphenyl)ethyl)sulfane (**3g**).

(4-Chlorophenyl)(1-(4-methoxyphenyl)ethyl)sulfane (**3h**)

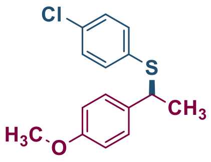

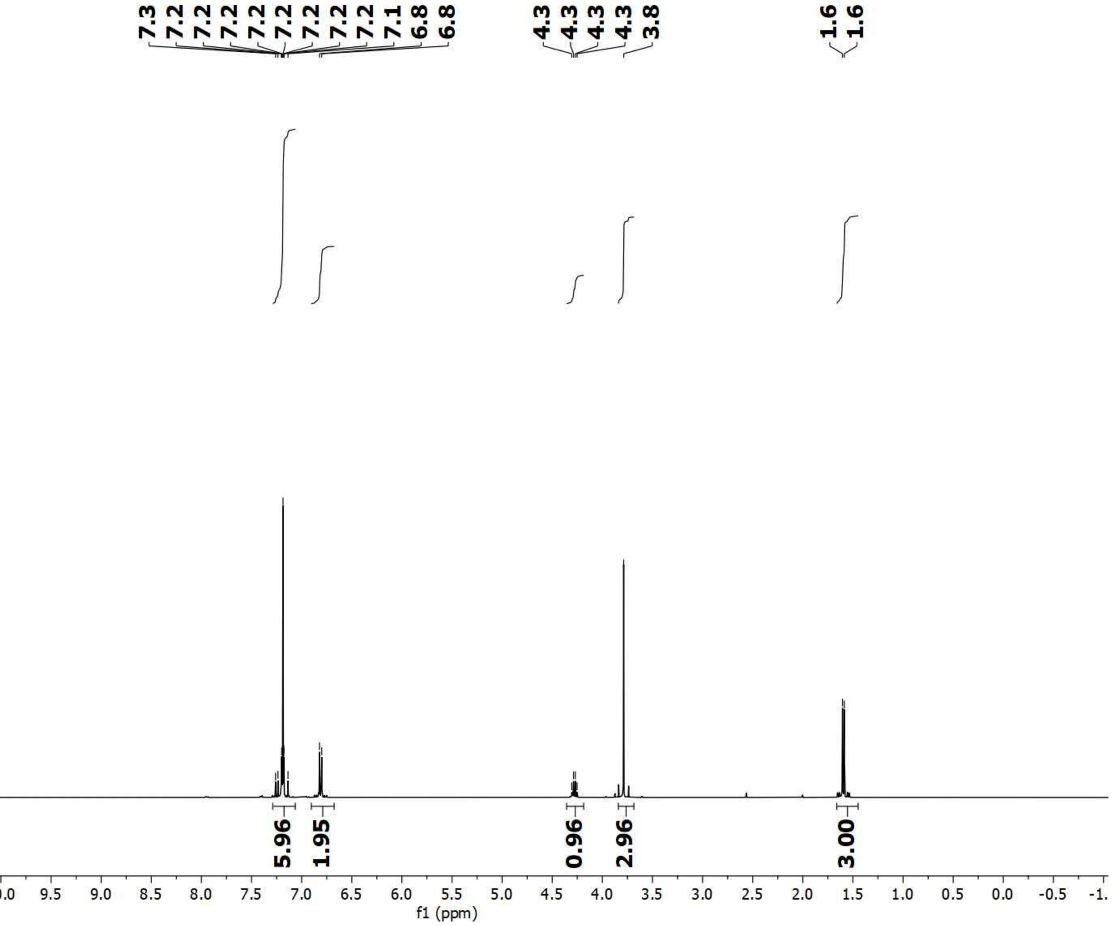

Figure S16. ^{1}H NMR (400 MHz, Chloroform-d, 25°C) of (4-chlorophenyl)(1-(4-methoxyphenyl)ethyl)sulfane (**3h**).

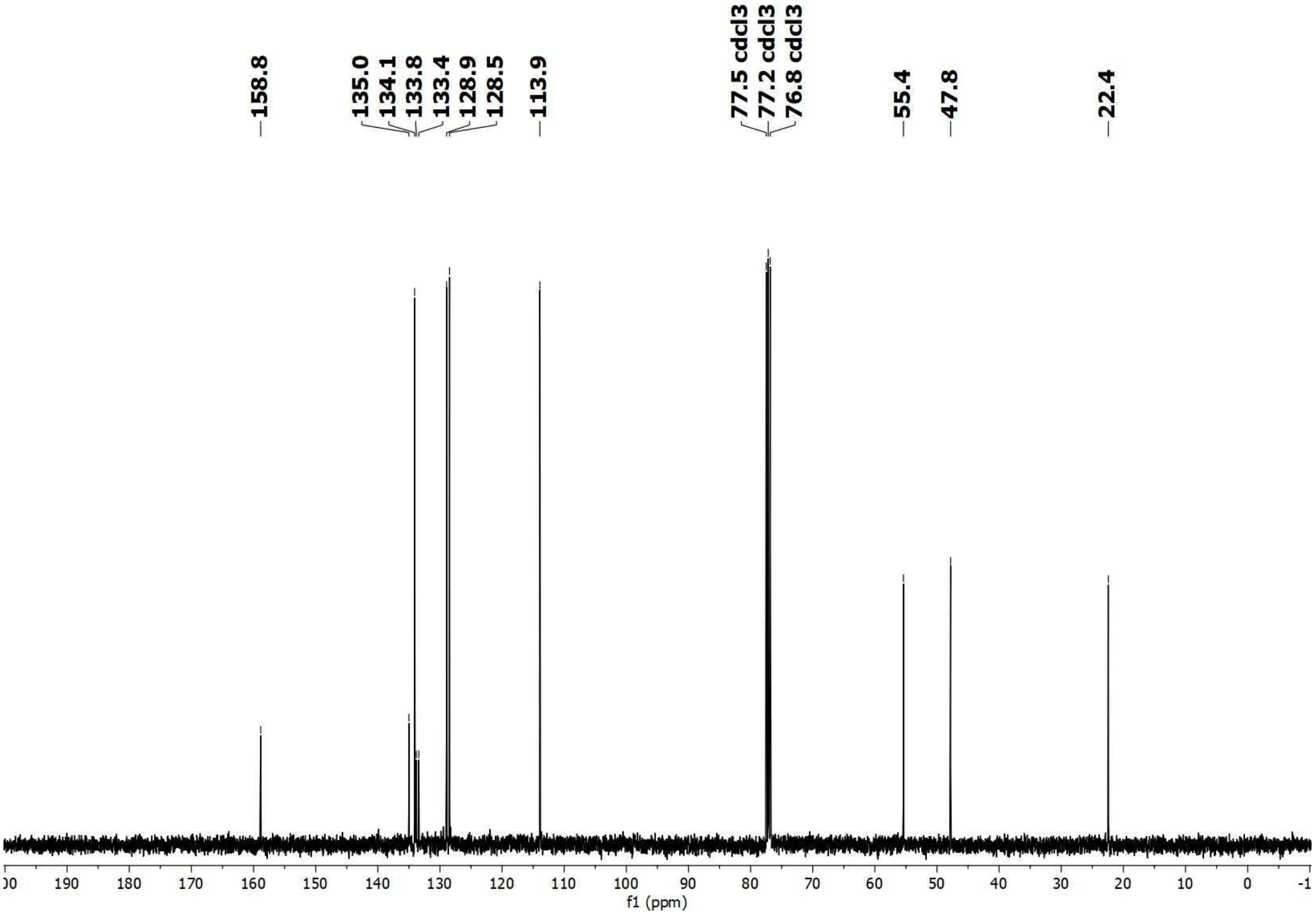

Figure S17. $^{13}C\{^{1}H\}$ NMR (101 MHz, Chloroform-d, 25°C) of (4-chlorophenyl)(1-(4-methoxyphenyl)ethyl)sulfane (**3h**).

(4-Bromophenyl)(1-(4-methoxyphenyl)ethyl)sulfane (**3i**)

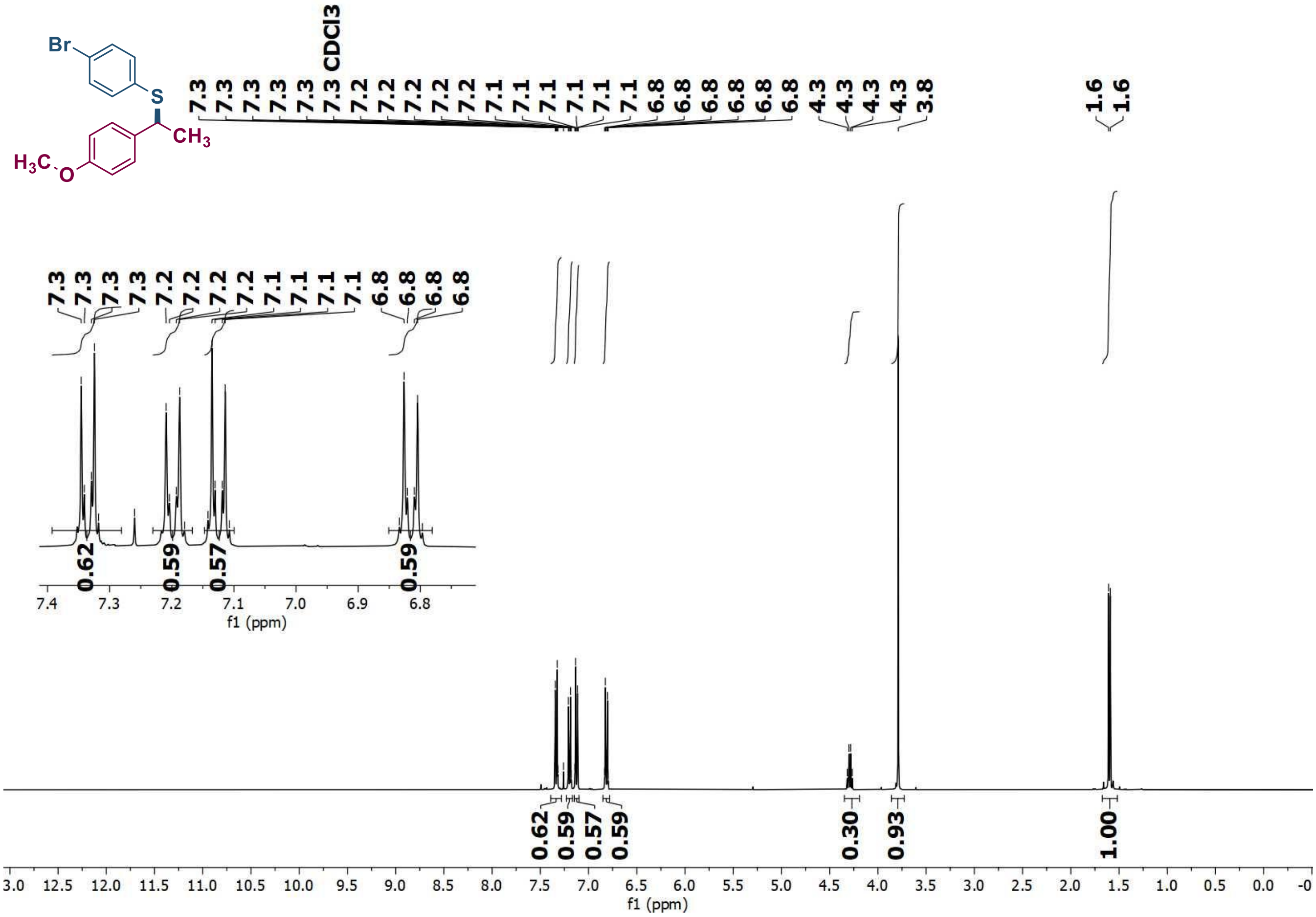

Figure S18. ^{1}H NMR (400 MHz, Chloroform-d, 25°C) of (4-bromophenyl)(1-(4-methoxyphenyl)ethyl)sulfane (**3i**).

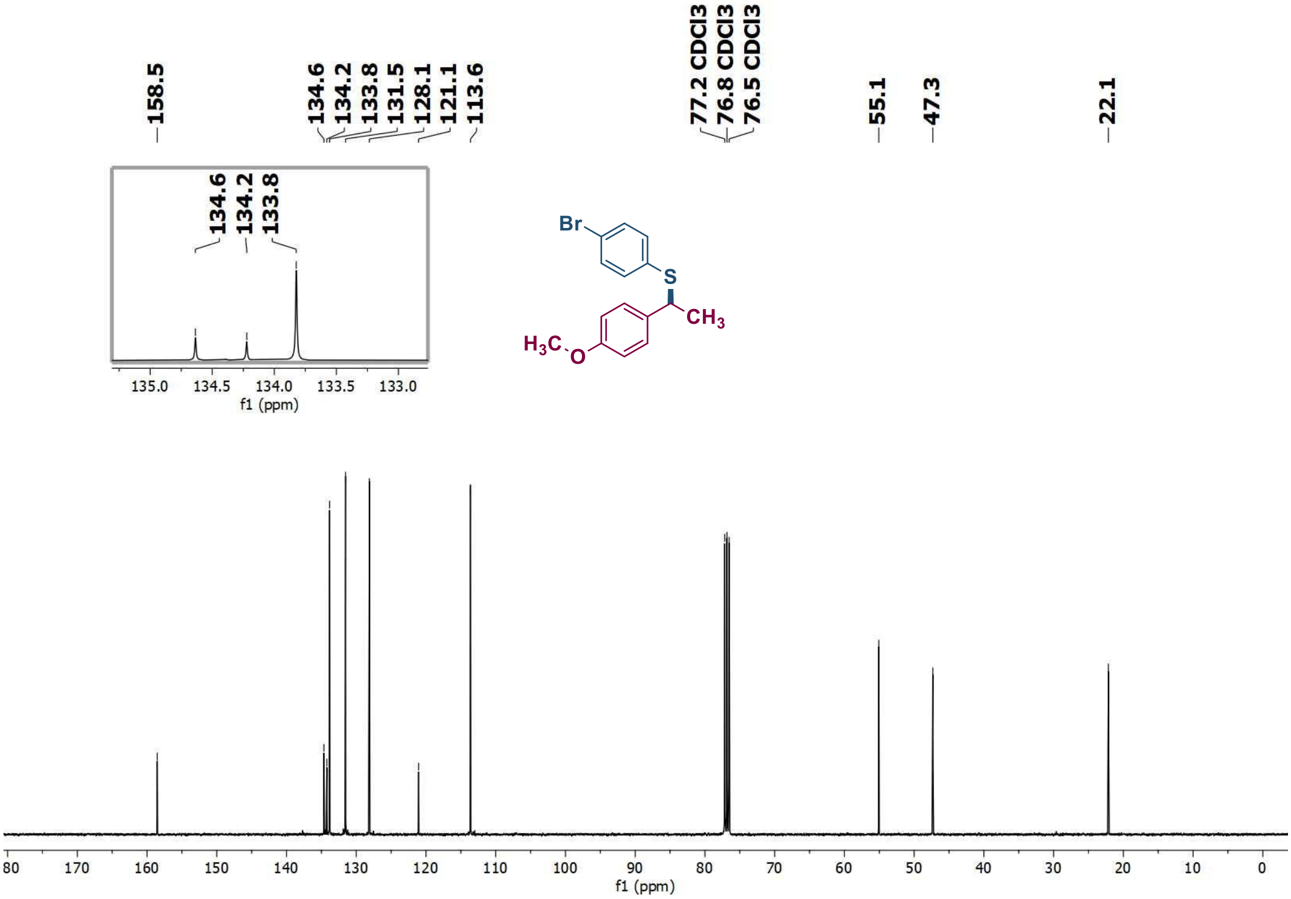

Figure S19. ^{13}C{^{1}H} NMR (101 MHz, Chloroform-d, 25°C) of (4-bromophenyl)(1-(4-methoxyphenyl)ethyl)sulfane (**3i**).

(1-(4-Methoxyphenyl)ethyl)(4-(trifluoromethoxy)phenyl)sulfane (**3j**)

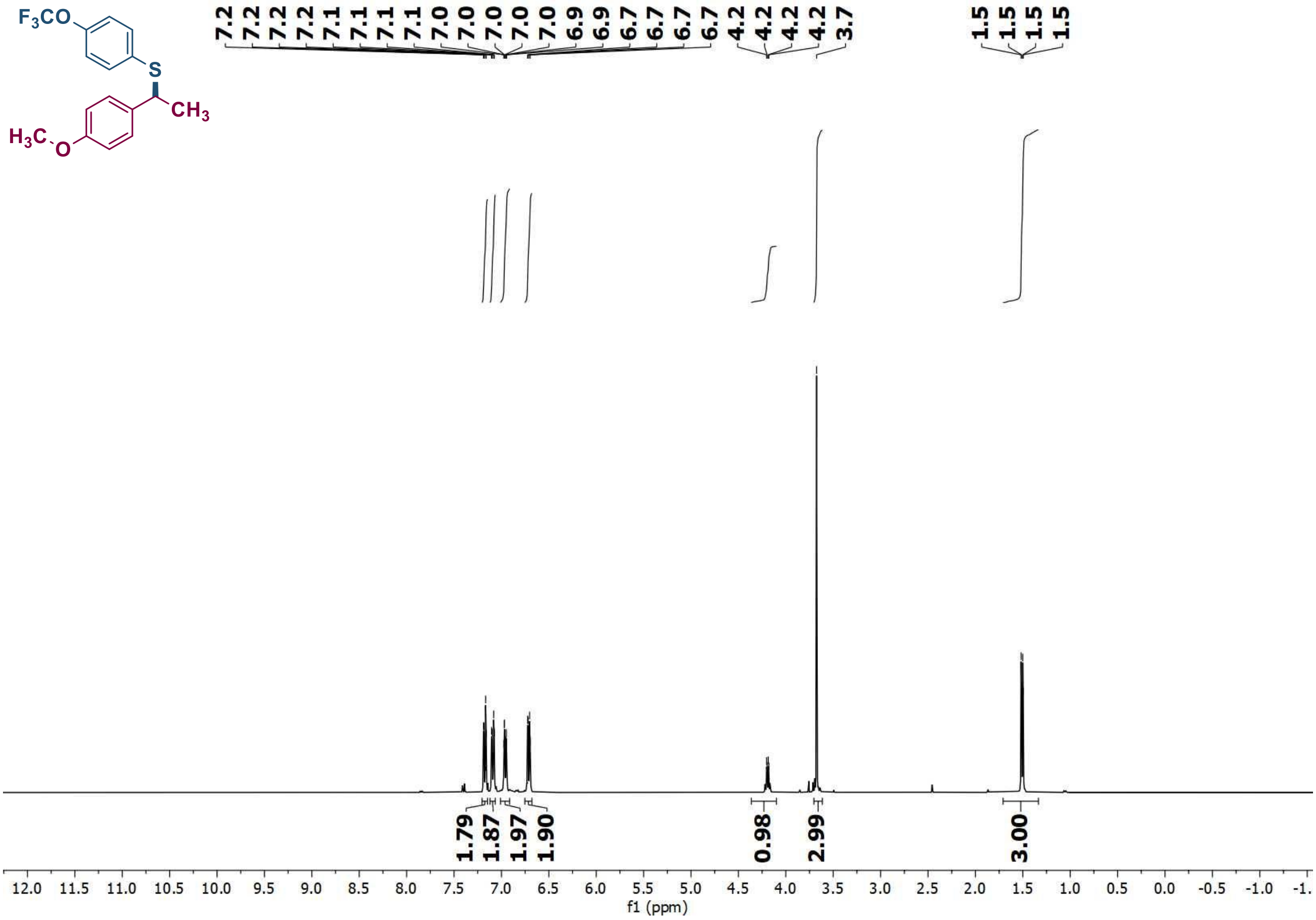

Figure S20. ^{1}H NMR (400 MHz, Chloroform-d, 25°C) of (1-(4-methoxyphenyl)ethyl)(4-(trifluoromethoxy)phenyl)sulfane (**3j**).

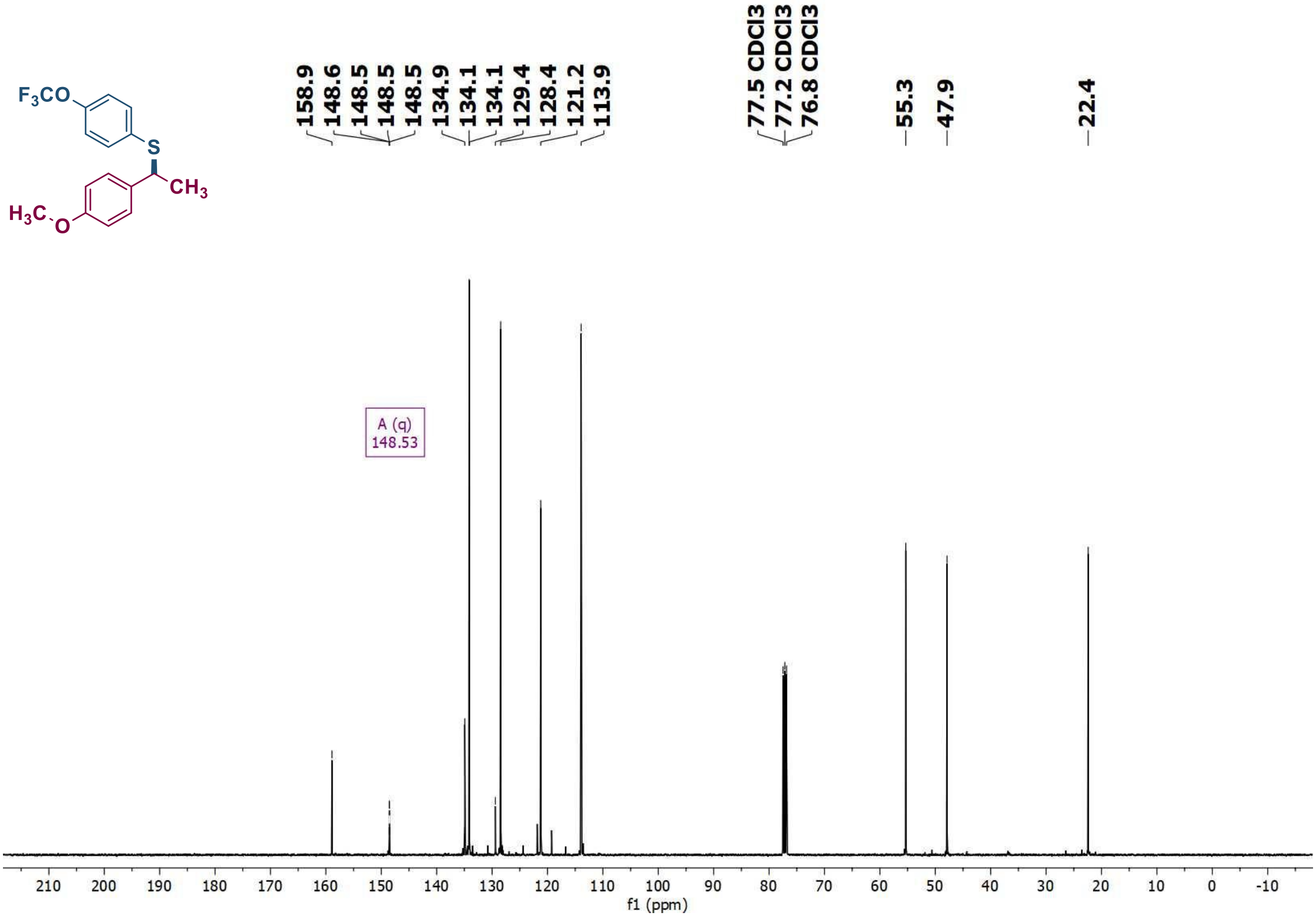

Figure S21. ^{13}C{^{1}H} NMR (101 MHz, Chloroform-d, 25°C) of (1-(4-methoxyphenyl)ethyl)(4-(trifluoromethoxy)phenyl)sulfane (**3j**).

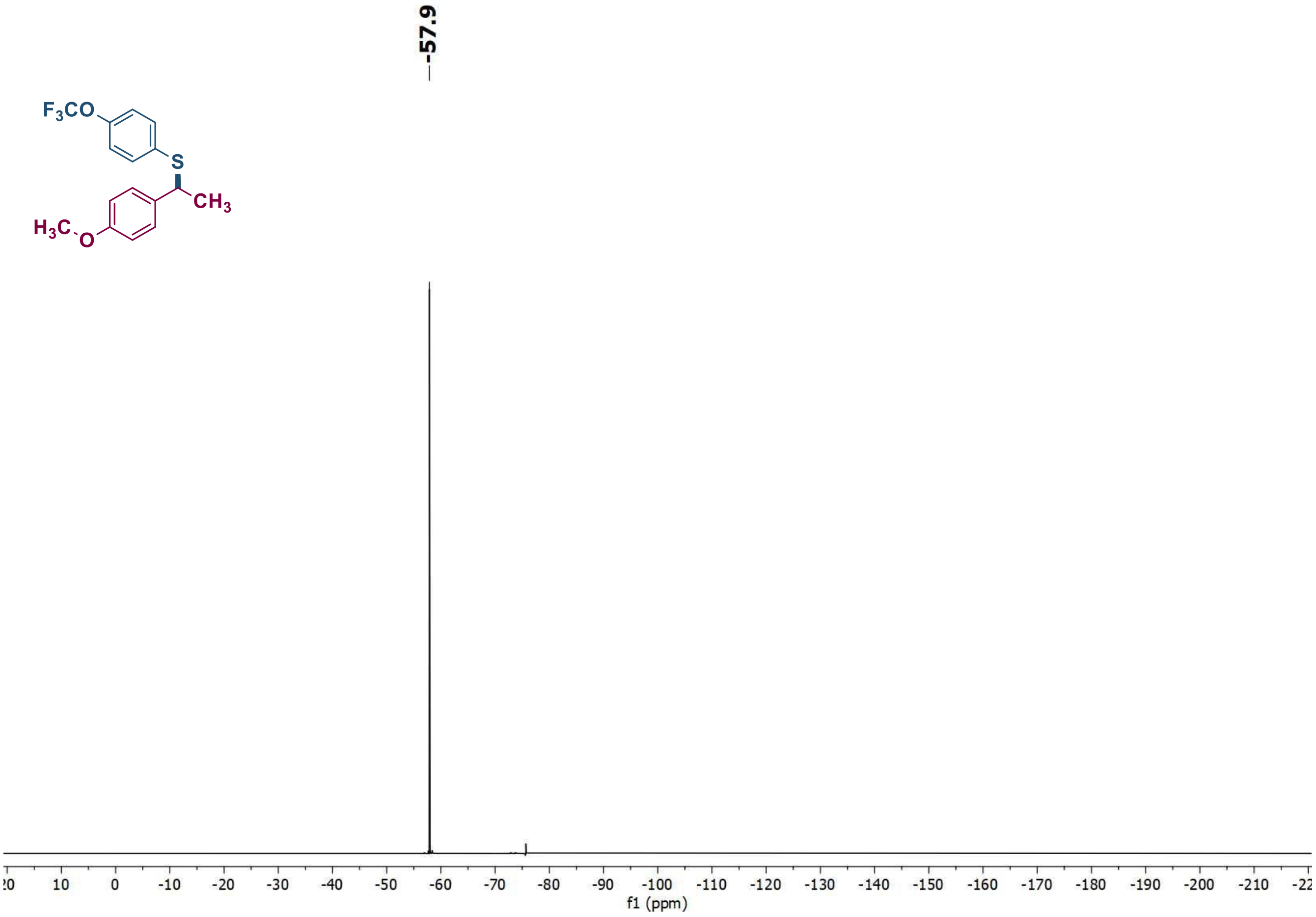

Figure S22. ^{19}F NMR (377 MHz, Chloroform-d, 25°C) of (1-(4-methoxyphenyl)ethyl)(4-(trifluoromethoxy)phenyl)sulfane (**3j**).

(1-(4-Methoxyphenyl)ethyl)(2-(trifluoromethyl)phenyl)sulfane (**3k**)

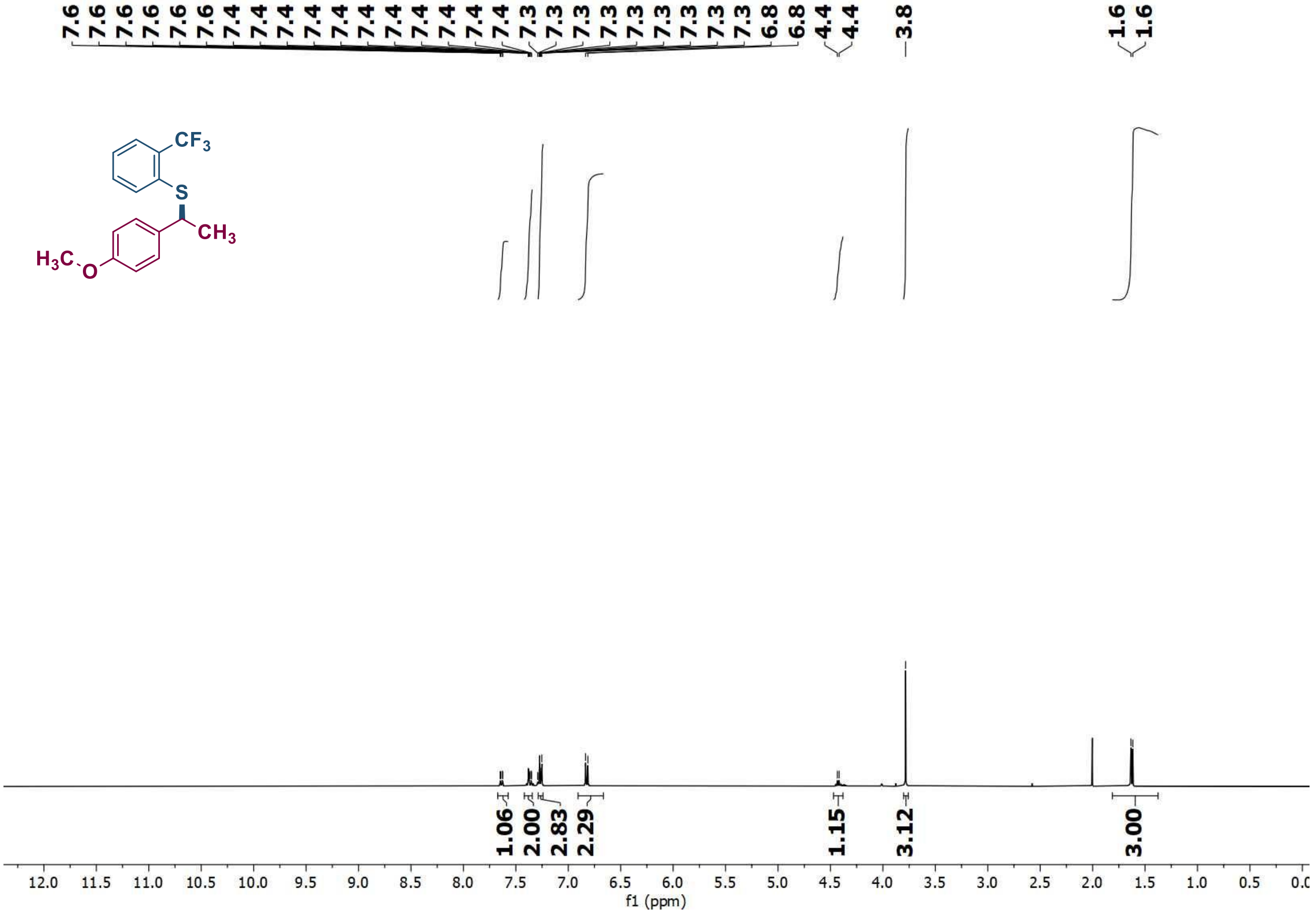

Figure S23. ^{1}H NMR (400 MHz, Chloroform-d, 25°C) of (1-(4-methoxyphenyl)ethyl)(2-(trifluoromethyl)phenyl)sulfane (**3k**).

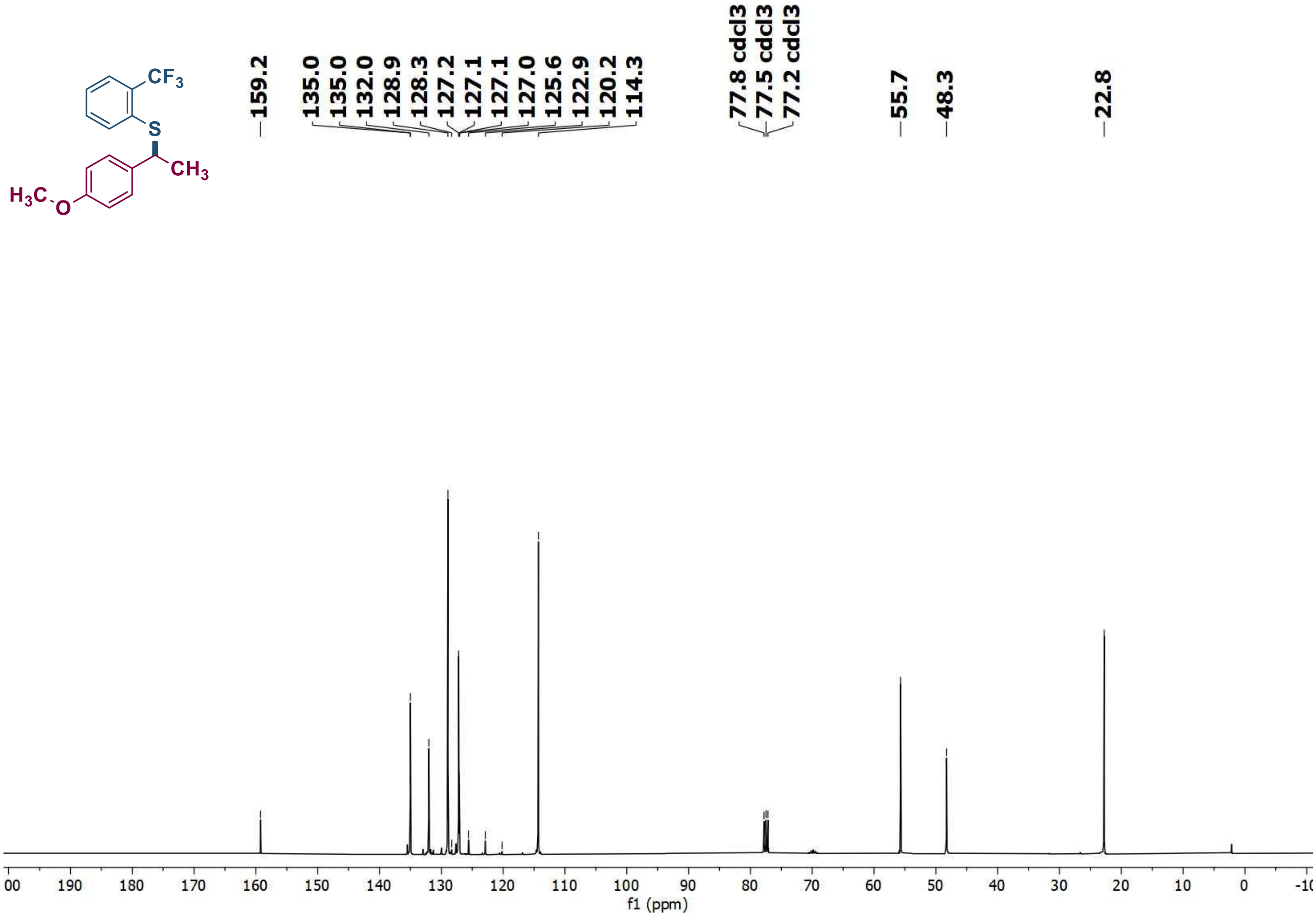

Figure S24. $^{13}C\{^{1}H\}$ NMR (101 MHz, Chloroform-d, 25°C) of (1-(4-Methoxyphenyl)ethyl)(2-(trifluoromethyl)phenyl)sulfane (**3k**).

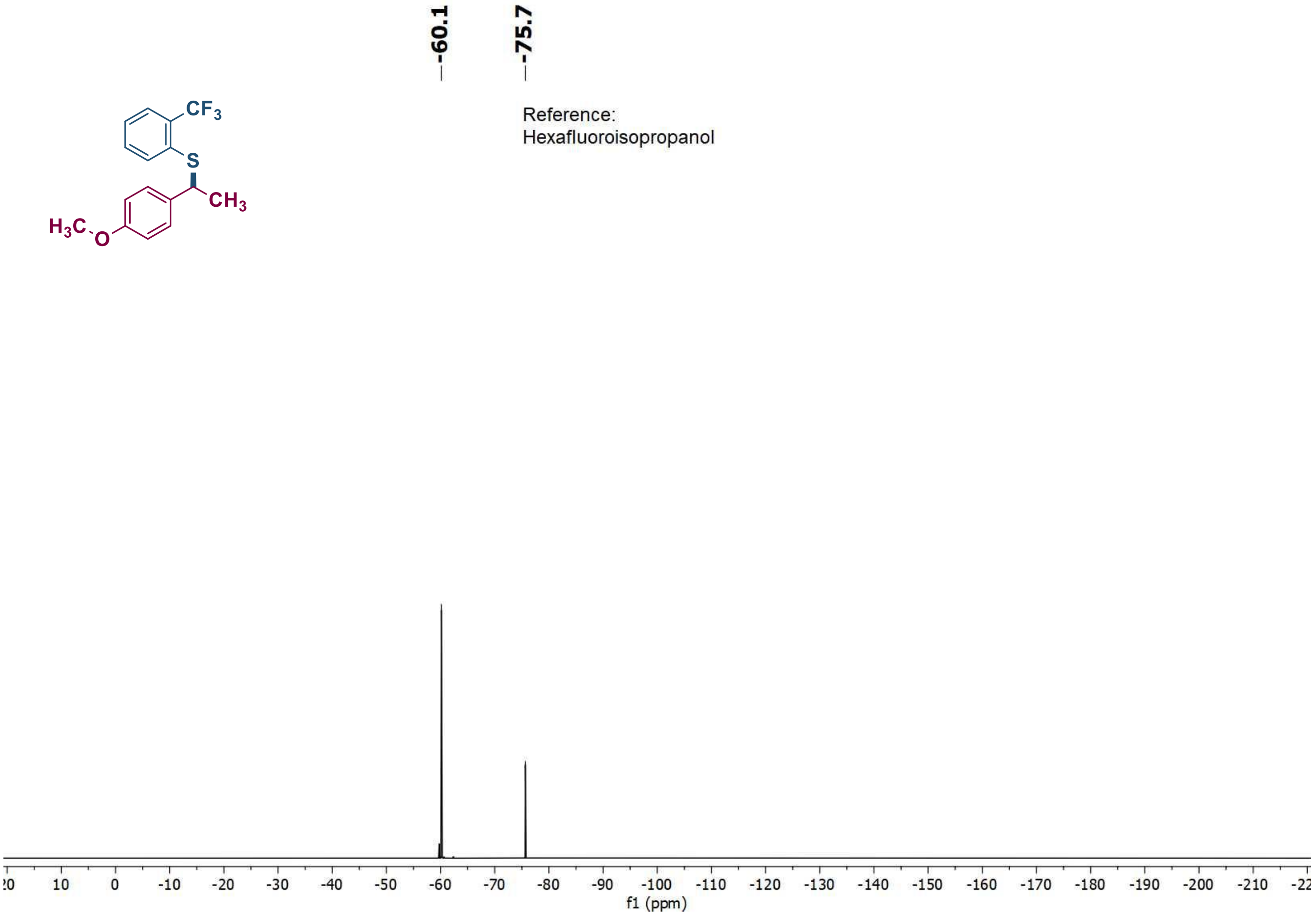

Figure S25. ^{19}F NMR (377 MHz, Chloroform-d, 25°C) of (1-(4-Methoxyphenyl)ethyl)(2-(trifluoromethyl)phenyl)sulfane (**3k).**

5-Fluoro-2-((1-(4-methoxyphenyl)ethyl)thio)benzo[d]oxazole (**3l**)

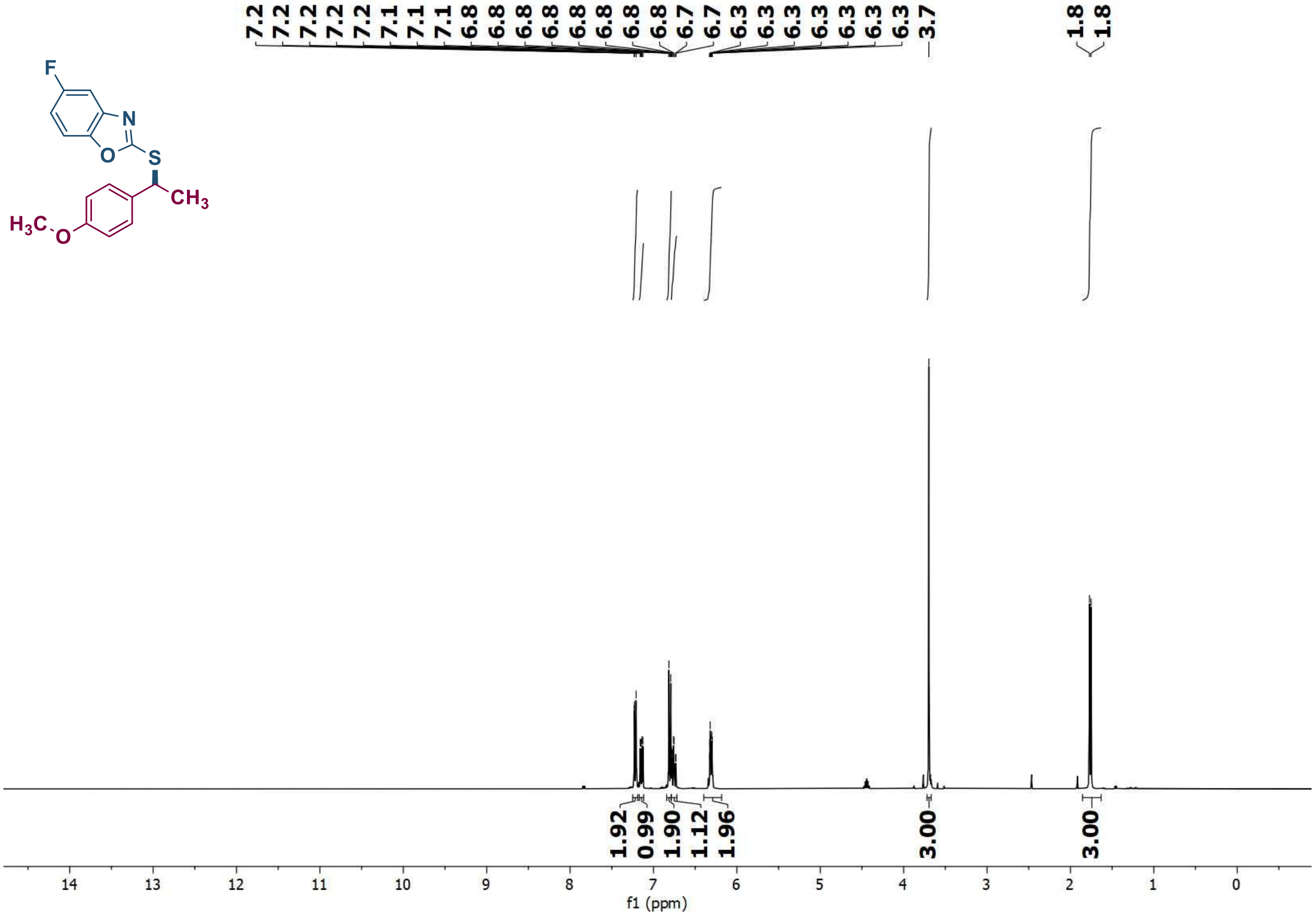

Figure S26. ^{1}H NMR (400 MHz, Chloroform-d, 25°C) of 5-fluoro-2-((1-(4-methoxyphenyl)ethyl)thio)benzo[d]oxazole (**3l**).

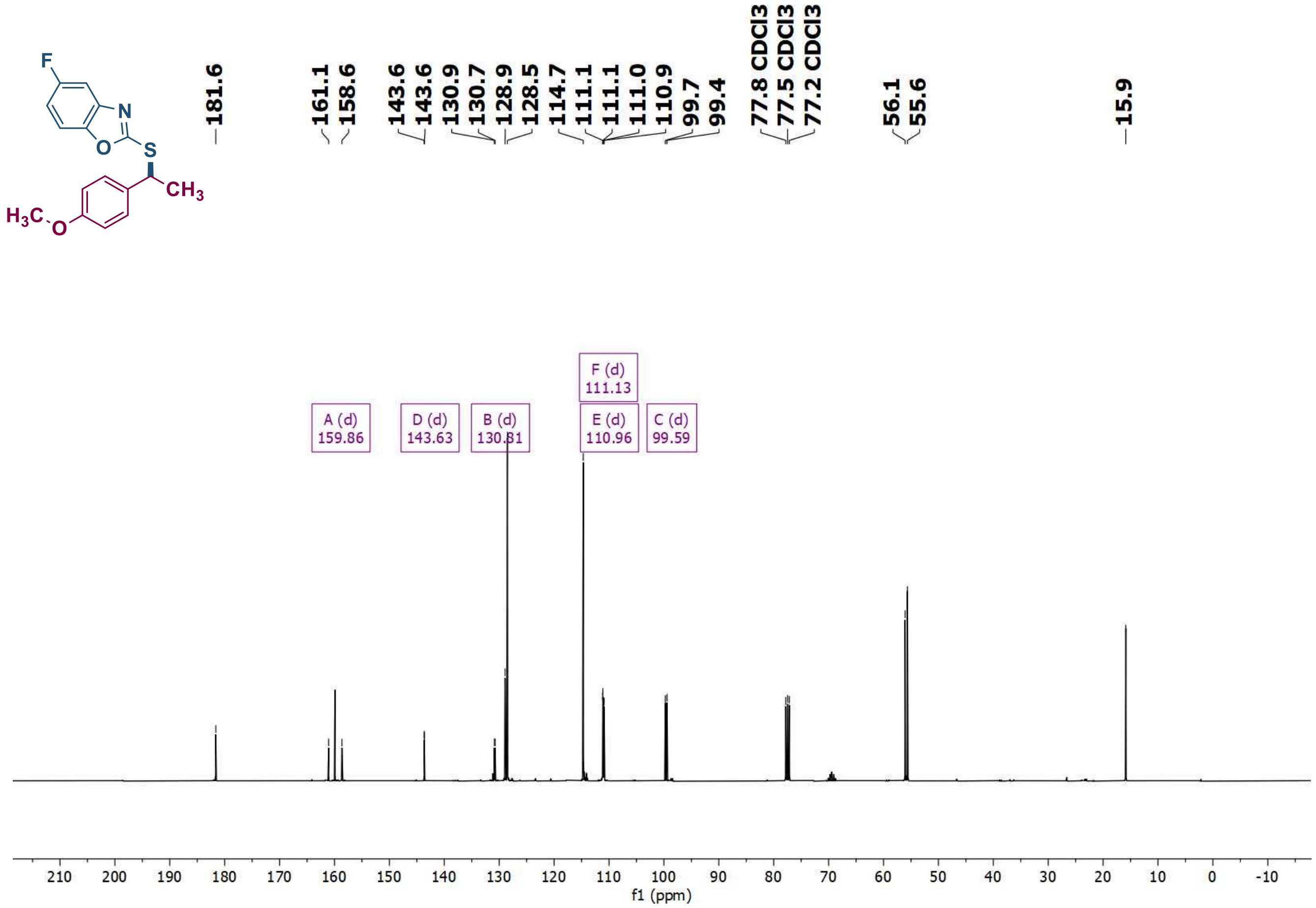

Figure S27. $^{13}C\{^{1}H\}$ NMR (101 MHz, Chloroform-d, 25°C) of 5-fluoro-2-((1-(4-methoxyphenyl)ethyl)thio)benzo[d]oxazole (**3l**).

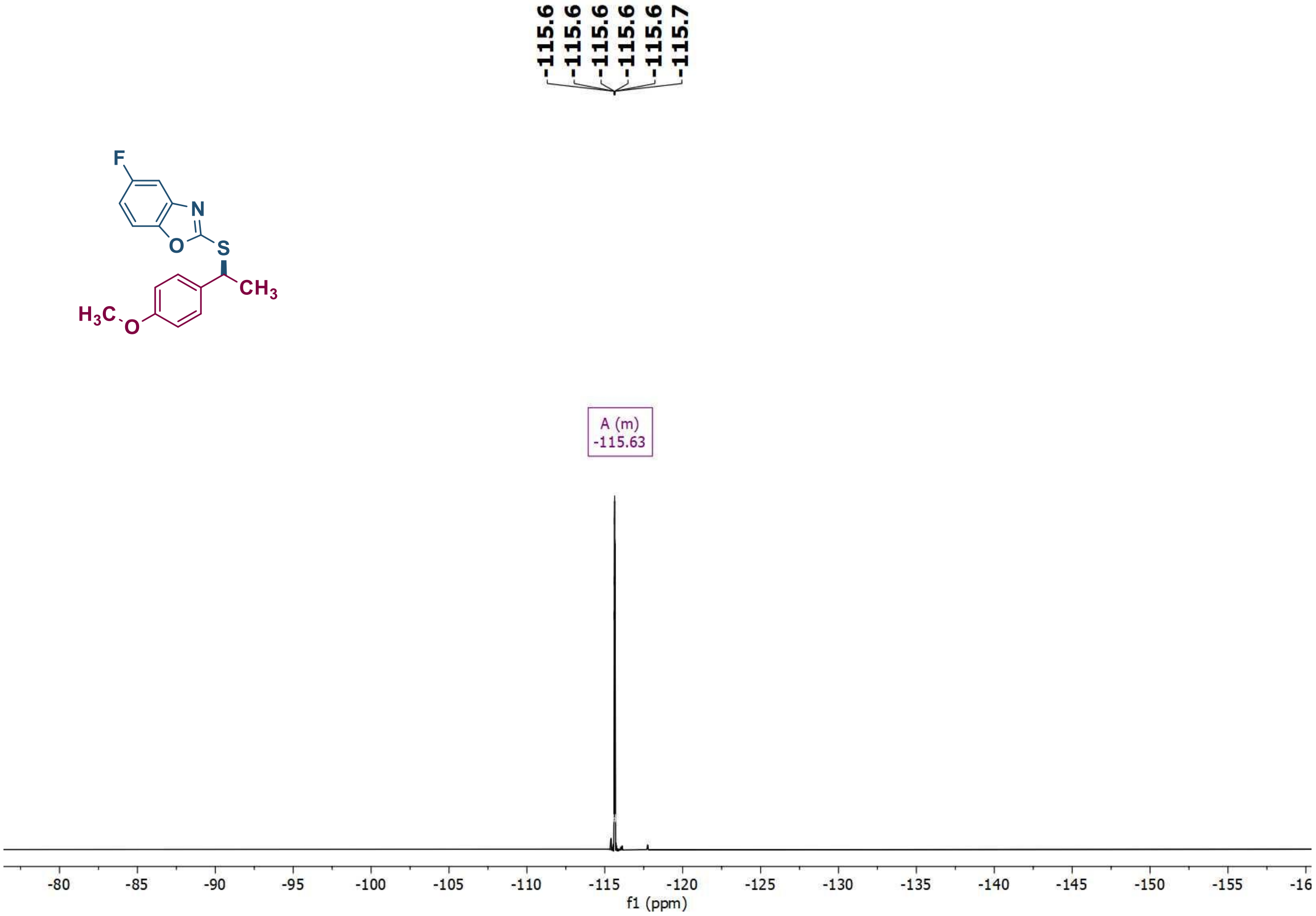

Figure S28. ^{19}F NMR (377 MHz, Chloroform-d, 25°C) of 5-fluoro-2-((1-(4-methoxyphenyl)ethyl)thio)benzo[d]oxazole (**3l**).

2-((1-(4-Methoxyphenyl)ethyl)thio)thiophene (**3m**)

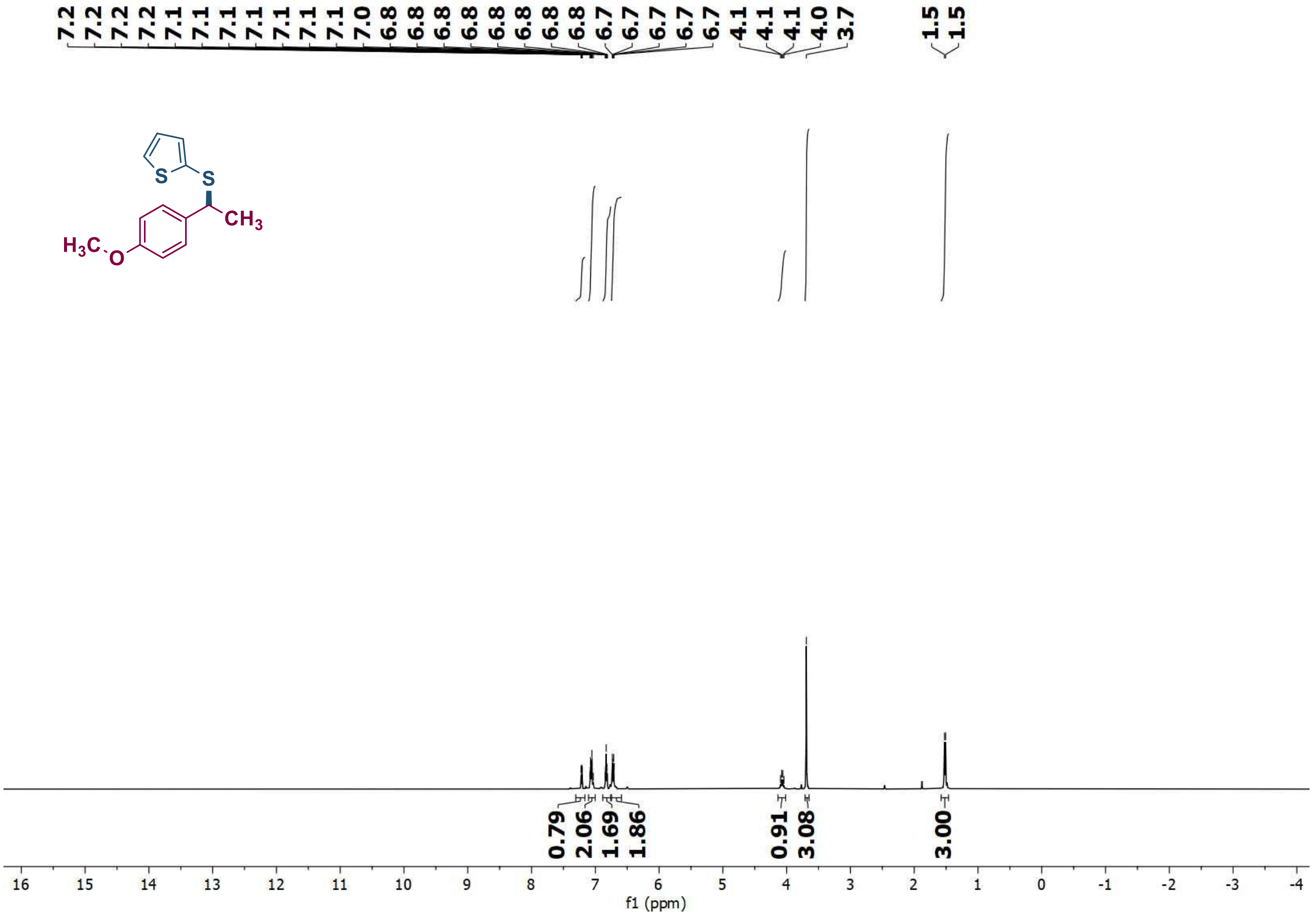

Figure S29. ^{1}H NMR (400 MHz, Chloroform-d, 25°C) of 2-((1-(4-methoxyphenyl)ethyl)thio)thiophene (**3m**).

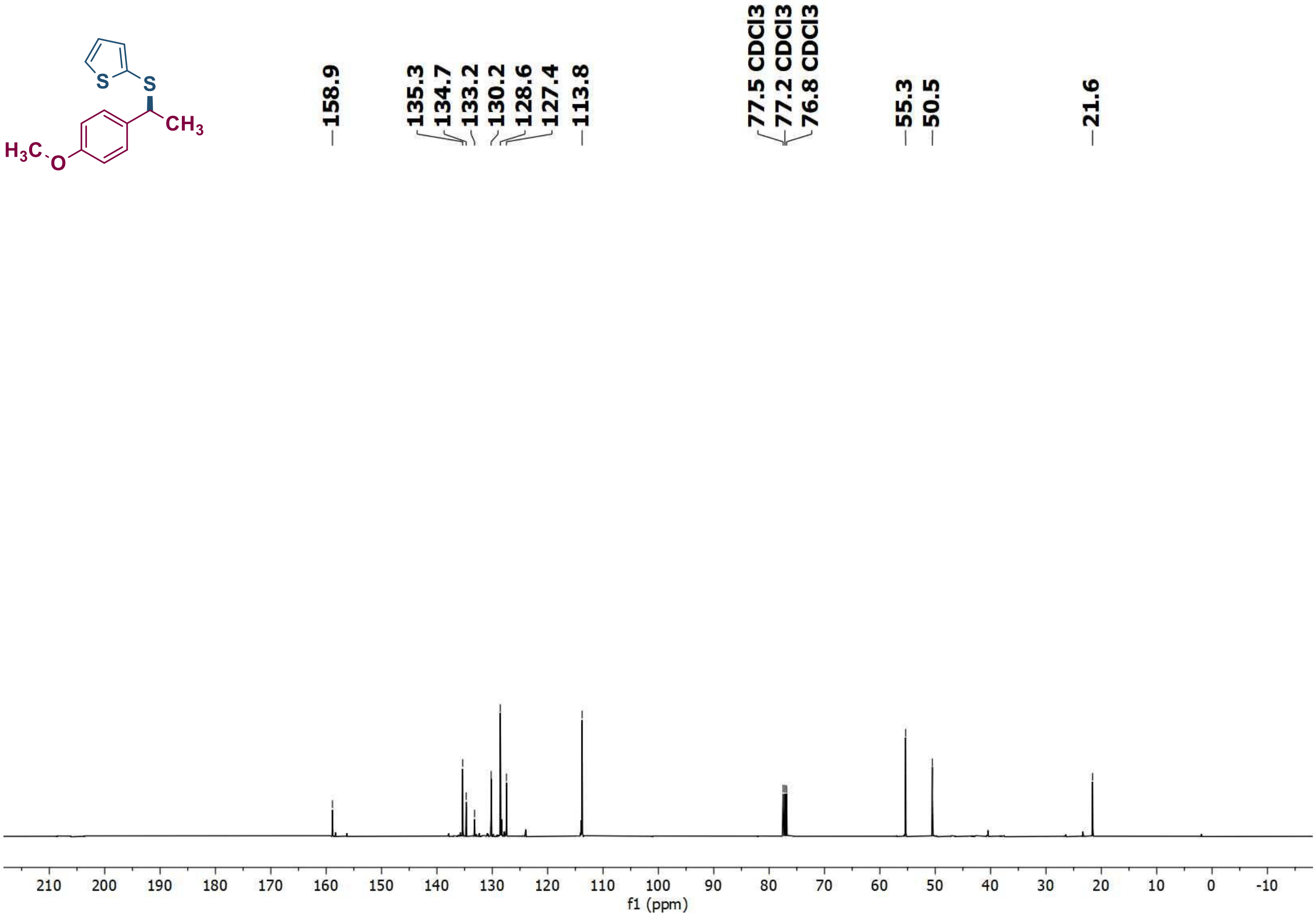

Figure S30. ^{13}C{^{1}H} NMR (101 MHz, Chloroform-d, 25°C) of 2-((1-(4-methoxyphenyl)ethyl)thio)thiophene (**3m**).

Heptyl(1-(4-methoxyphenyl)ethyl)sulfane (**3n**)

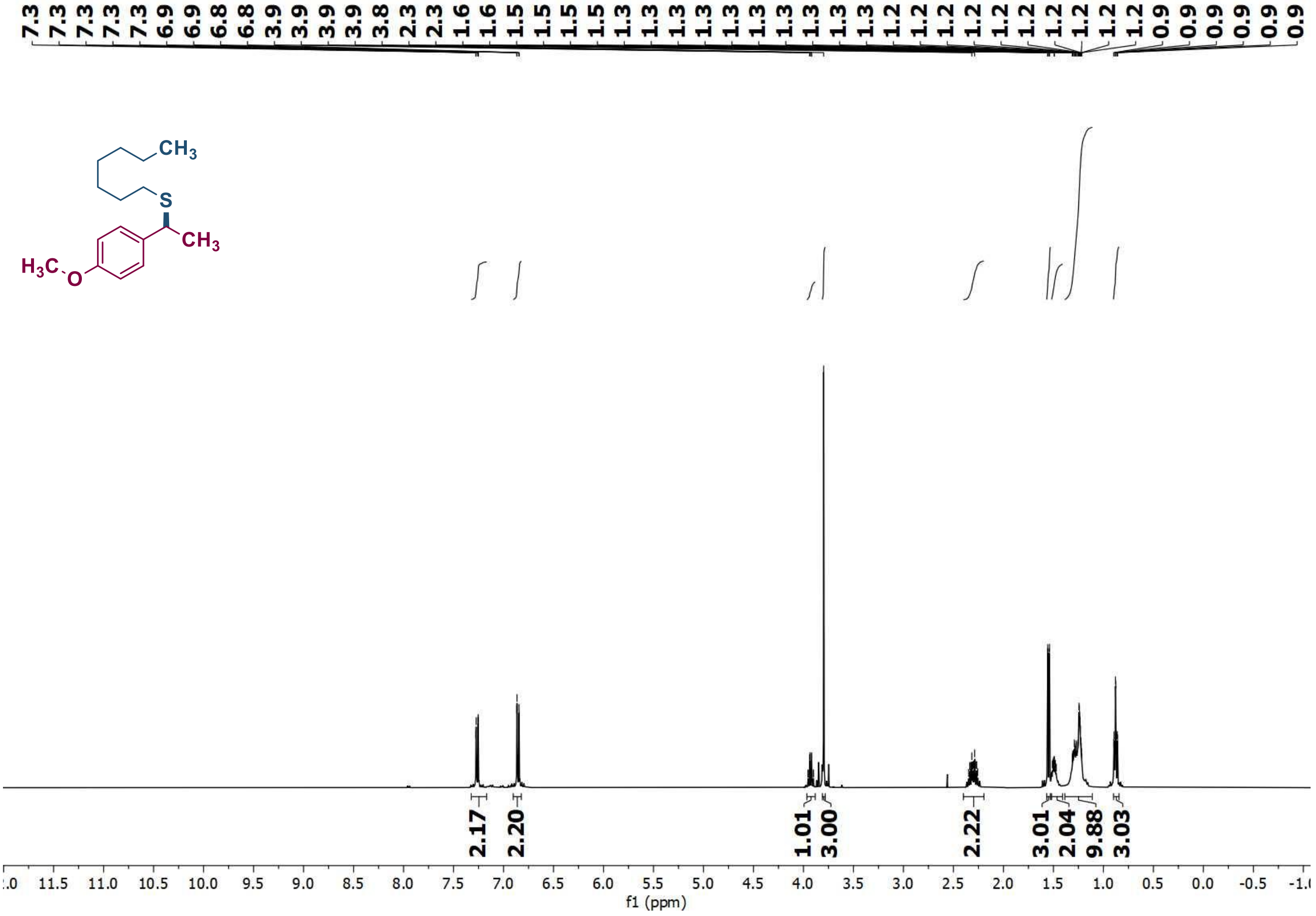

Figure S31. ^{1}H NMR (400 MHz, Chloroform-d, 25°C) of heptyl(1-(4-methoxyphenyl)ethyl)sulfane (**3n**).

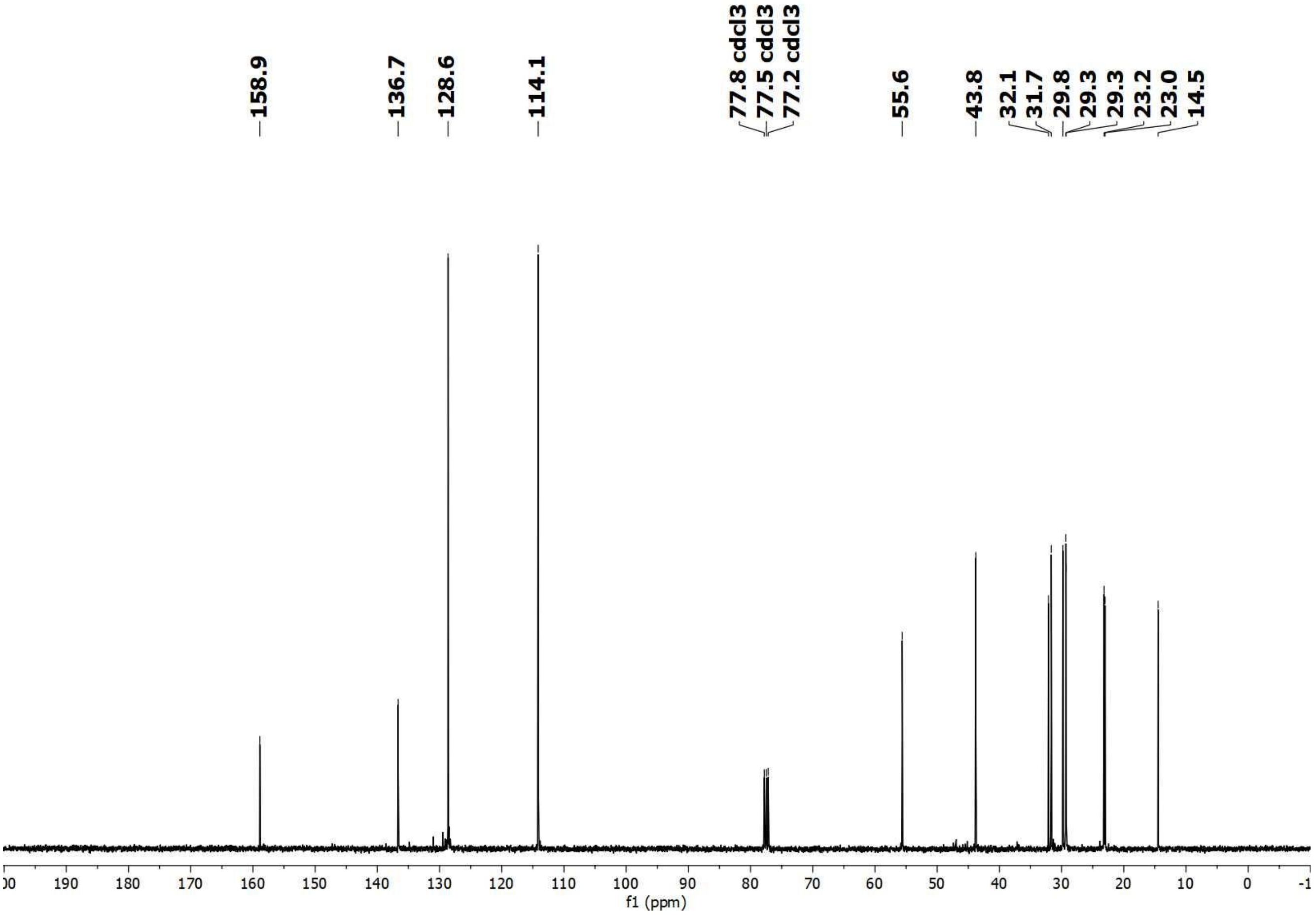

Figure S32. ^{13}C{^{1}H} NMR (101 MHz, Chloroform-d, 25°C) of heptyl(1-(4-methoxyphenyl)ethyl)sulfane (**3n**).

(1-(4-Methoxyphenyl)ethyl)(octyl)sulfane (**3o**)

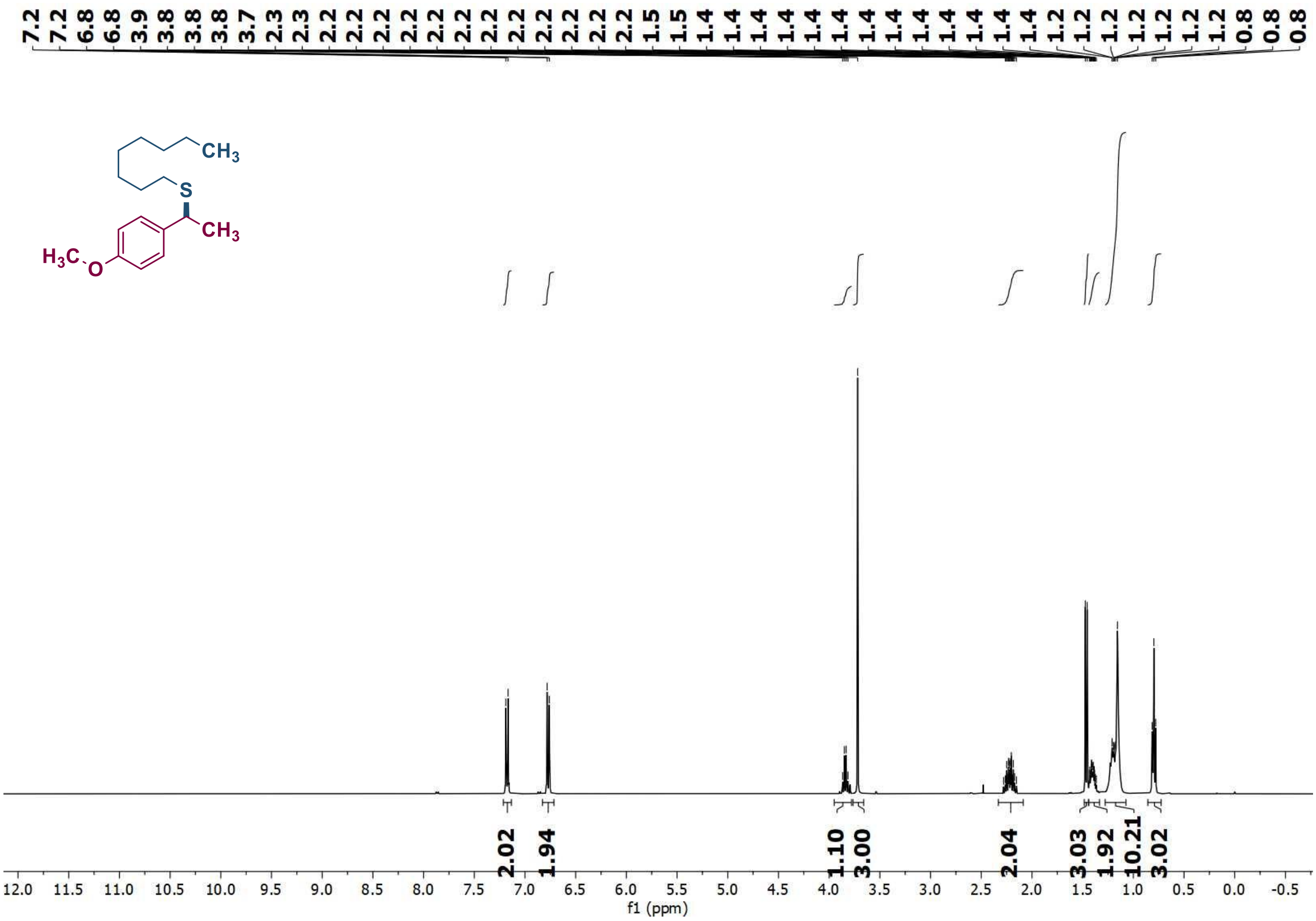

Figure S33. ^{1}H NMR (400 MHz, Chloroform-d, 25°C) of (1-(4-methoxyphenyl)ethyl)(octyl)sulfane (**3o**).

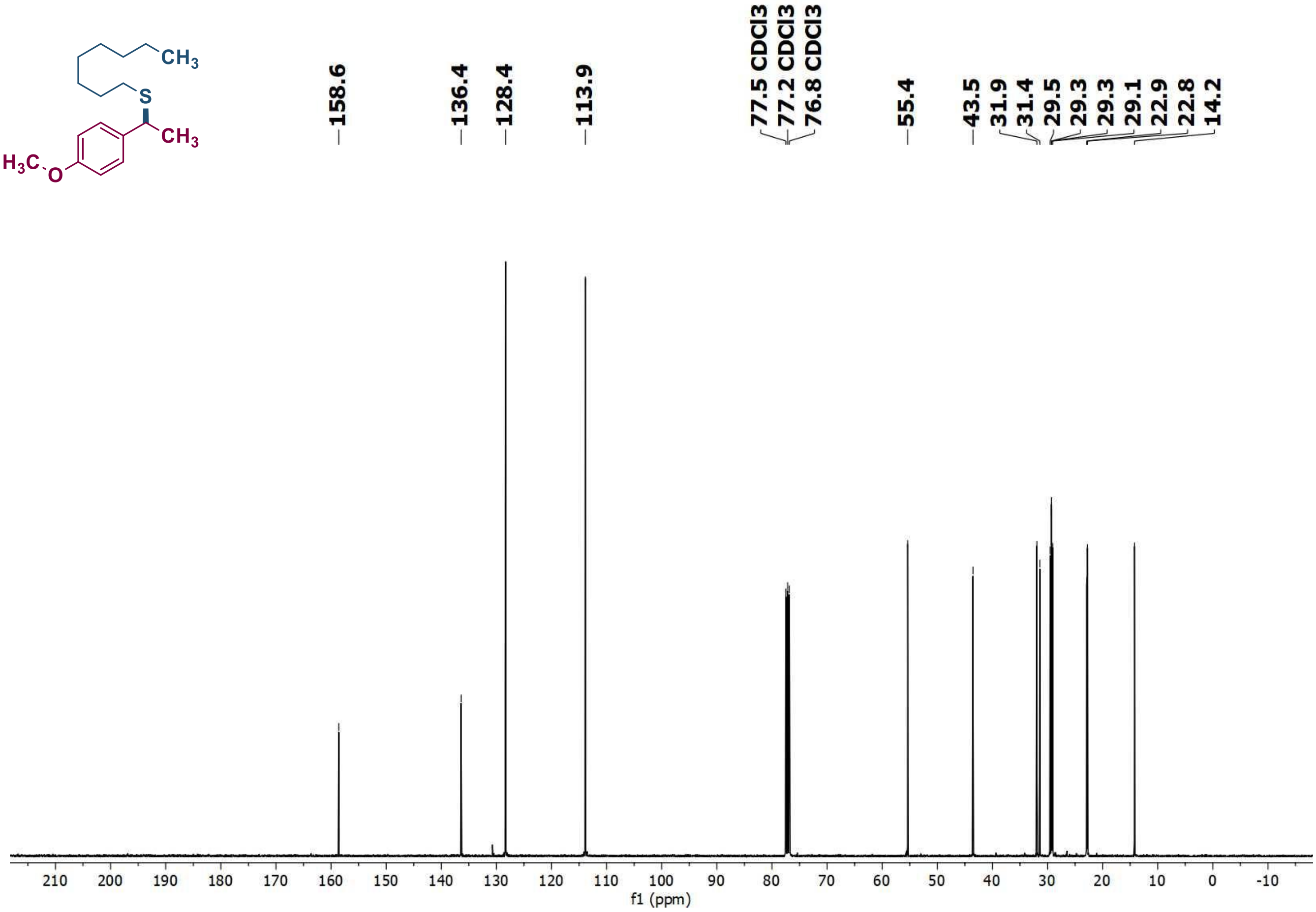

Figure S34. ^{13}C{^{1}H} NMR (101 MHz, Chloroform-d, 25°C) of (1-(4-methoxyphenyl)ethyl)(octyl)sulfane (**3o**).

Cyclohexyl(1-(4-methoxyphenyl)ethyl)sulfane (**3p**)

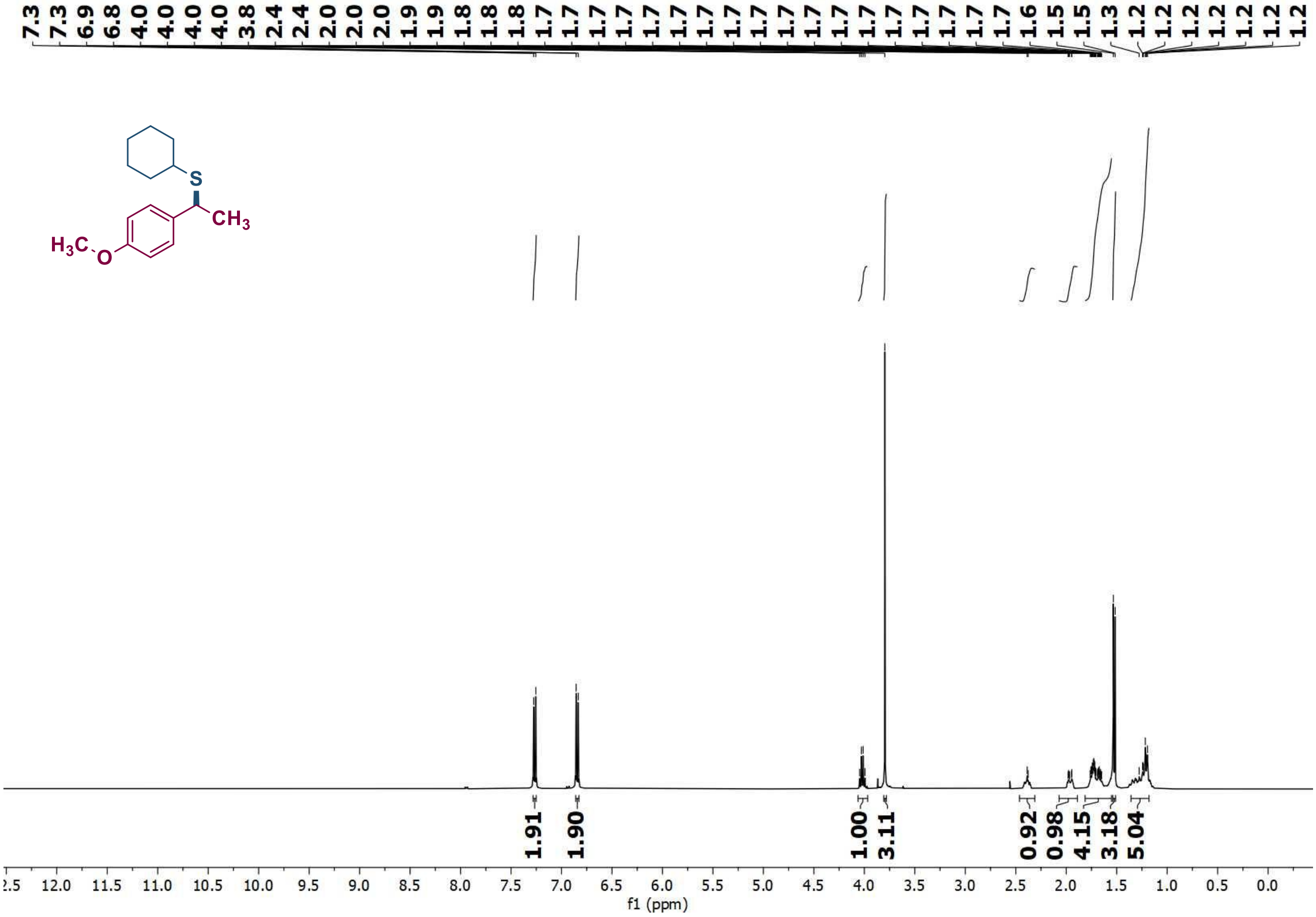

Figure S35. ^{1}H NMR (400 MHz, Chloroform-d, 25°C) of cyclohexyl(1-(4-methoxyphenyl)ethyl)sulfane (**3p**).

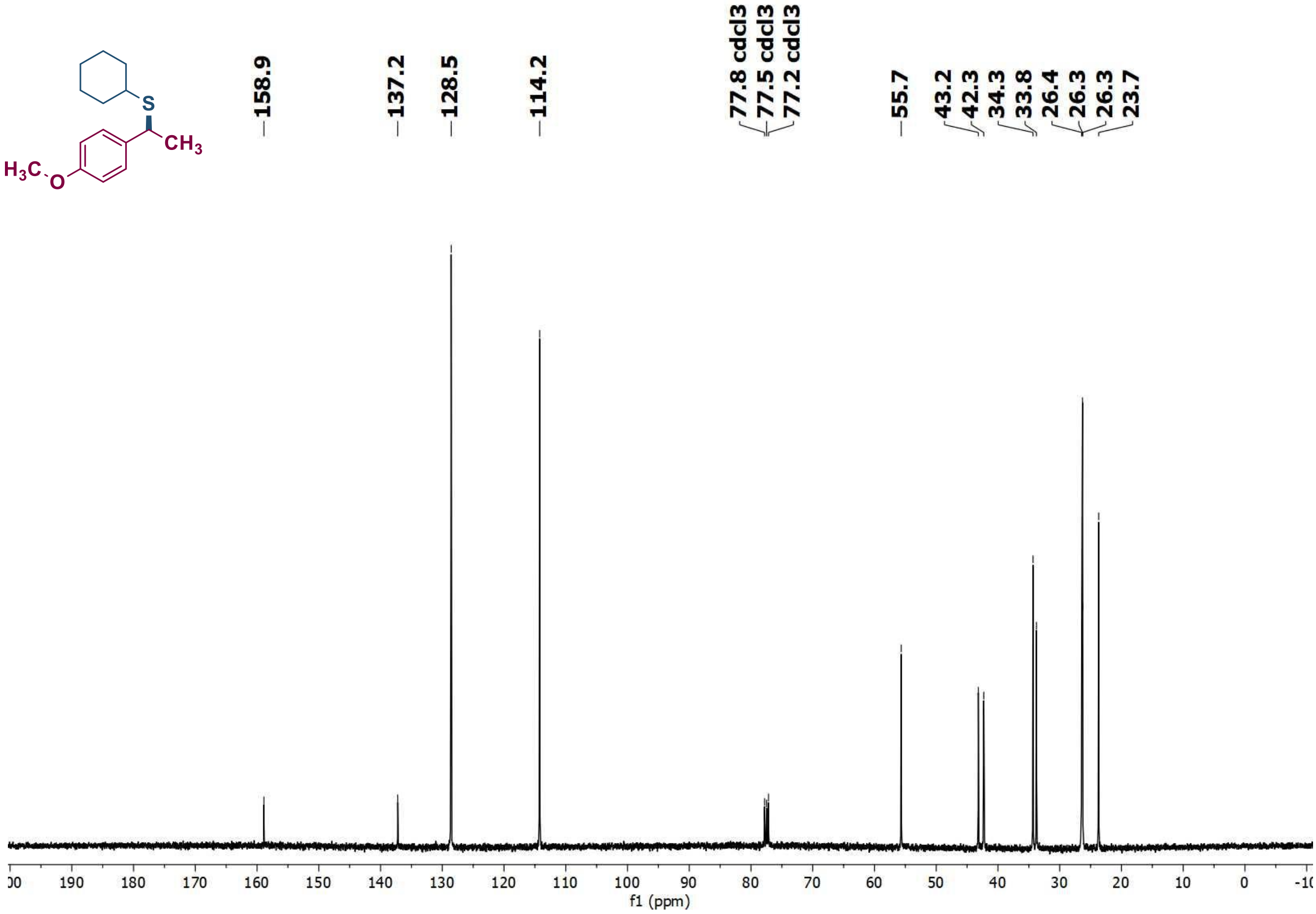

Figure S36. $^{13}C\{^{1}H\}$ NMR (101 MHz, Chloroform-d, 25°C) of cyclohexyl(1-(4-methoxyphenyl)ethyl)sulfane (**3p**).

3-((1-(4-Methoxyphenyl)ethyl)thio)propanoic acid (**3q**)

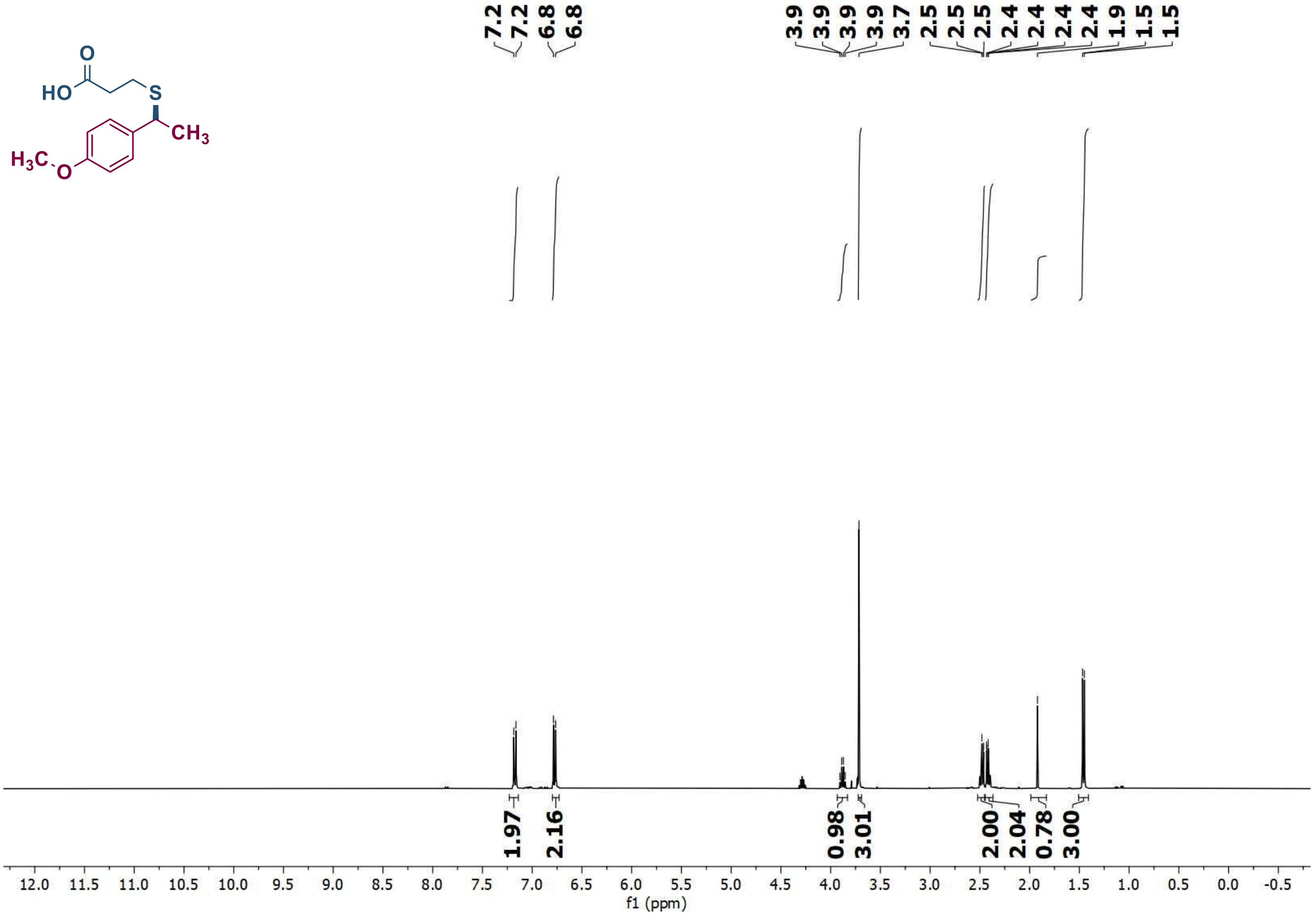

Figure S37. ^{1}H NMR (400 MHz, Chloroform-d, 25°C) of 3-((1-(4-methoxyphenyl)ethyl)thio)propanoic acid (**3q**).

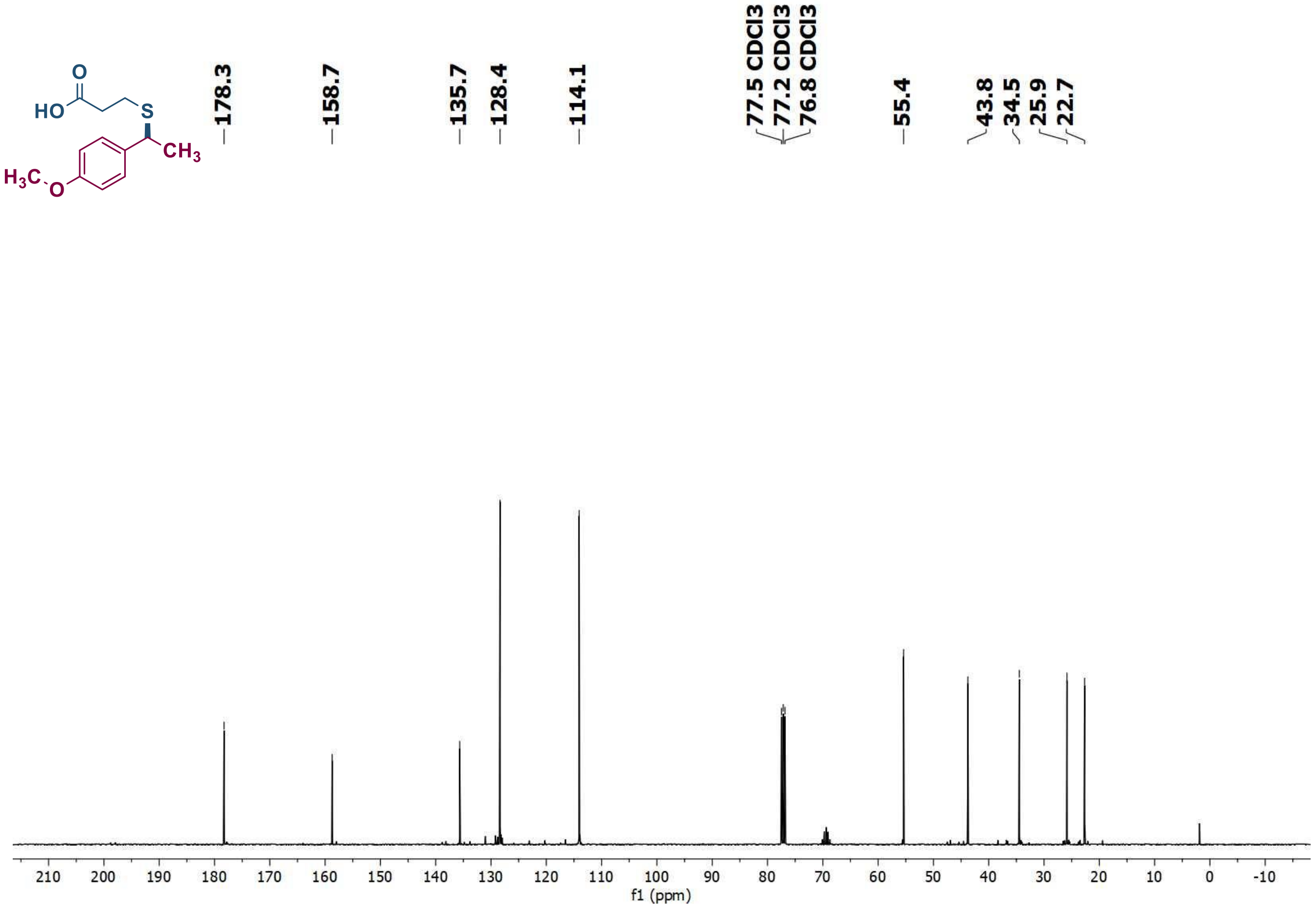

Figure S38. ^{13}C{^{1}H} NMR (101 MHz, Chloroform-d, 25°C) of 3-((1-(4-methoxyphenyl)ethyl)thio)propanoic acid (**3q**).

Methyl 2-((1-(4-methoxyphenyl)ethyl)thio)acetate (**3r**)

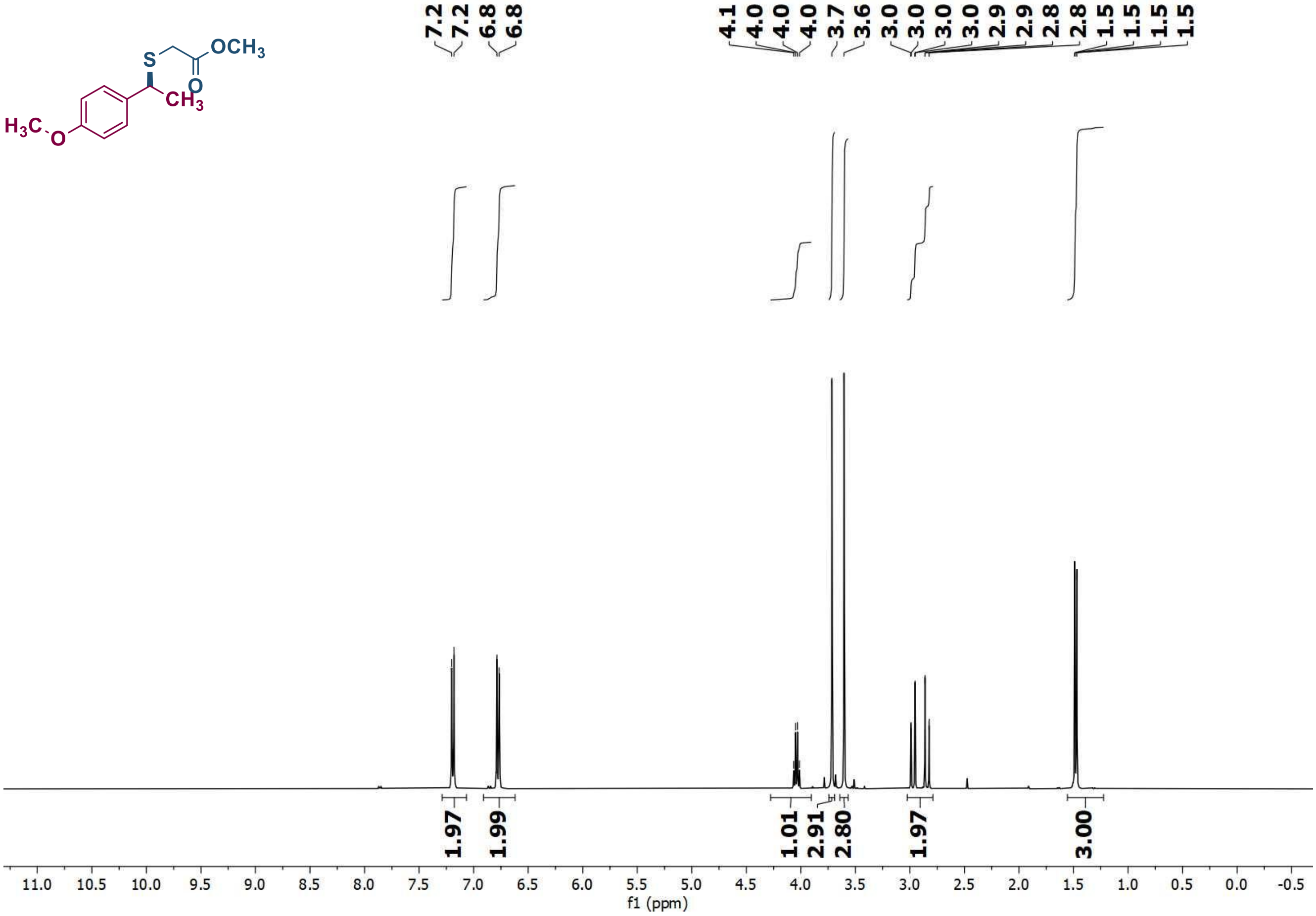

Figure S39. ^{1}H NMR (400 MHz, Chloroform-d, 25°C) of methyl 2-((1-(4-methoxyphenyl)ethyl)thio)acetate (**3r**).

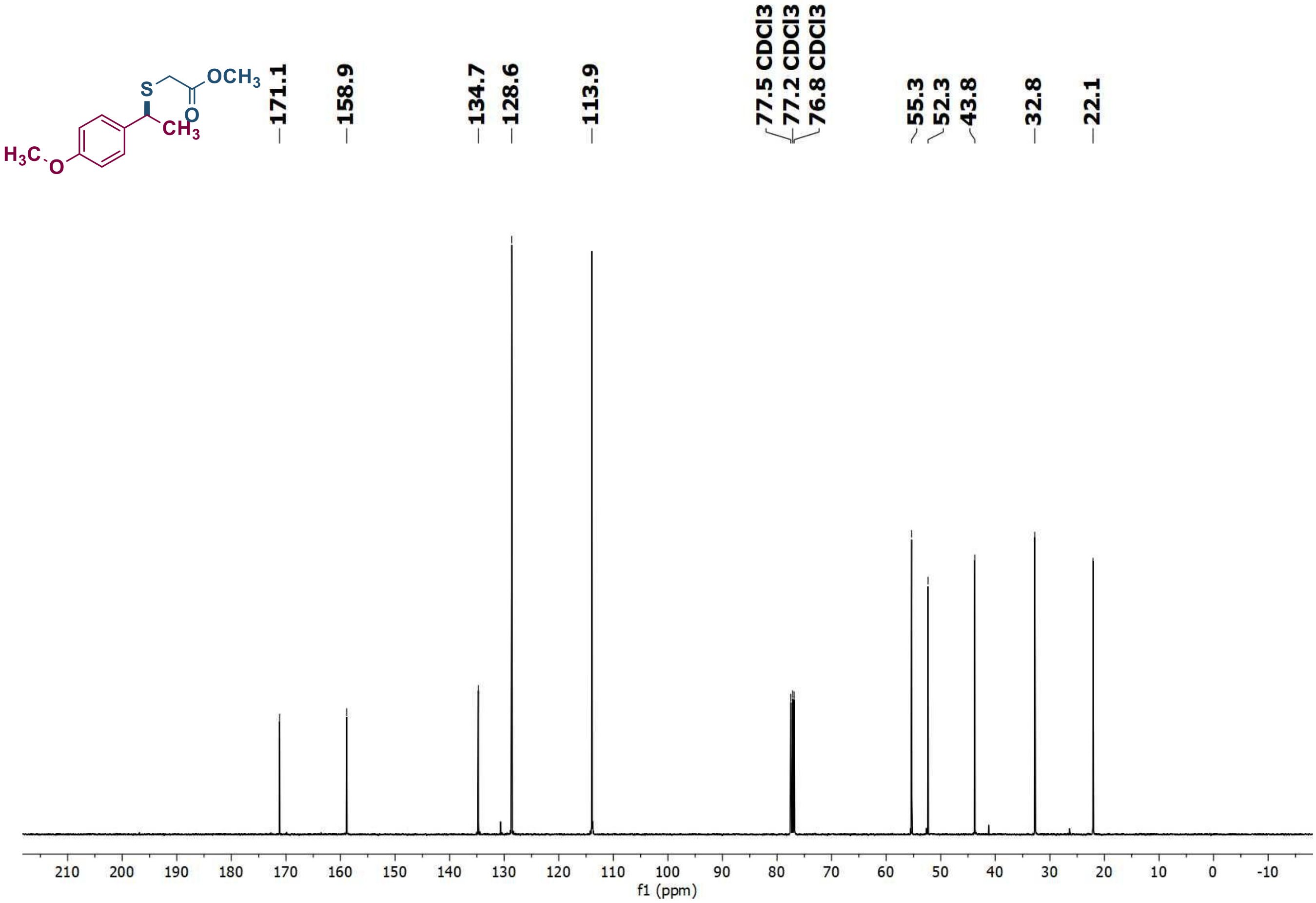

Figure S40. ^{13}C{^{1}H} NMR (101 MHz, Chloroform-d, 25°C) of methyl 2-((1-(4-methoxyphenyl)ethyl)thio)acetate (**3r**).

(4-Chlorobenzyl)(1-(4-methoxyphenyl)ethyl)sulfane (**3s**)

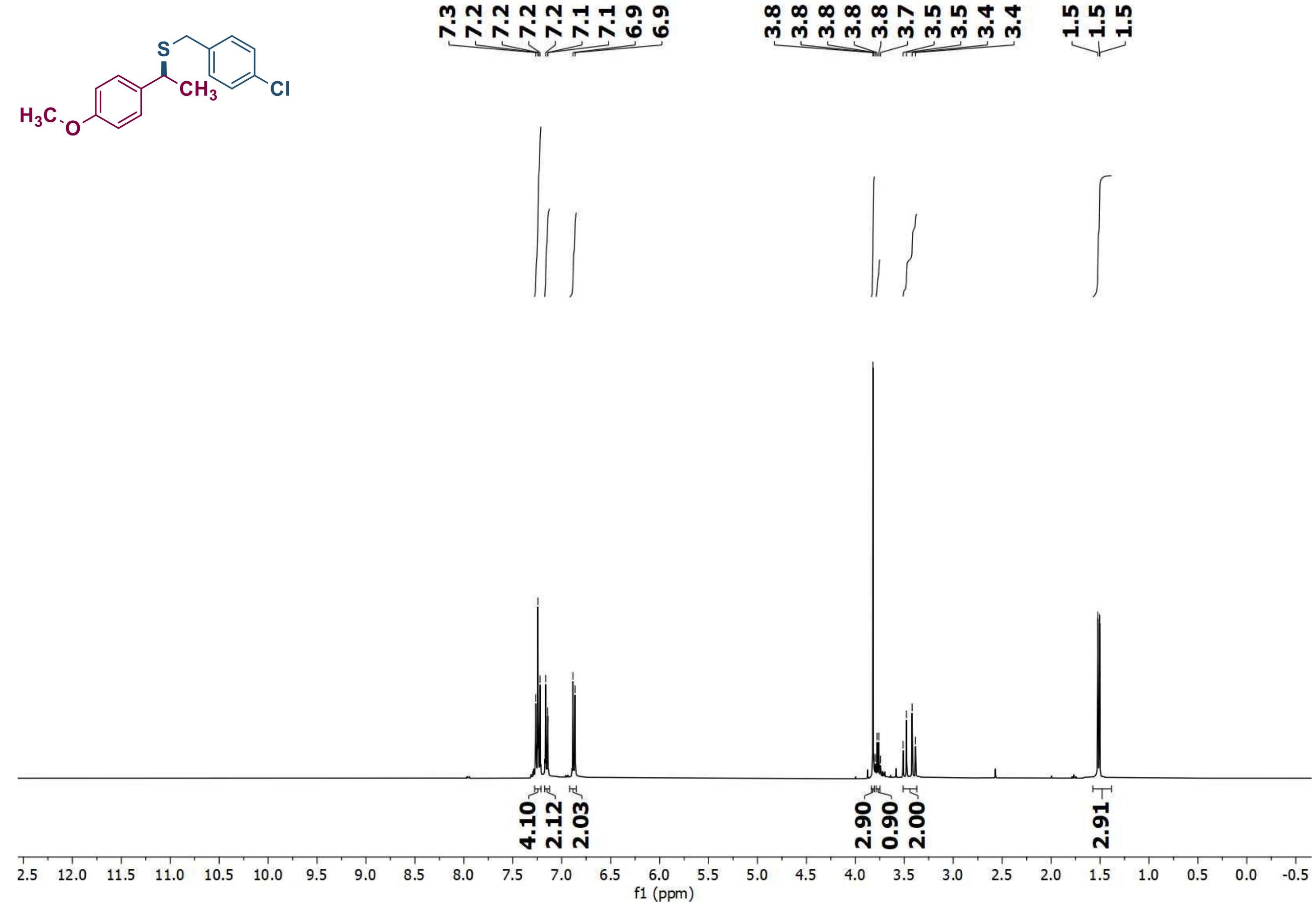

Figure S41. ^{1}H NMR (400 MHz, Chloroform-d, 25°C) of (4-chlorobenzyl)(1-(4-methoxyphenyl)ethyl)sulfane (**3s**).

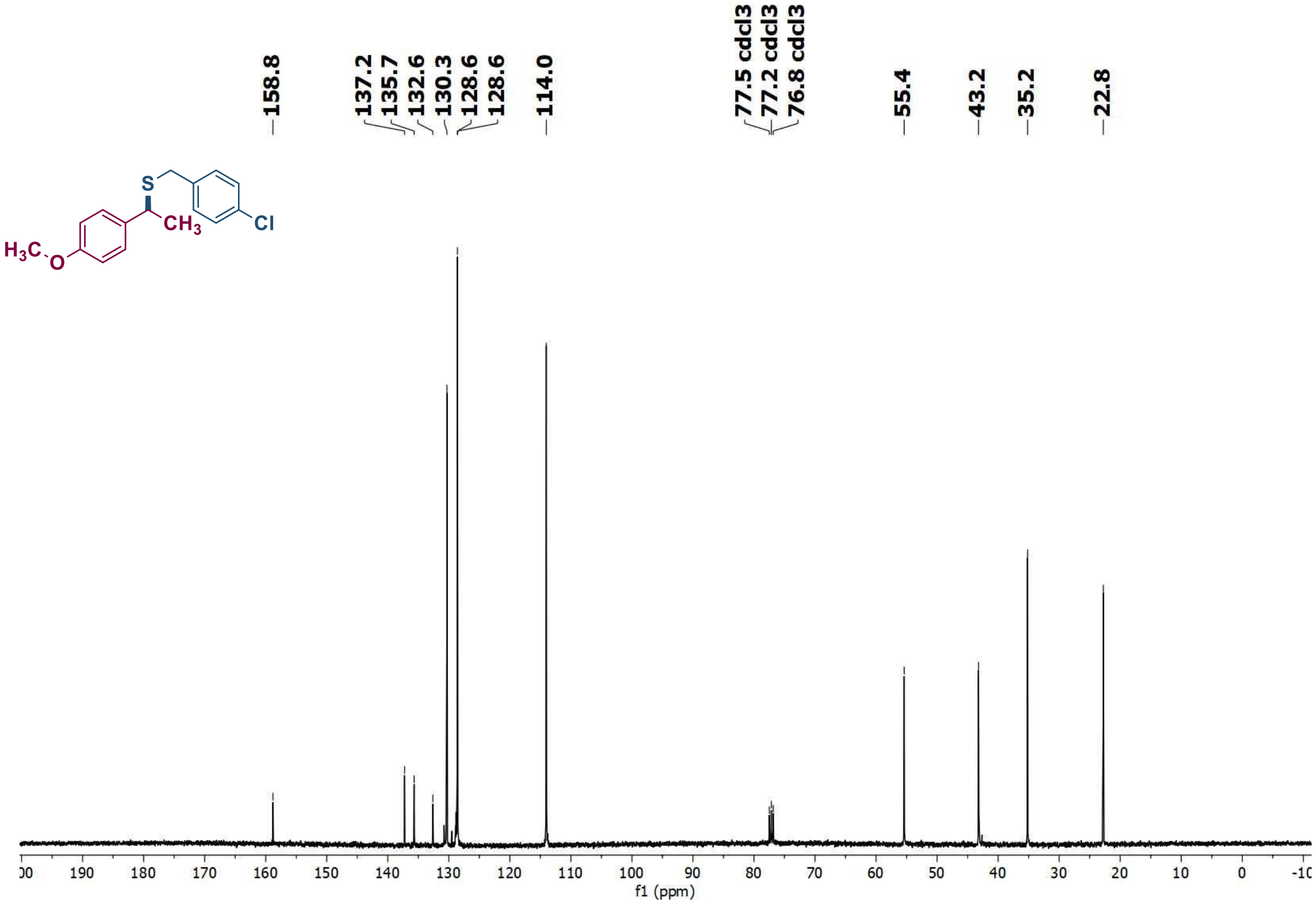

Figure S42. $^{13}C\{^{1}H\}$ NMR (101 MHz, Chloroform-d, 25°C) of (4-chlorobenzyl)(1-(4-methoxyphenyl)ethyl)sulfane (**3s**).

1-(1-(4-Methoxyphenyl)ethyl)thio)-10-(1-(4-methoxyphenyl)ethyl)thio)decane (**3t**)

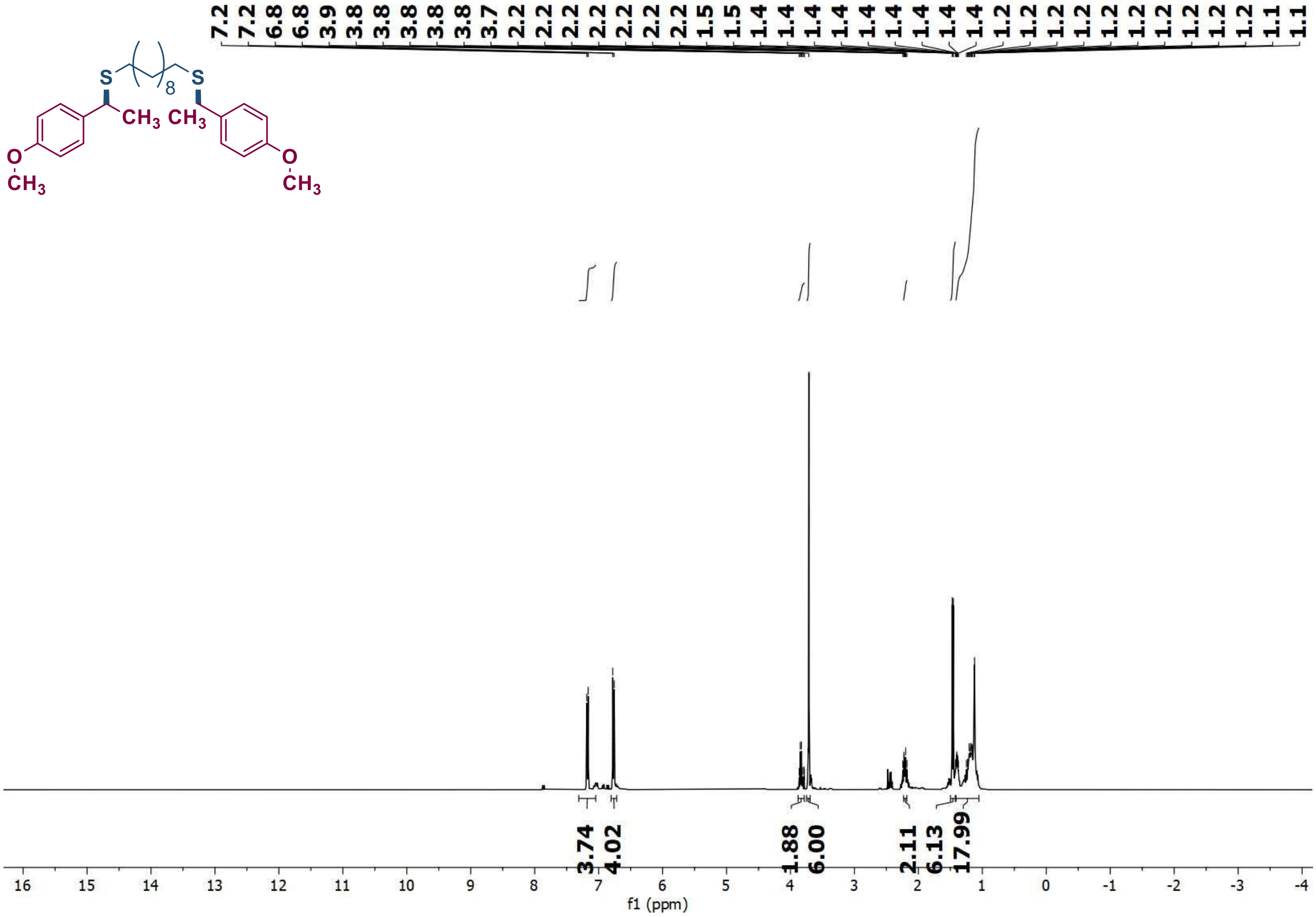

Figure S43. ^{1}H NMR (400 MHz, Chloroform-d, 25°C) of 1-(1-(4-methoxyphenyl)ethyl)thio)-10-(1-(4-methoxyphenyl)ethyl)thio)decane (**3t**).

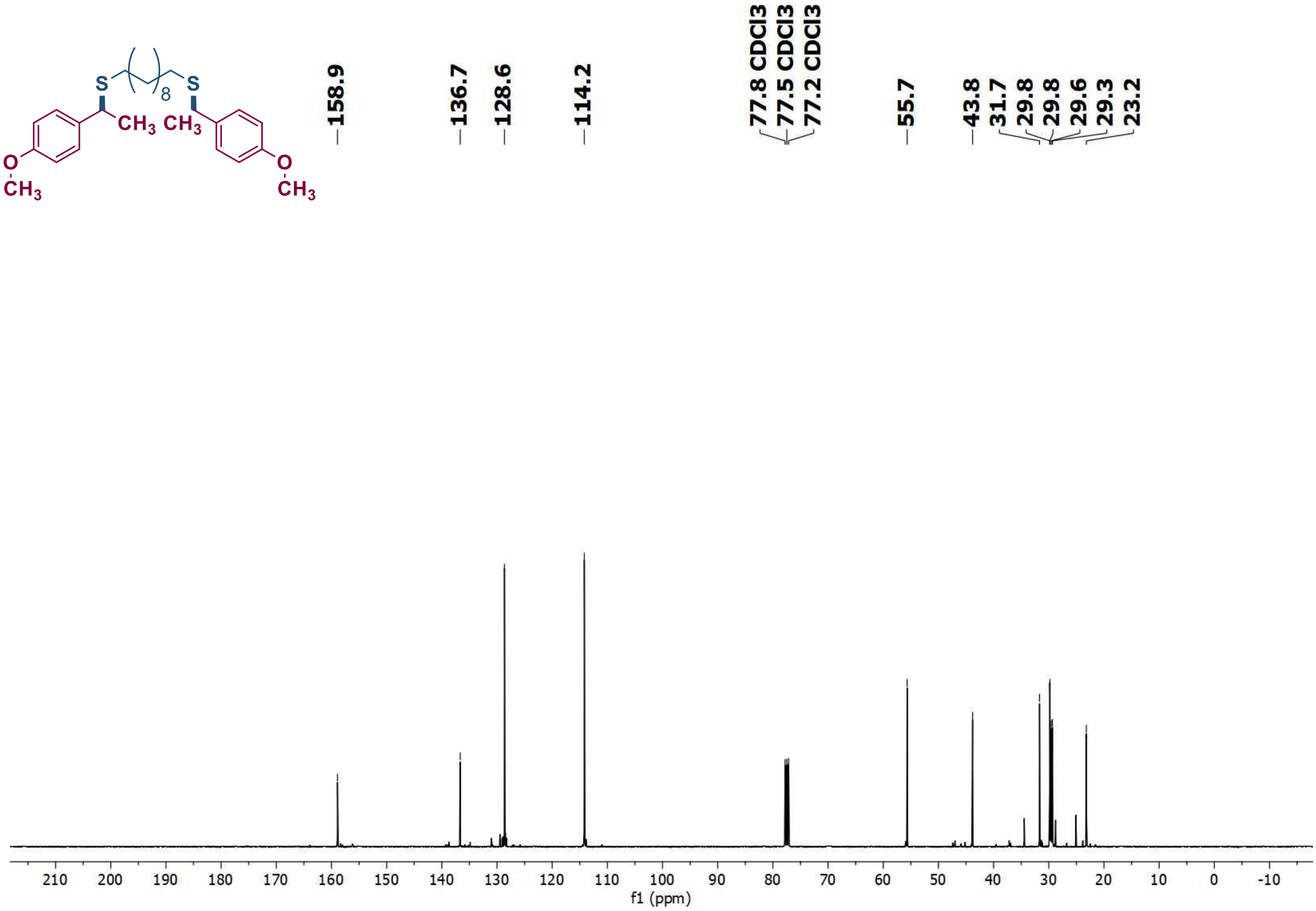

Figure S44. ^{13}C{^{1}H} NMR (101 MHz, Chloroform-d, 25°C) of 1-(1-(4-methoxyphenyl)ethyl)thio)-10-(1-(4-methoxyphenyl)ethyl)thio)decane (**3t**).

Phenyl(1,2,3,4-tetrahydronaphthalen-1-yl)sulfane (**4a**)

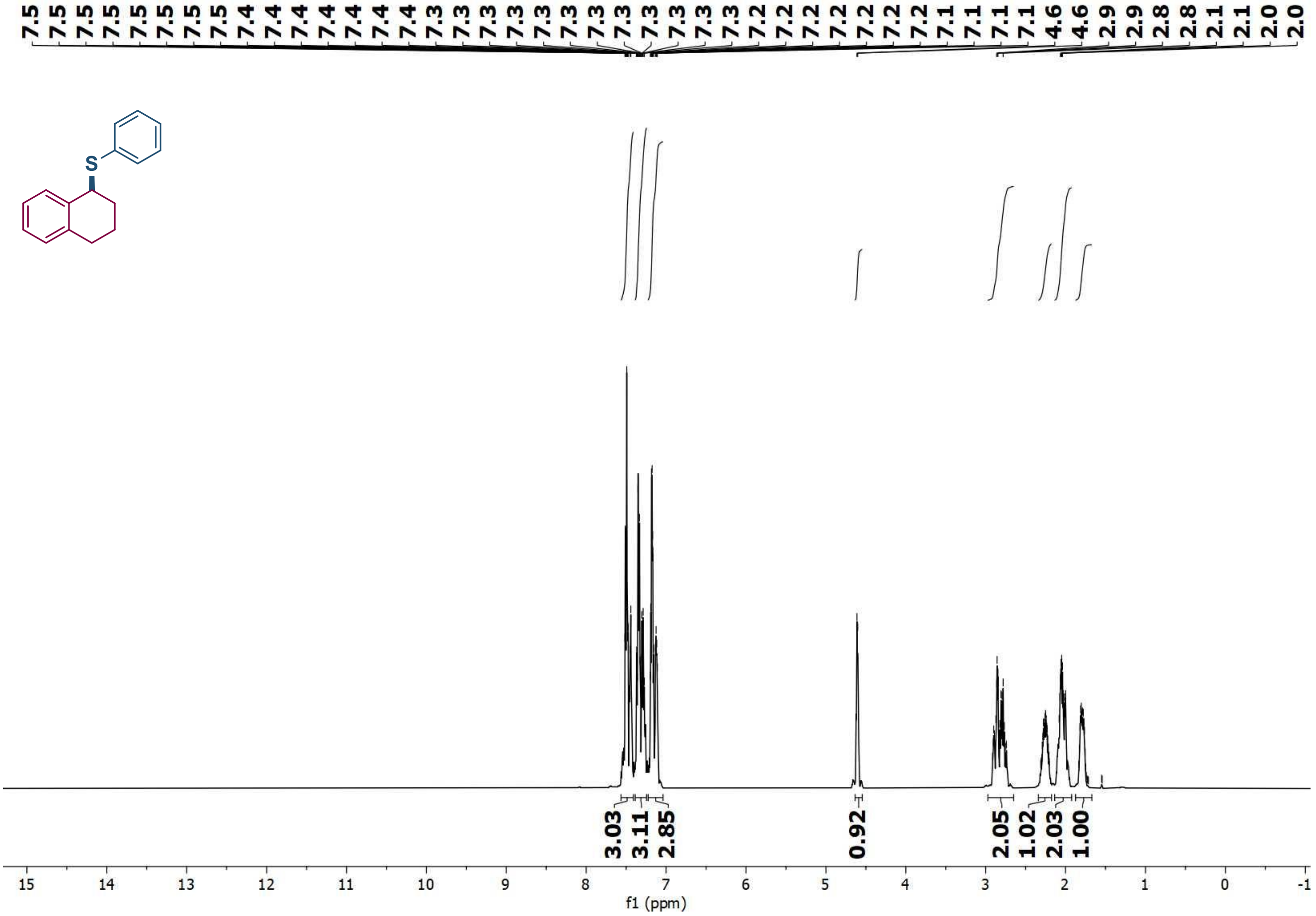

Figure S45. ^{1}H NMR (400 MHz, Chloroform-d, 25°C) of phenyl(1,2,3,4-tetrahydronaphthalen-1-yl)sulfane (**4a**).

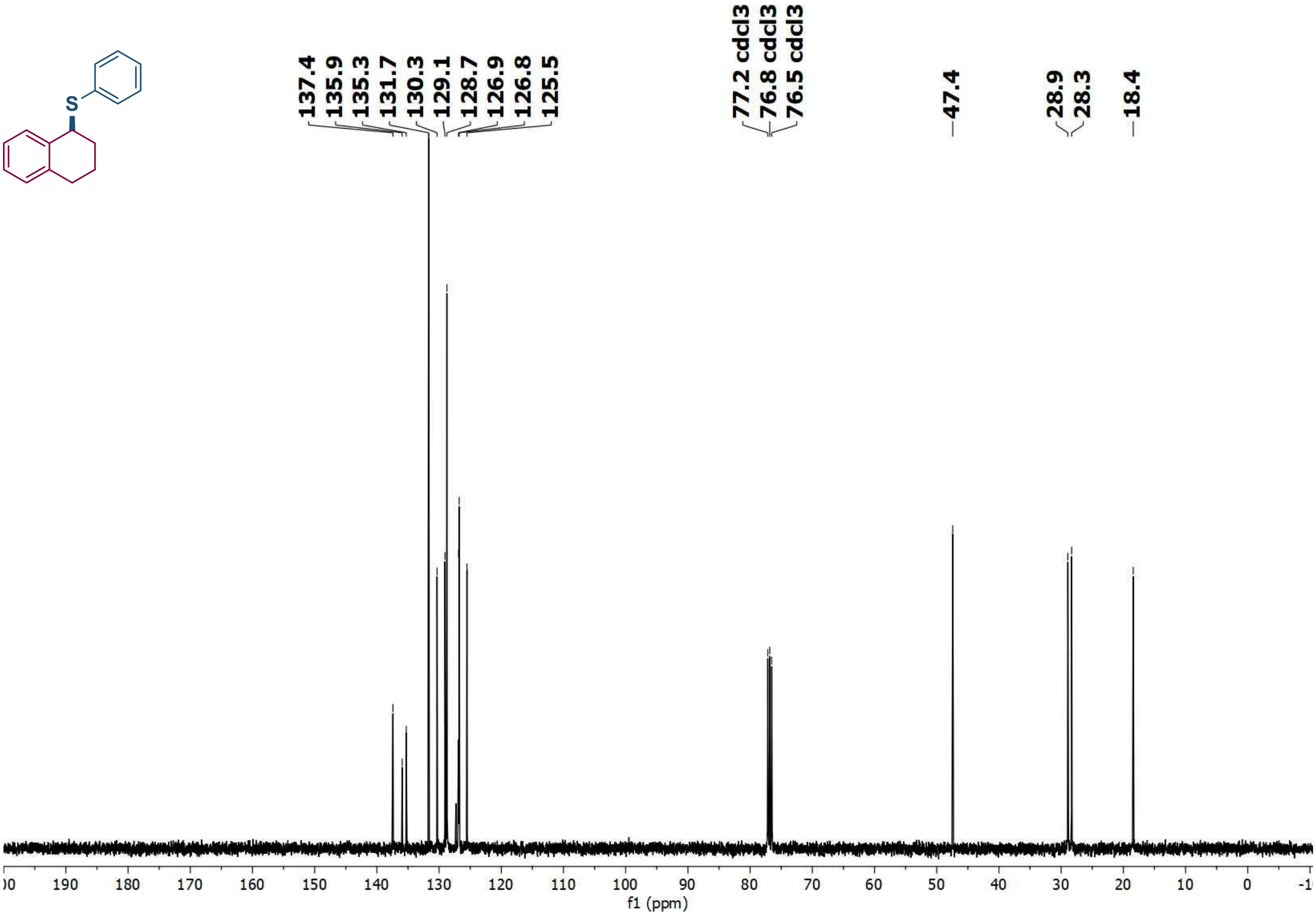

Figure S46. ^{13}C{^{1}H} NMR (101 MHz, Chloroform-d, 25°C) of phenyl(1,2,3,4-tetrahydronaphthalen-1-yl)sulfane (**4a**).

(Cyclopropyl(phenyl)methyl)(phenyl)sulfane (**4b**)

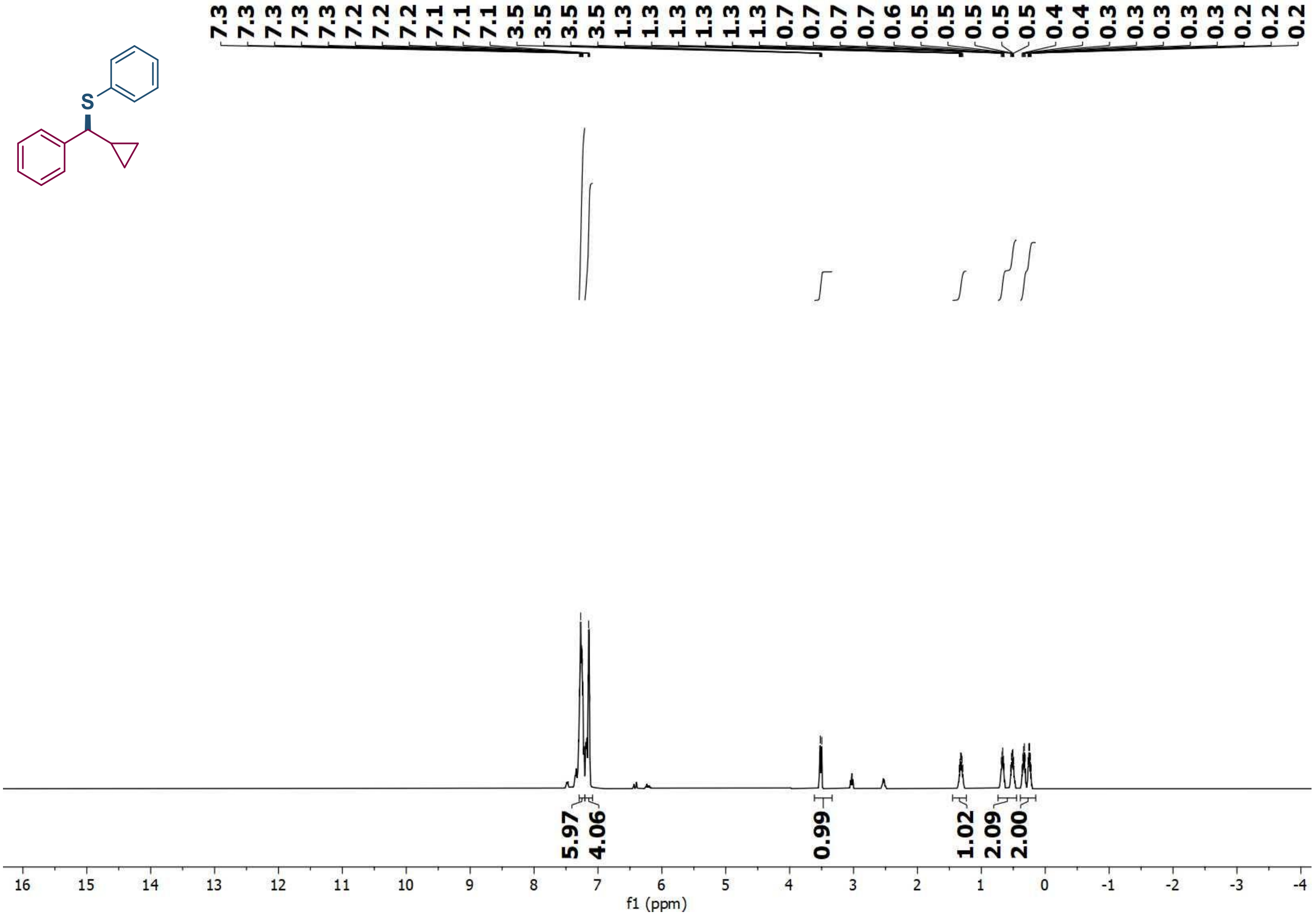

Figure S47. ^{1}H NMR (400 MHz, Chloroform-d, 25°C) of (cyclopropyl(phenyl)methyl)(phenyl)sulfane (**4b**).

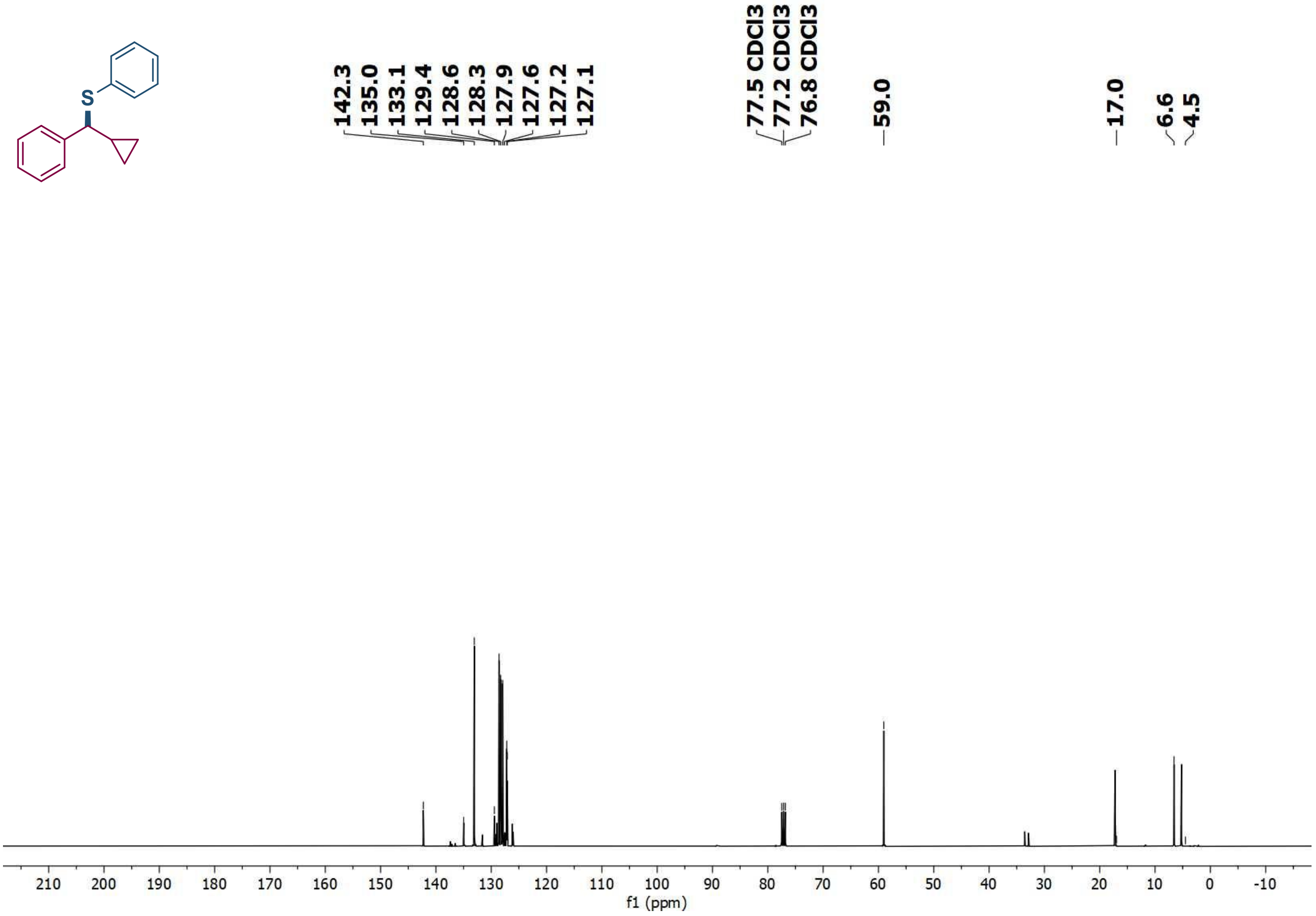

Figure S48. $^{13}C\{^{1}H\}$ NMR (101 MHz, Chloroform-d, 25°C) of (cyclopropyl(phenyl)methyl)(phenyl)sulfane (**4b**).

(2,3-Dihydro-1H-inden-1-yl)(phenyl)sulfane (**4c**)

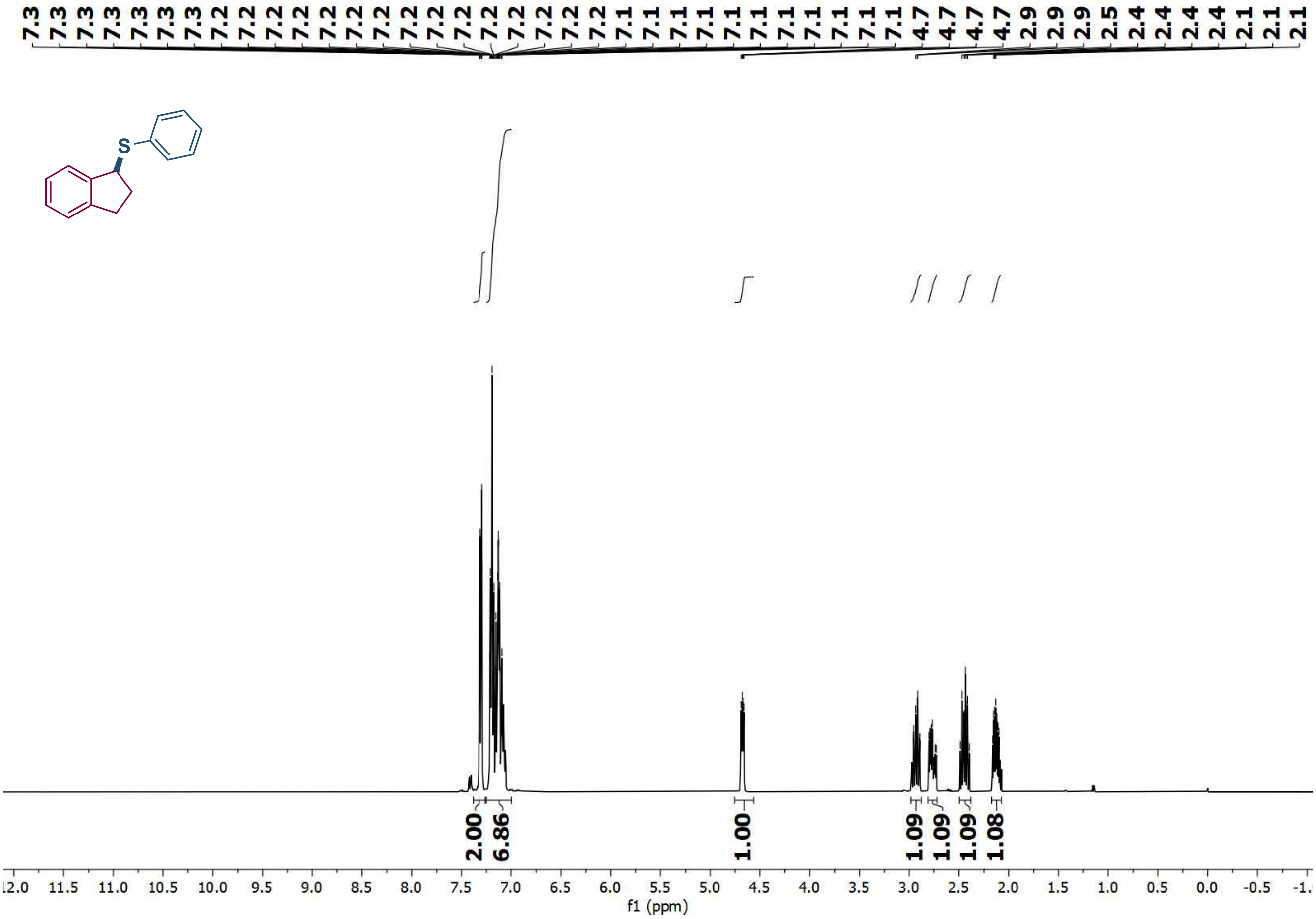

Figure S49. ^{1}H NMR (400 MHz, Chloroform-d, 25°C) of (2,3-dihydro-1H-inden-1-yl)(phenyl)sulfane (**4c**).

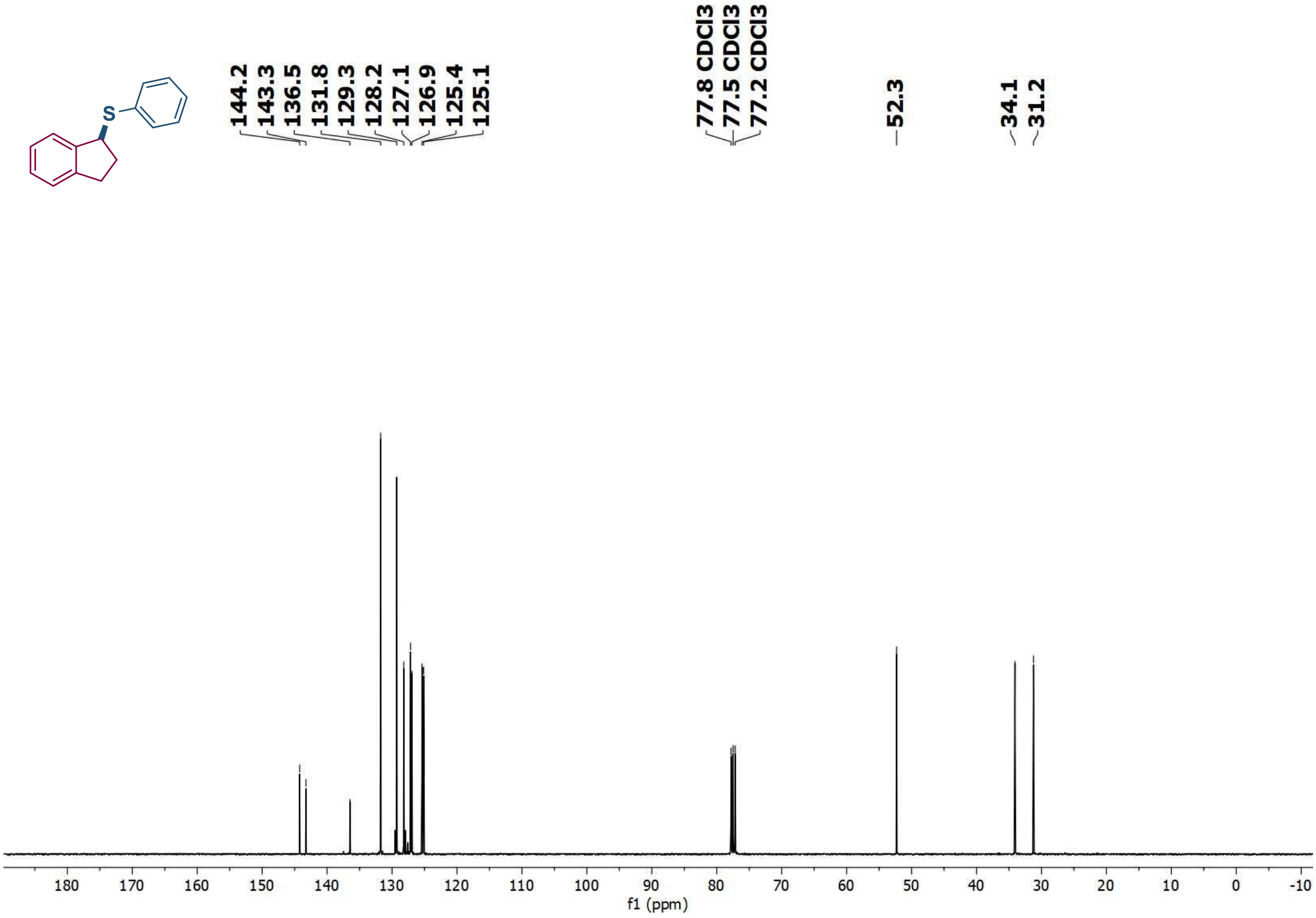

Figure S50. ^{13}C{^{1}H} NMR (101 MHz, Chloroform-d, 25°C) of (2,3-dihydro-1H-inden-1-yl)(phenyl)sulfane (**4c**).

Phenyl(1-phenylethyl)sulfane (**4d**)

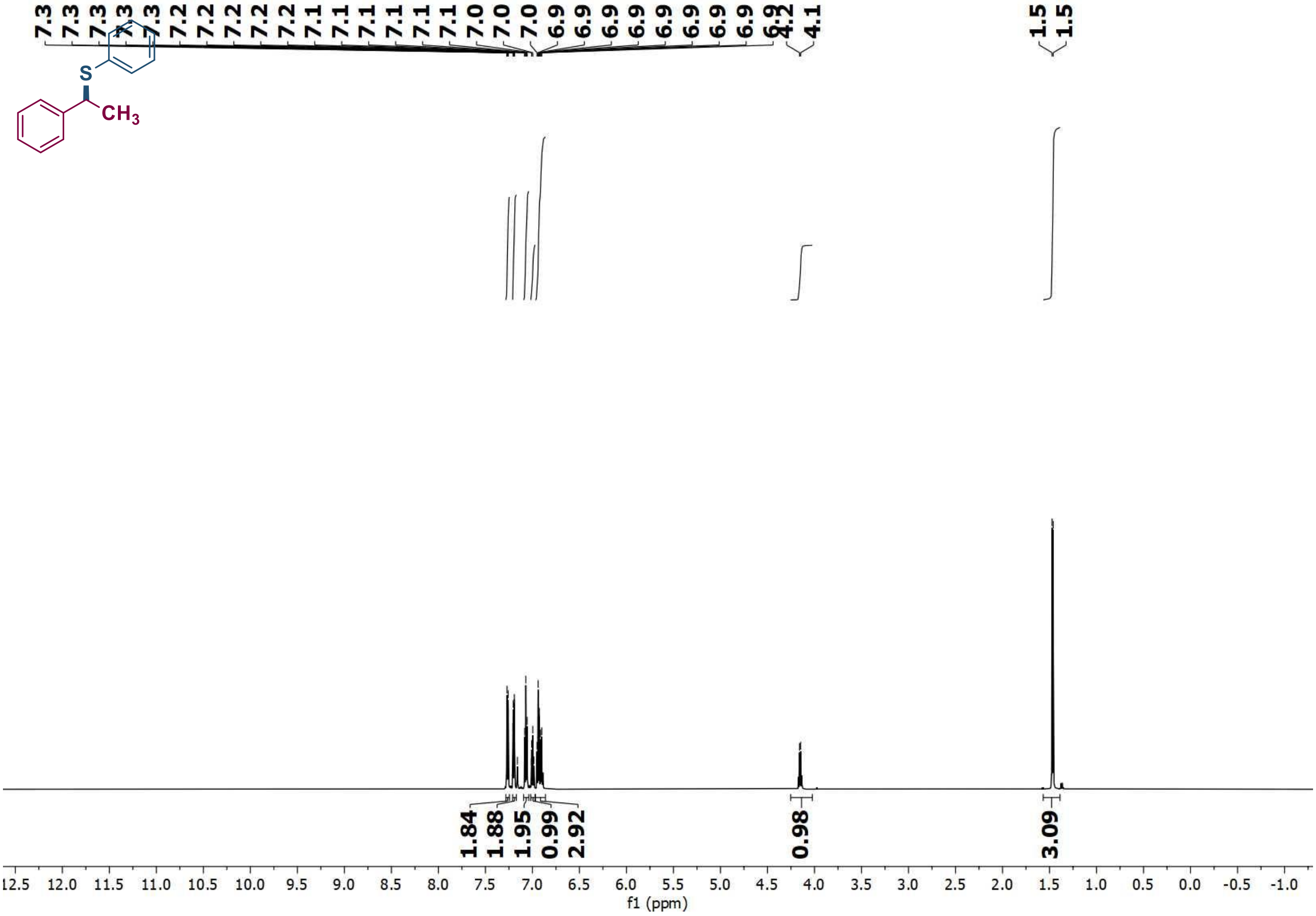

Figure S51. ^{1}H NMR (400 MHz, benzene-d$_6$, 25°C) of phenyl(1-phenylethyl)sulfane (**4d**).

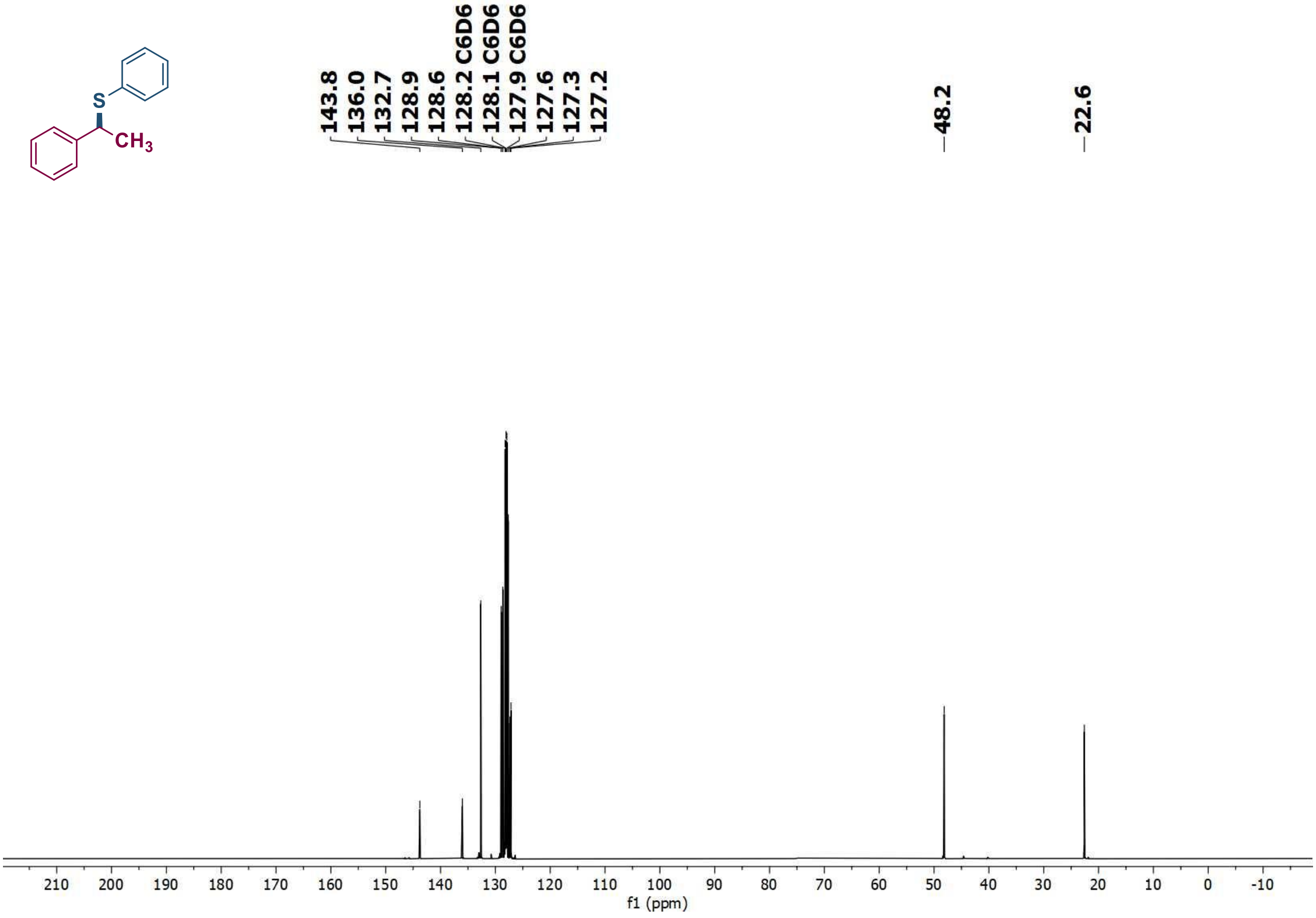

Figure S52. $^{13}C\{^{1}H\}$ NMR (101 MHz, benzene-d$_6$, 25°C) of phenyl(1-phenylethyl)sulfane (**4d**).

(1-(4-Fluorophenyl)ethyl)(phenyl)sulfane (**4e**)

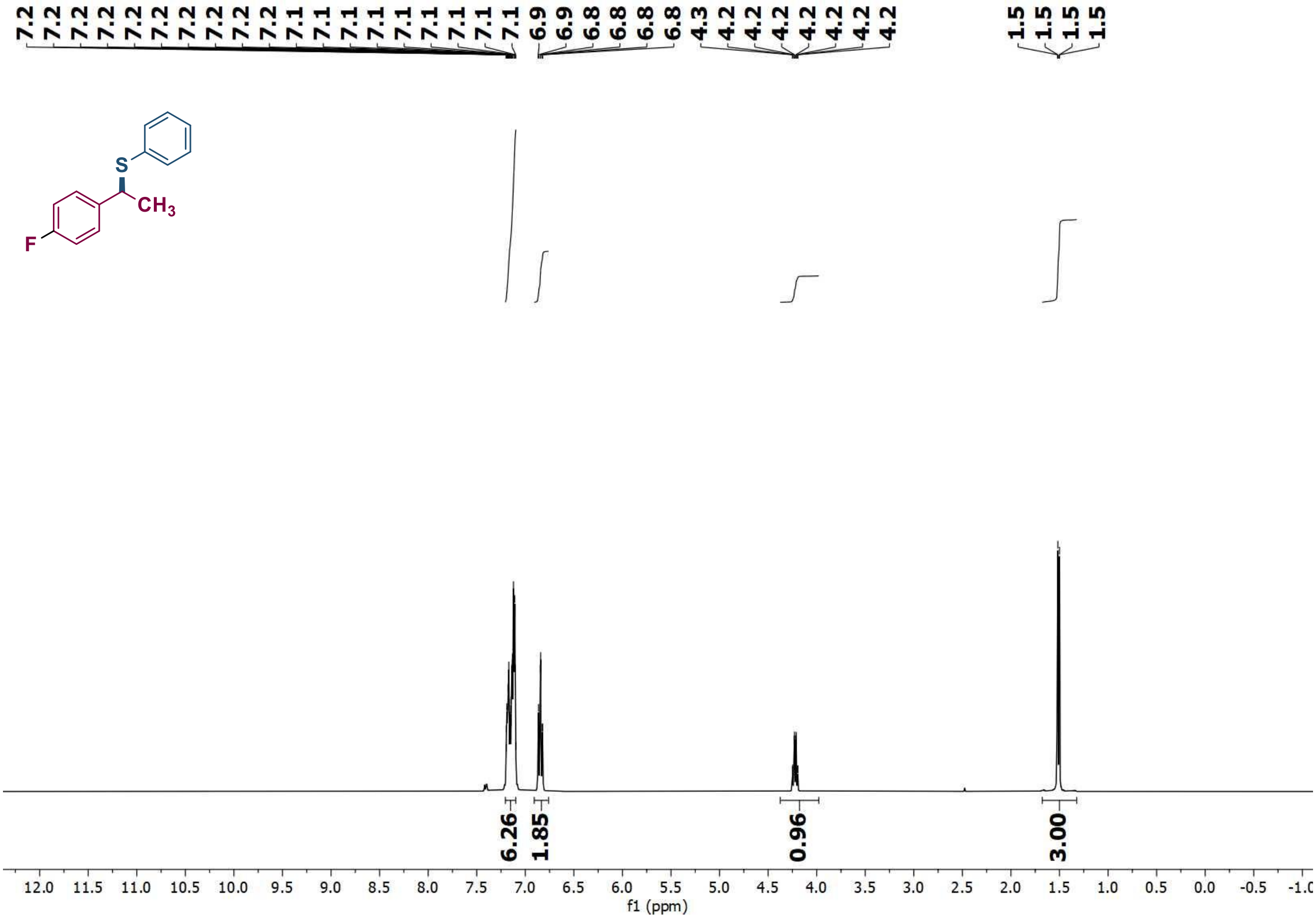

Figure S53. ^{1}H NMR (400 MHz, Chloroform-d, 25°C) of (1-(4-fluorophenyl)ethyl)(phenyl)sulfane (**4e**).

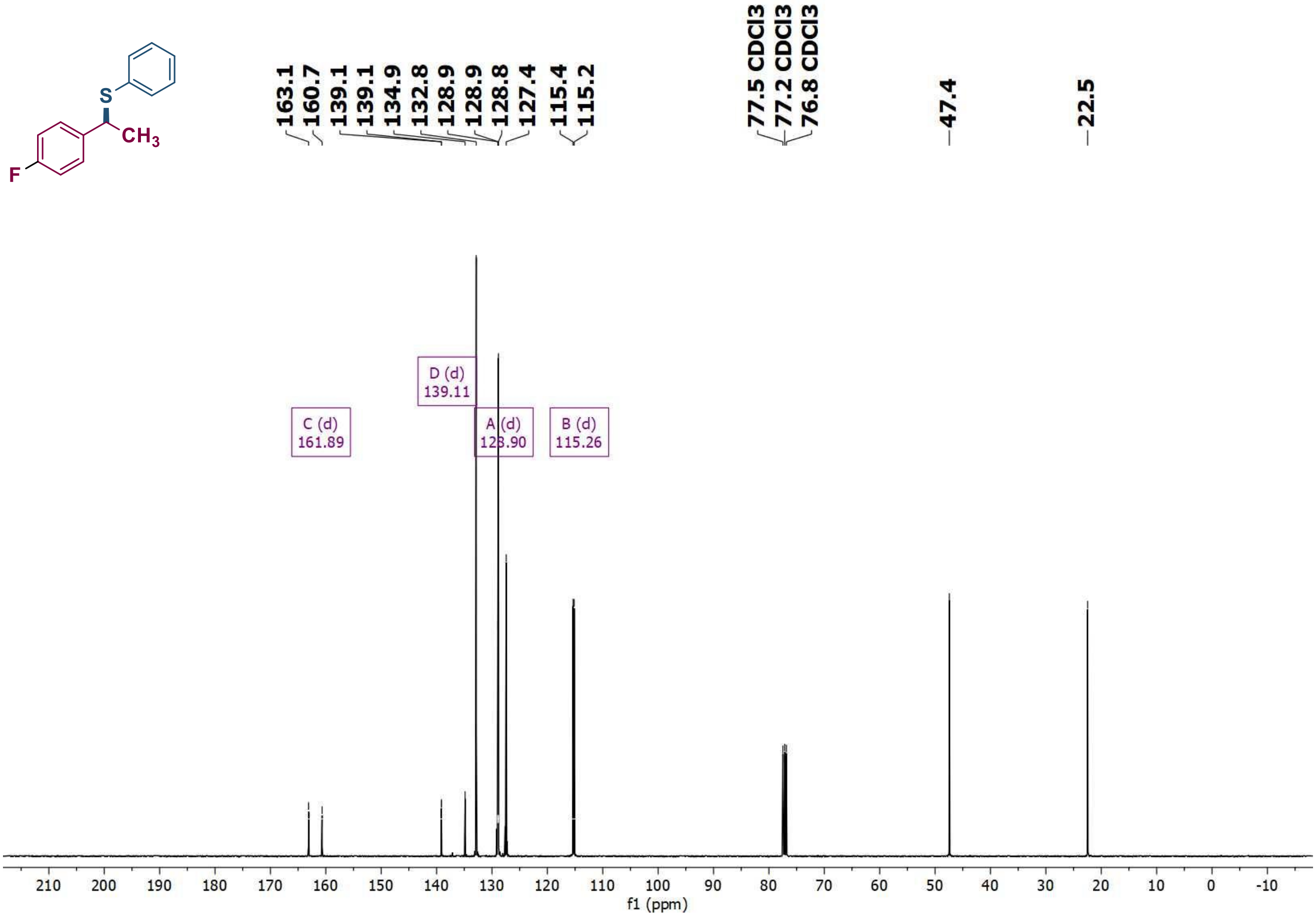

Figure S54. ^{13}C{^{1}H} NMR (101 MHz, Chloroform-d, 25°C) of (1-(4-fluorophenyl)ethyl)(phenyl)sulfane (**4e**).

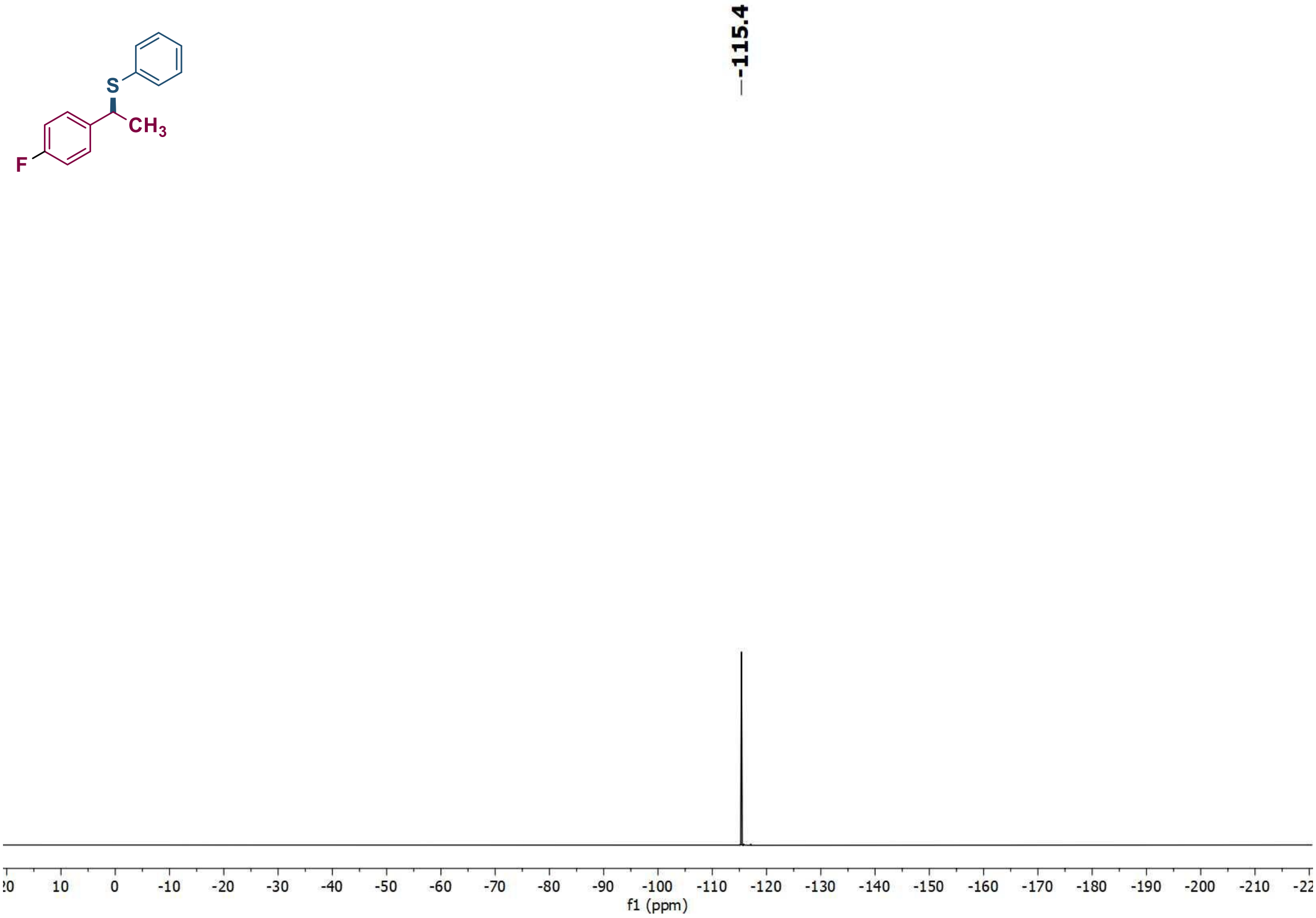

Figure S55. ^{19}F NMR (377 MHz, Chloroform-d, 25°C) of (1-(4-fluorophenyl)ethyl)(phenyl)sulfane (**4e**).

Benzhydryl(phenyl)sulfane (**4f**)

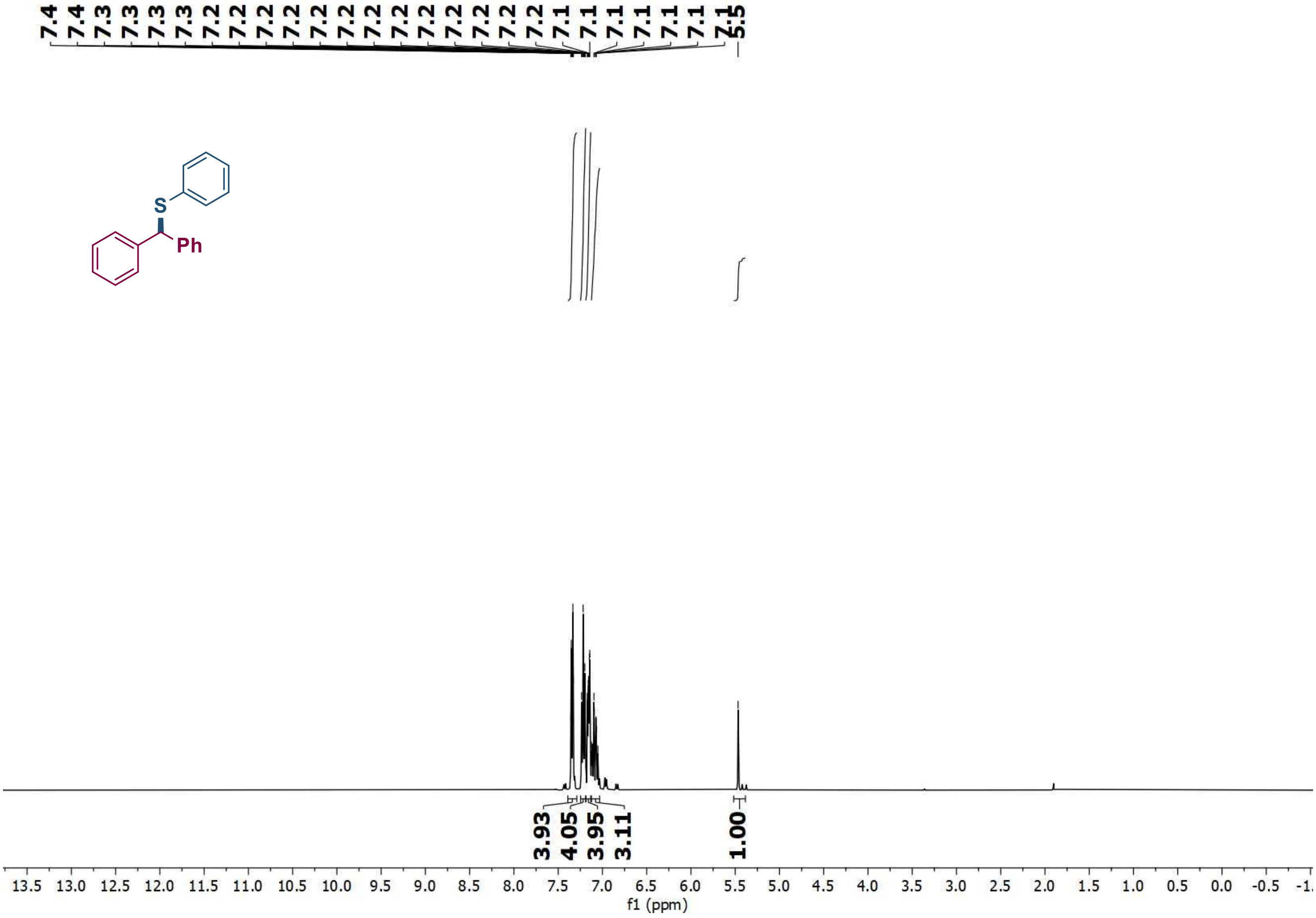

Figure S56. ^{1}H NMR (400 MHz, Chloroform-d, 25°C) of benzhydryl(phenyl)sulfane (**4f**).

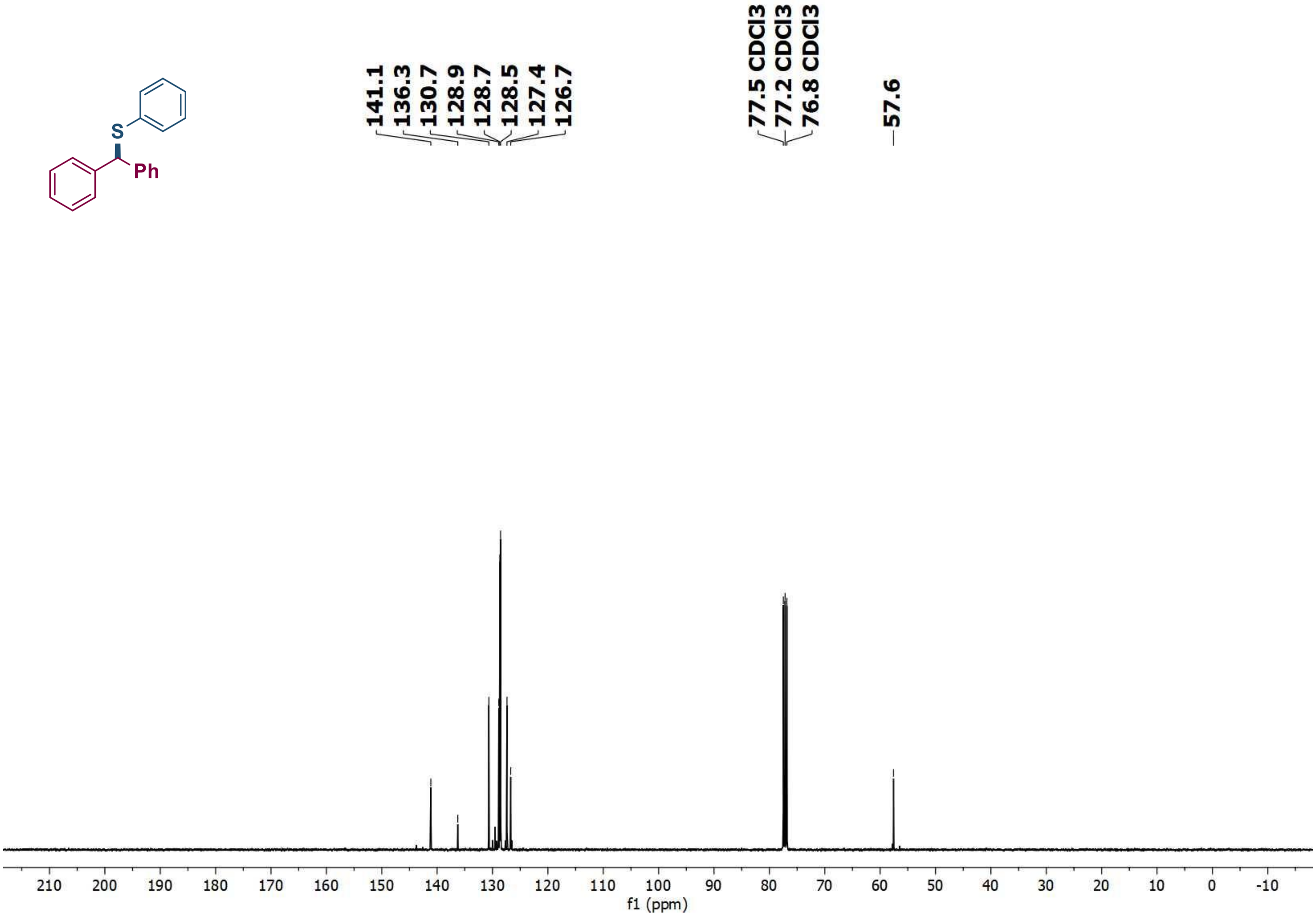

Figure S57. ^{13}C{1H} NMR (101 MHz, Chloroform-d, 25°C) of benzhydryl(phenyl)sulfane (**4f**).

Phenyl(2-(*p*-tolyl)propan-2-yl)sulfane (**4g**)

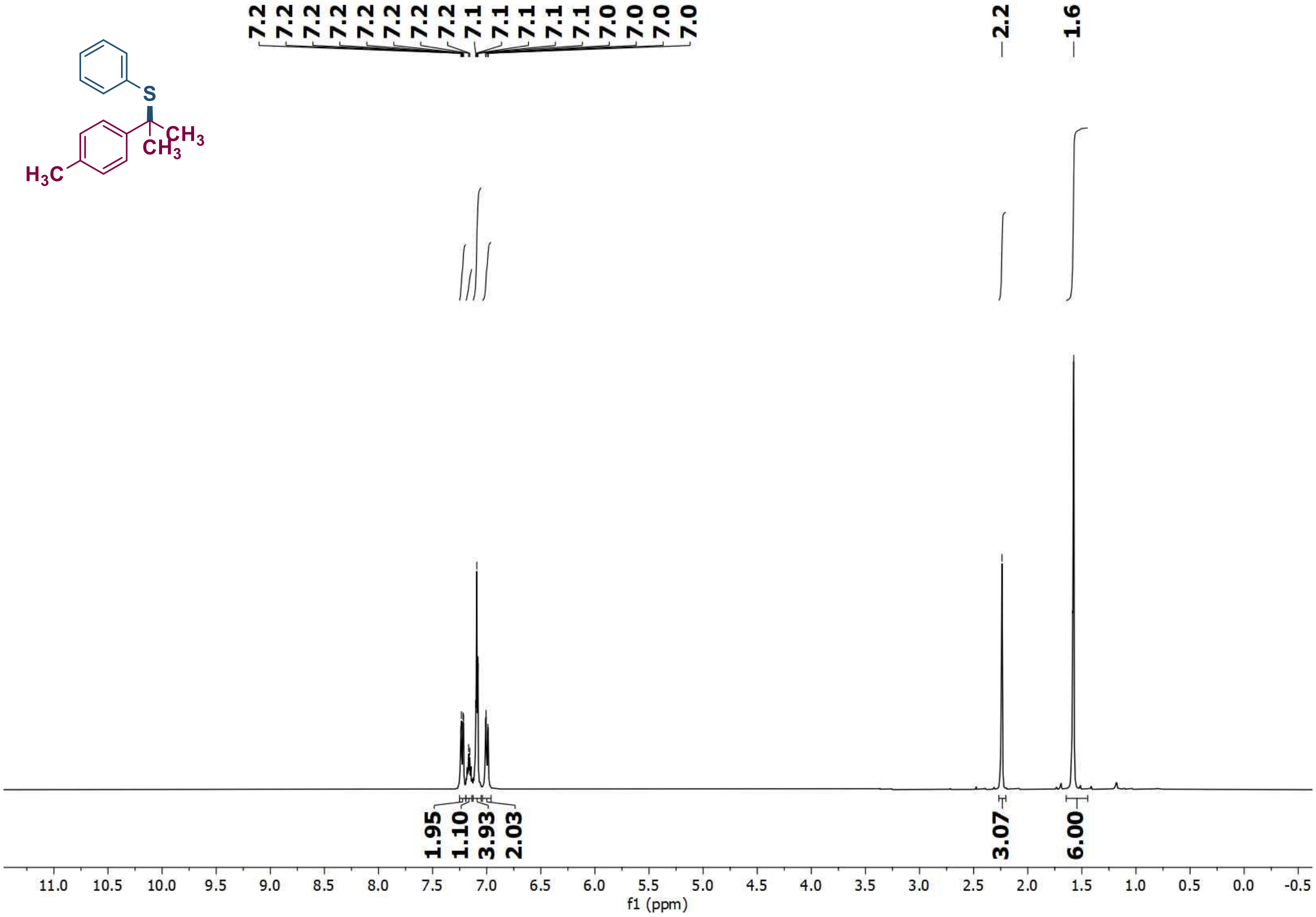

Figure S58. ^{1}H NMR (400 MHz, Chloroform-d, 25°C) of phenyl(2-(p-tolyl)propan-2-yl)sulfane (**4g**).

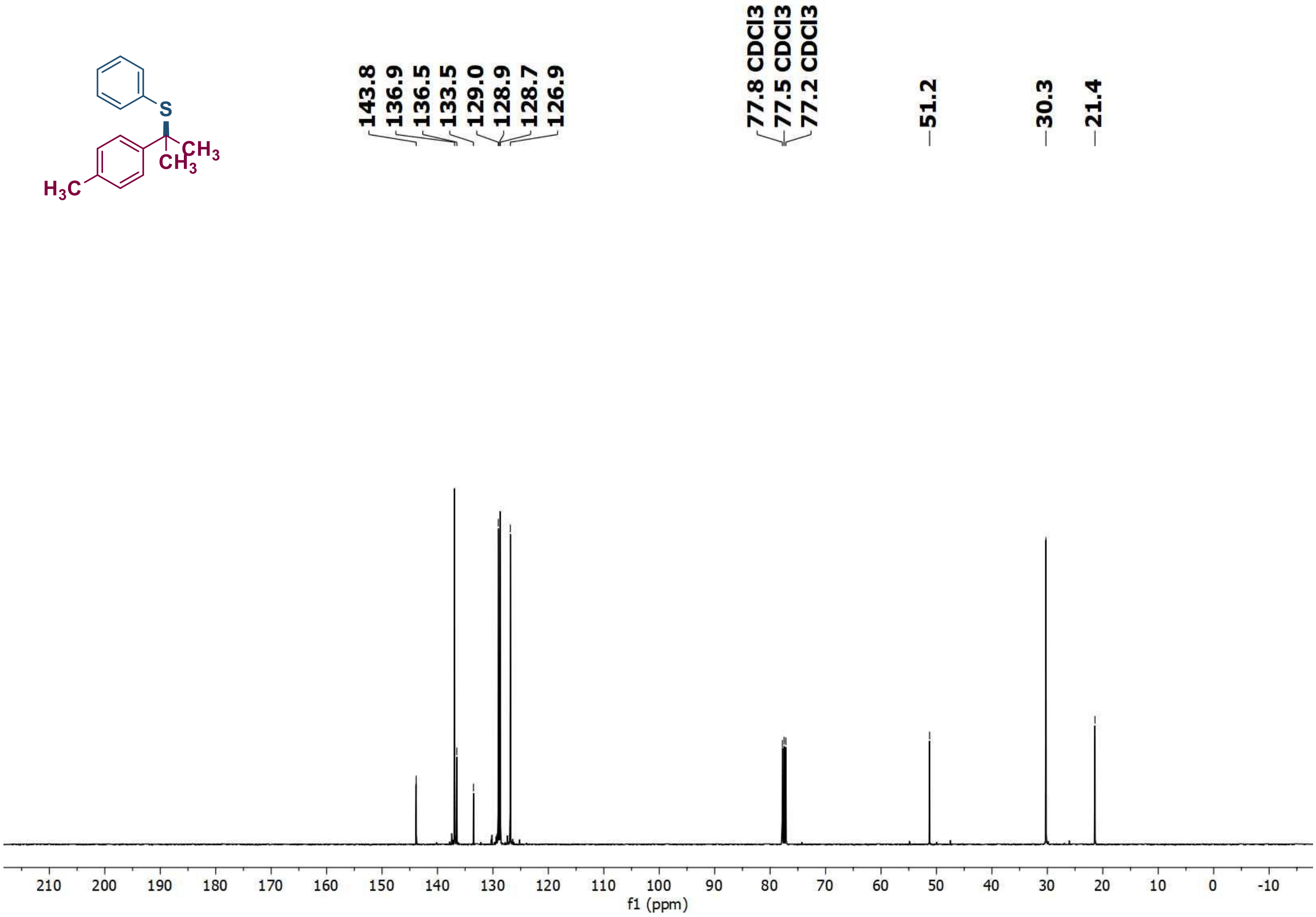

Figure S59. $^{13}C\{^{1}H\}$ NMR (101 MHz, Chloroform-d, 25°C) of phenyl(2-(p-tolyl)propan-2-yl)sulfane (**4g**).

(1-(4-Chlorophenyl)ethyl)(phenyl)sulfane (**4h**)

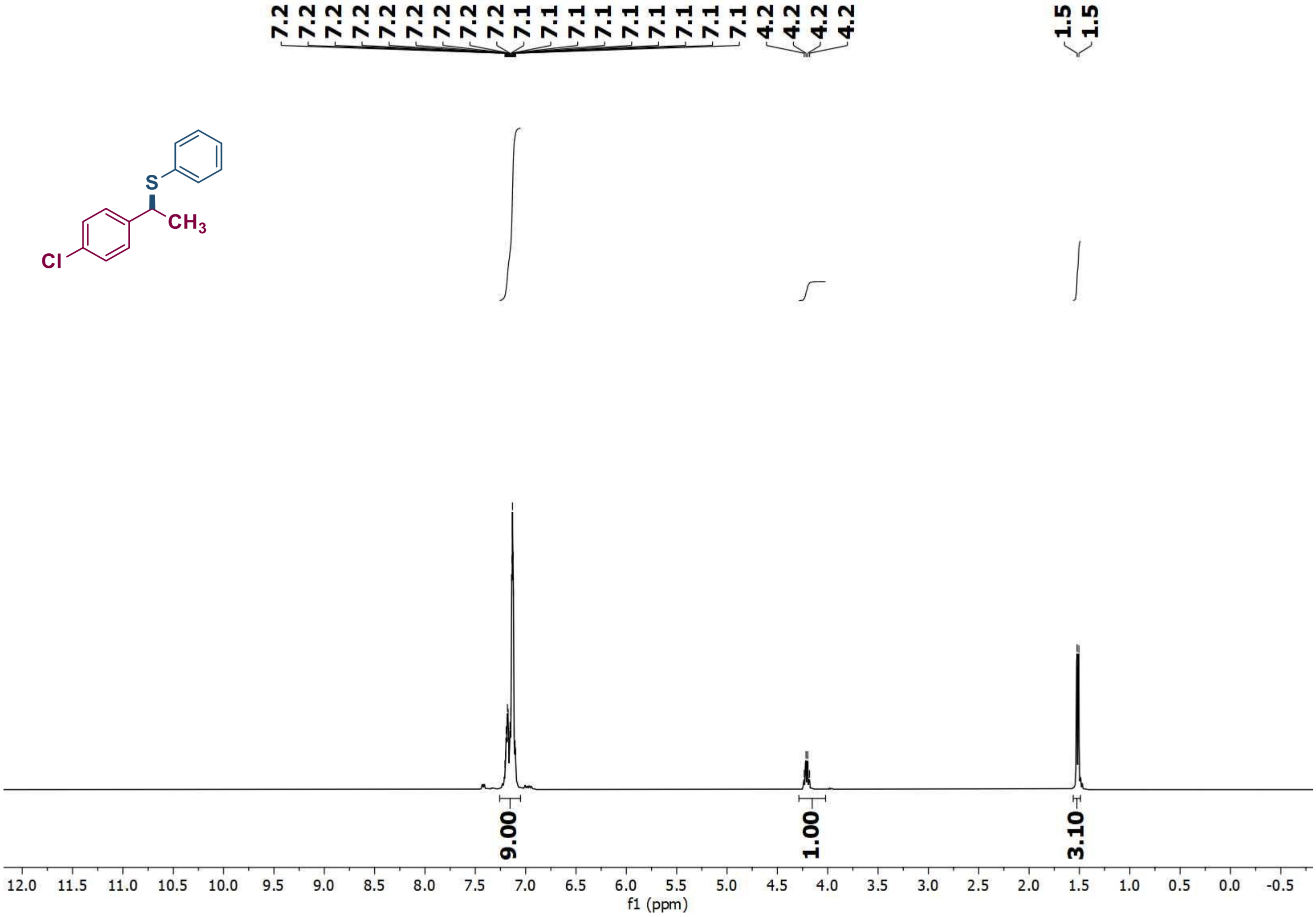

Figure S60. ^{1}H NMR (400 MHz, Chloroform-d, 25°C) of (1-(4-chlorophenyl)ethyl)(phenyl)sulfane (**4h**).

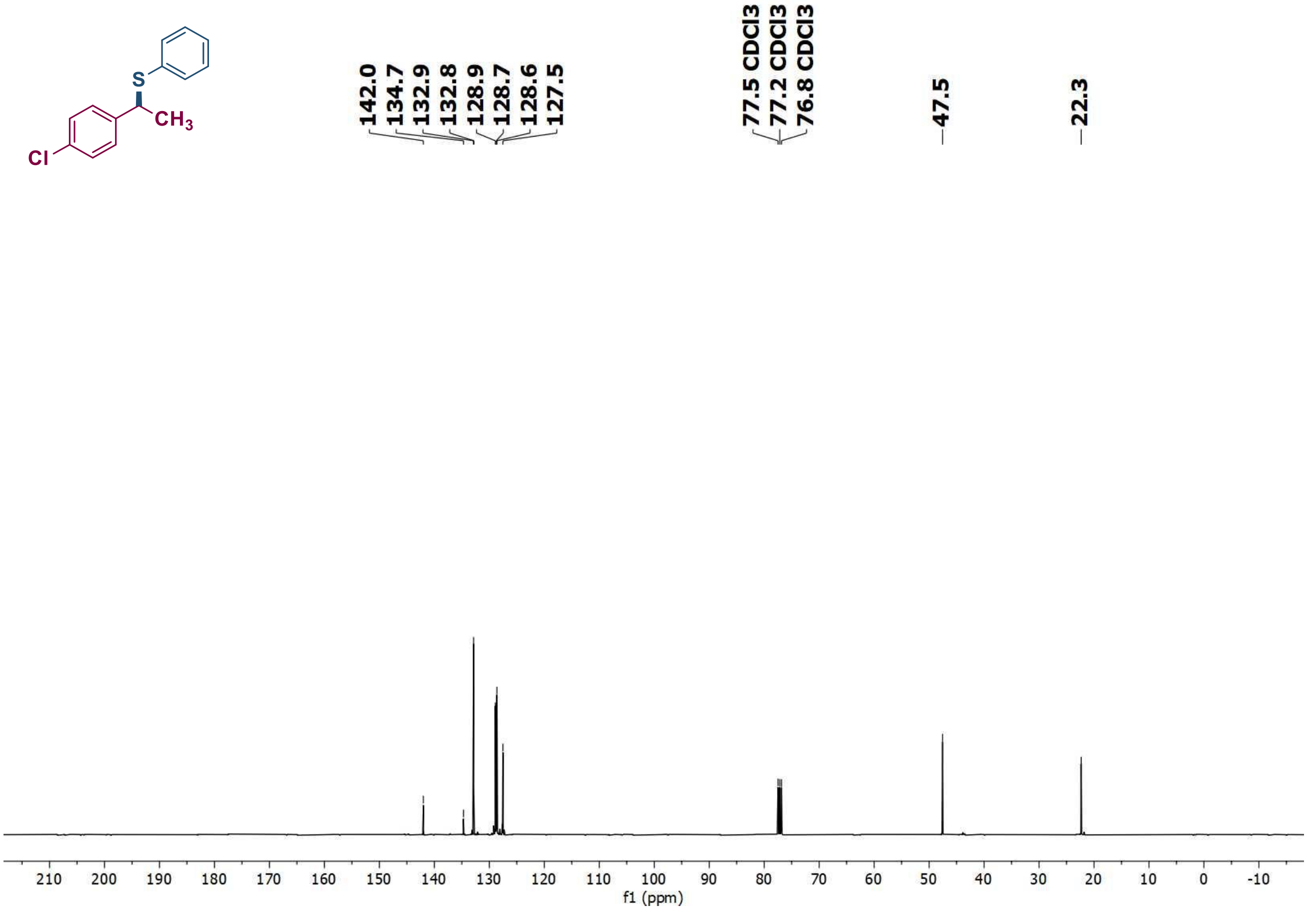

Figure S61. ^{13}C{^{1}H} NMR (101 MHz, Chloroform-d, 25°C) of (1-(4-chlorophenyl)ethyl)(phenyl)sulfane (**4h**).

1-Methoxy-4-(4-methylpent-4-en-2-yl)benzene (**6a**)

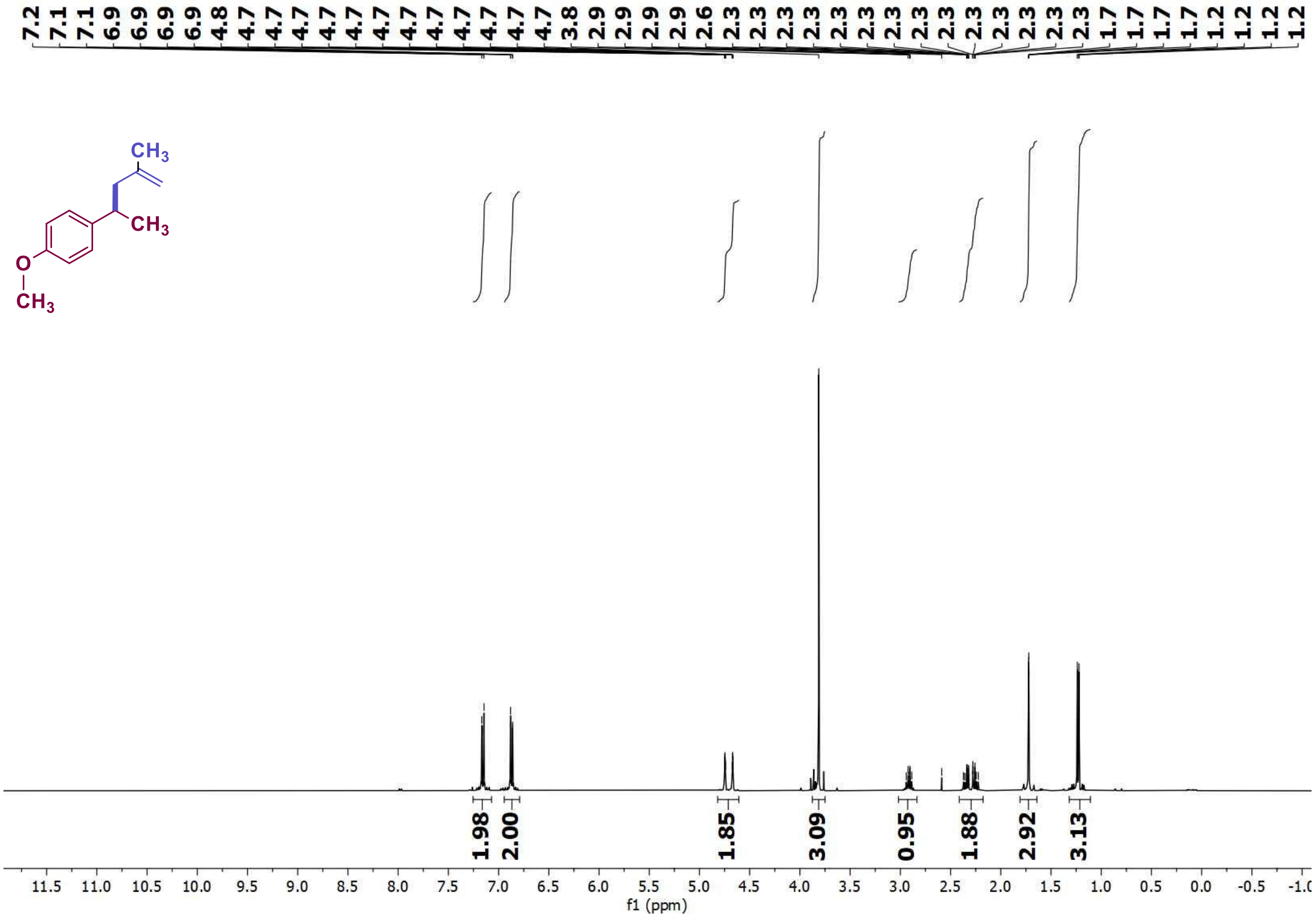

Figure S62. ^{1}H NMR (400 MHz, Chloroform-d, 25°C) of 1-methoxy-4-(4-methylpent-4-en-2-yl)benzene (**6a**).

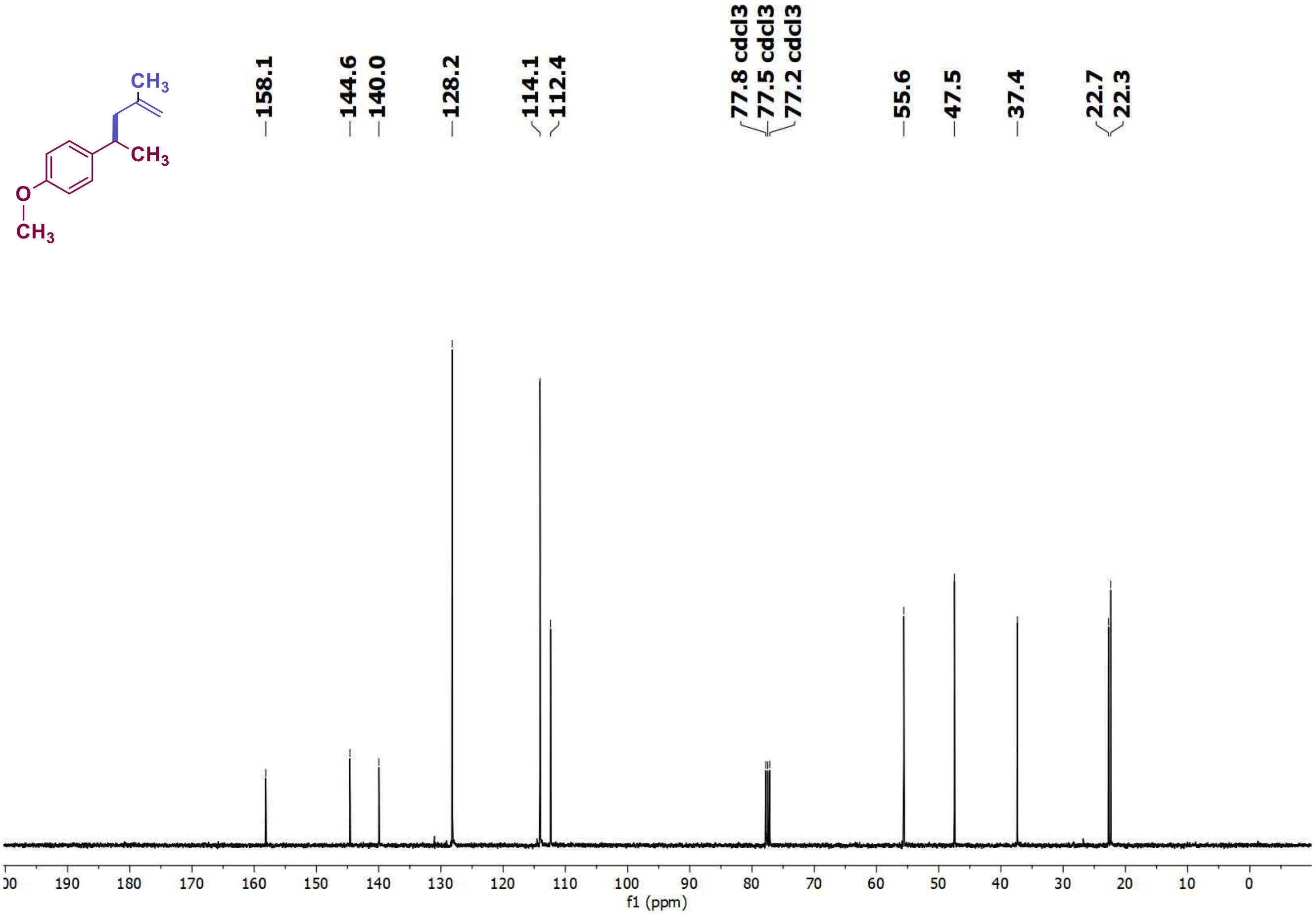

Figure S63. $^{13}C\{^1H\}$ NMR (101 MHz, Chloroform-d, 25°C) of 1-methoxy-4-(4-methylpent-4-en-2-yl)benzene (**6a**).

1-Methoxy-4-(pent-4-en-2-yl)benzene (**6b**)

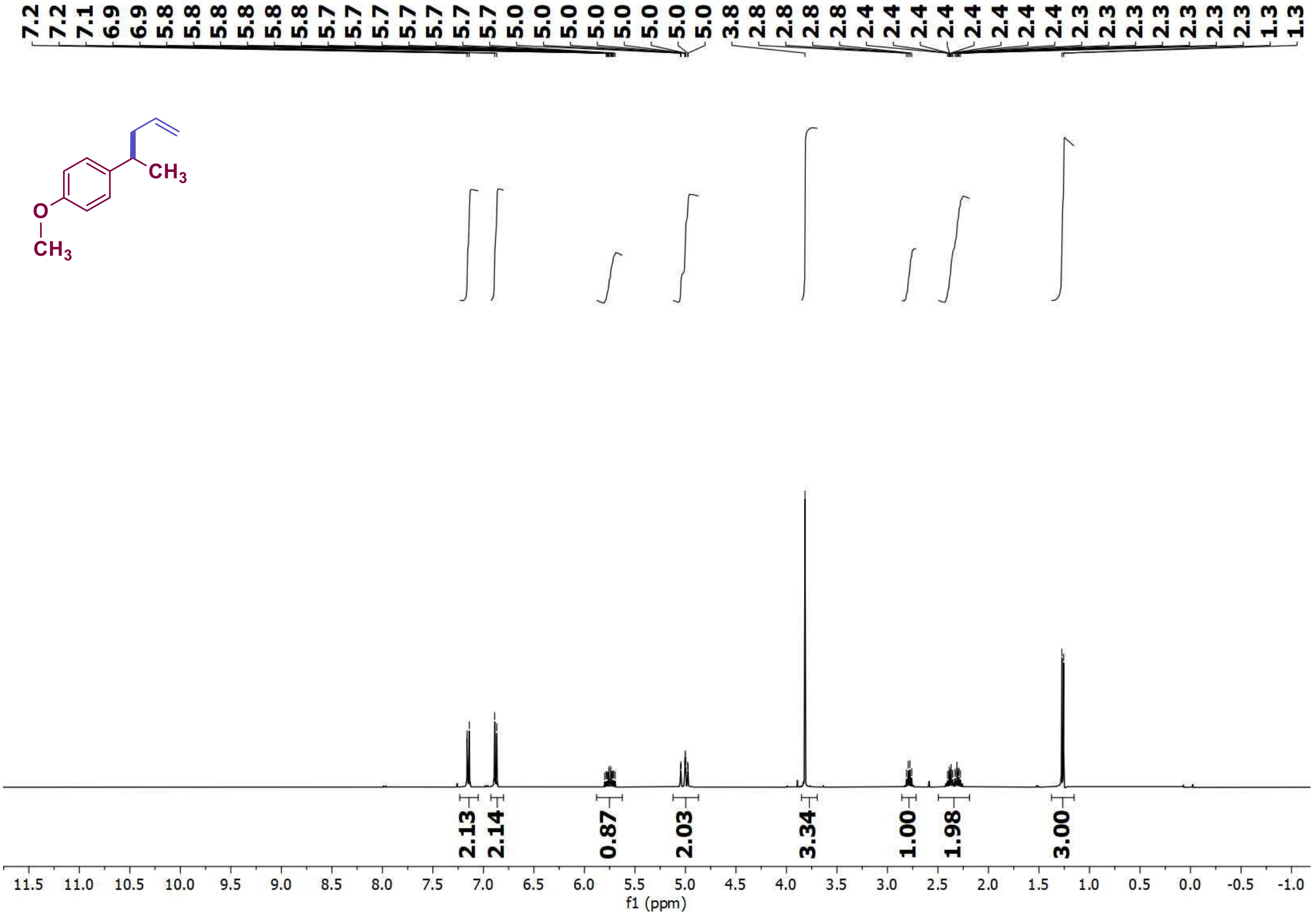

Figure S64. ^{1}H NMR (400 MHz, Chloroform-d, 25°C) of 1-methoxy-4-(pent-4-en-2-yl)benzene (**6b**).

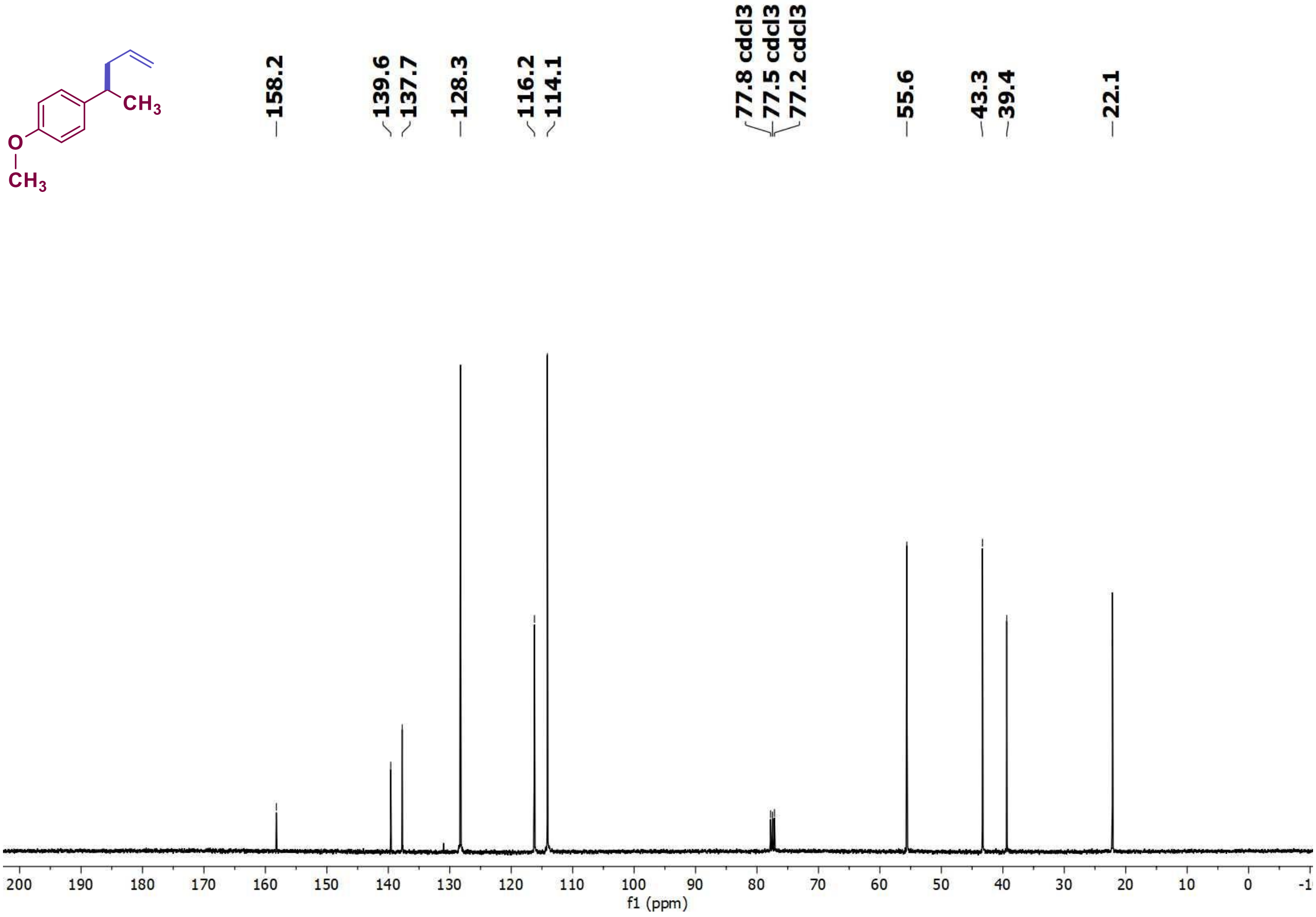

Figure S65. $^{13}C\{1H\}$ NMR (101 MHz, Chloroform-d, 25°C) of 1-methoxy-4-(pent-4-en-2-yl)benzene (**6b**).

1-Allyl-2,3-dihydro-1H-indene (**6c**)

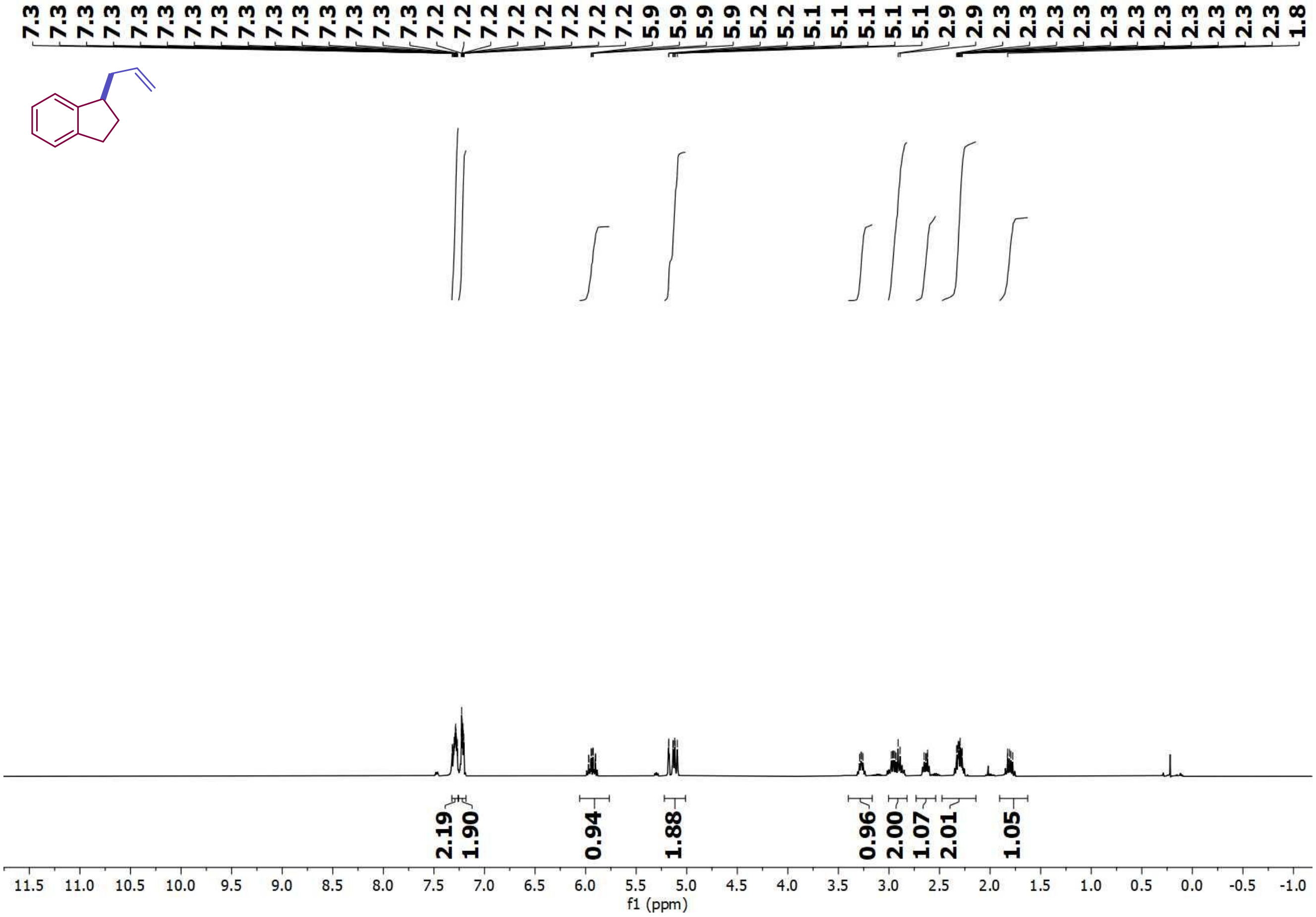

Figure S66. ^{1}H NMR (400 MHz, Chloroform-d, 25°C) of 1-allyl-2,3-dihydro-1H-indene (**6c**).

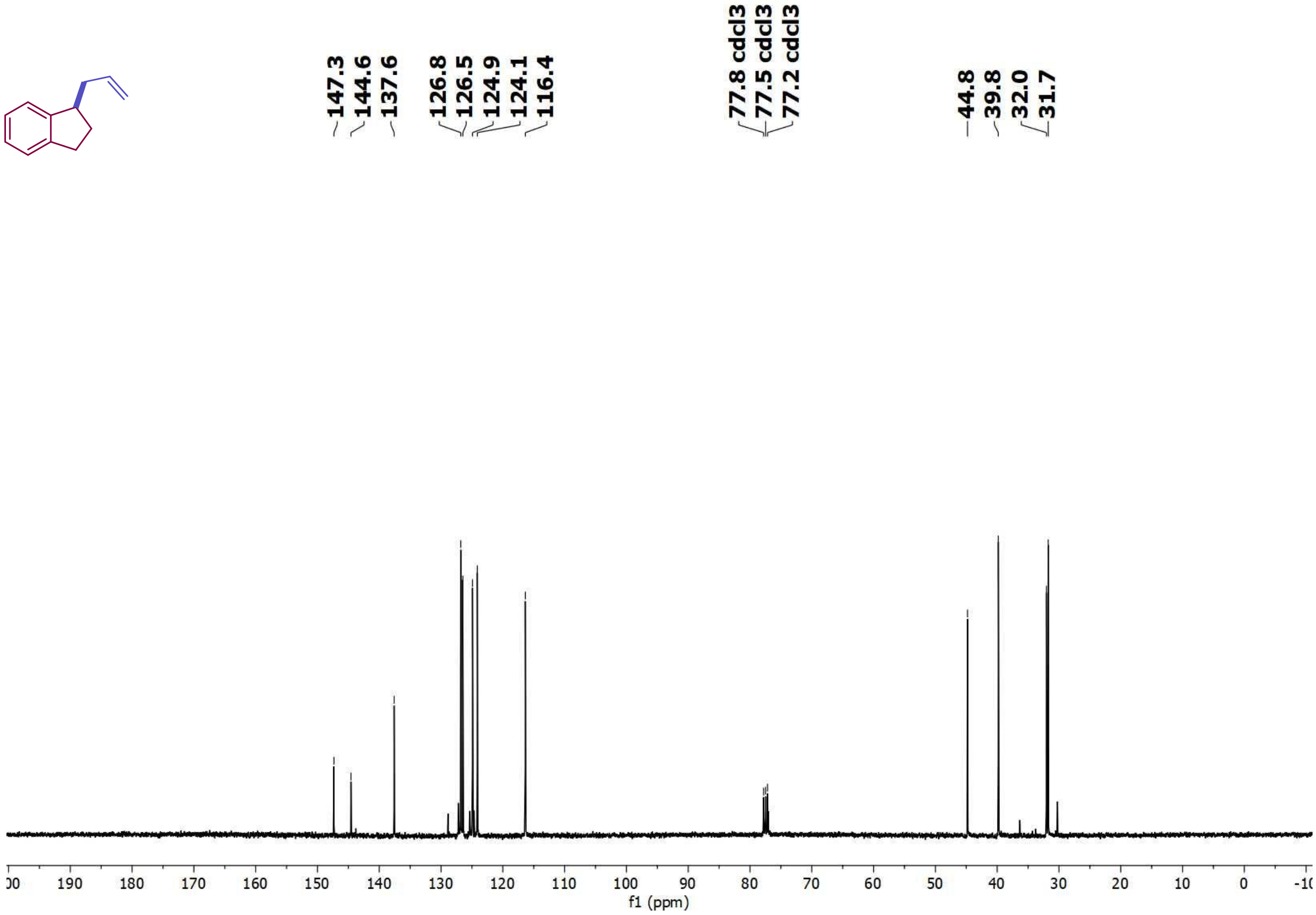

Figure S67. ^{13}C{^{1}H} NMR (101 MHz, Chloroform-d, 25°C) of 1-allyl-2,3-dihydro-1H-indene (**6c**).

1-Methoxy-4-(1-(4-methoxyphenyl)ethyl)-2,5-dimethylbenzene (**6d**)

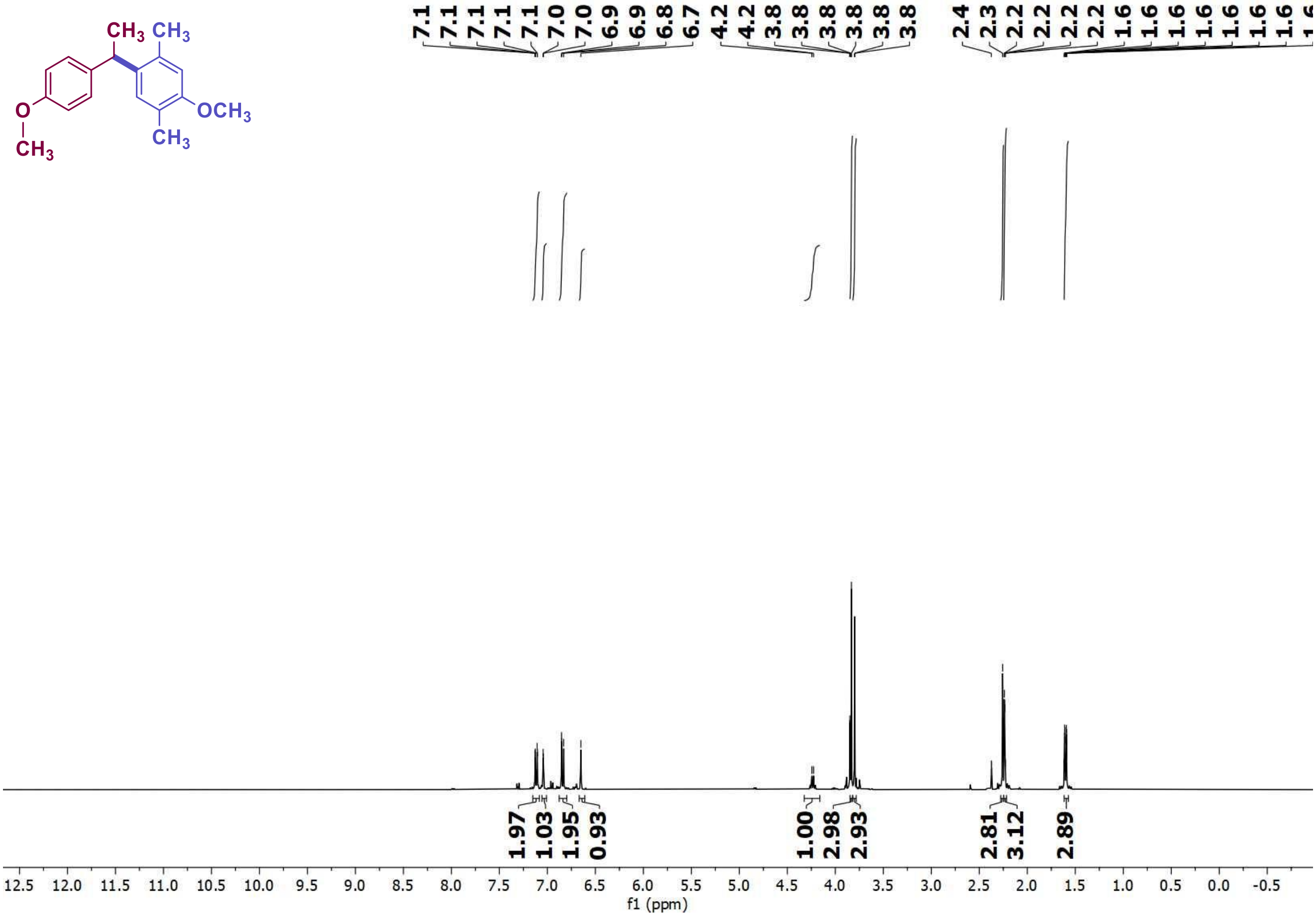

Figure S68. ^{1}H NMR (400 MHz, Chloroform-d, 25°C) of 1-methoxy-4-(1-(4-methoxyphenyl)ethyl)-2,5-dimethylbenzene (**6d**).

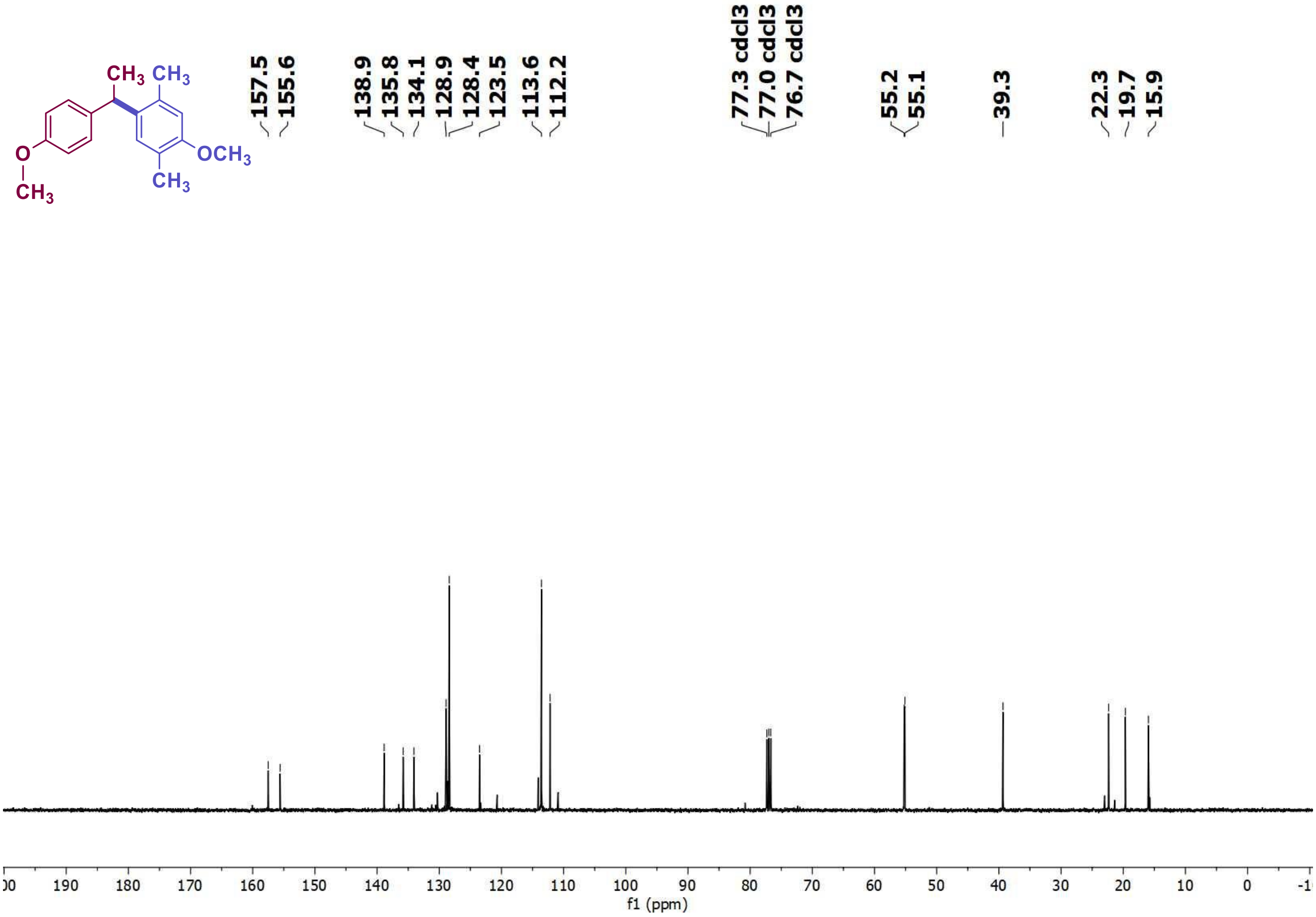

Figure S69. $^{13}C\{^{1}H\}$ NMR (101 MHz, Chloroform-d, 25°C) of 1-methoxy-4-(1-(4-methoxyphenyl)ethyl)-2,5-dimethylbenzene (**6d**).

1,3,5-Trimethoxy-2-(1-(4-methoxyphenyl)ethyl)benzene (**6e**)

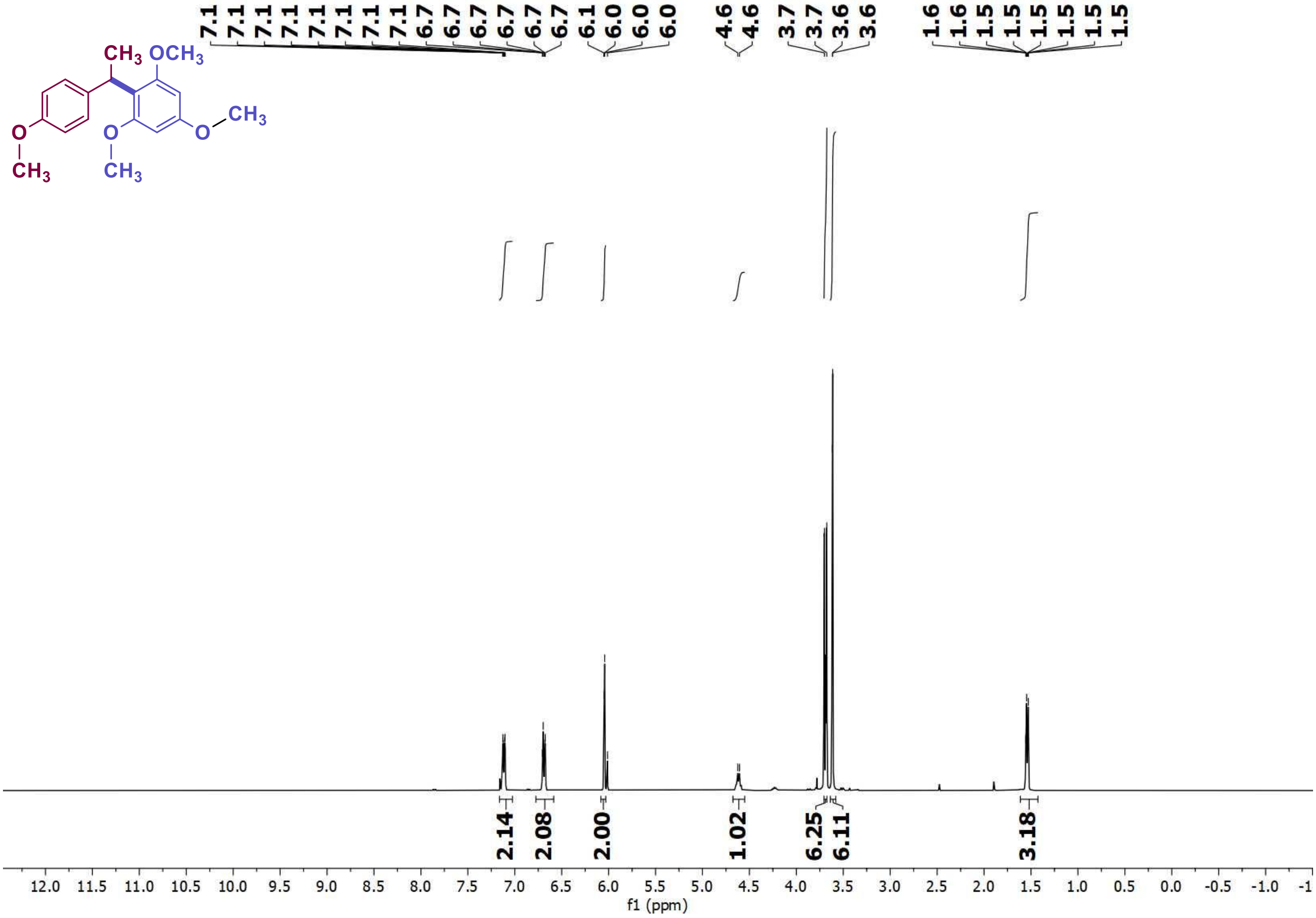

Figure S70. ^{1}H NMR (400 MHz, Chloroform-d, 25°C) of 1,3,5-trimethoxy-2-(1-(4-methoxyphenyl)ethyl)benzene (**6e**).

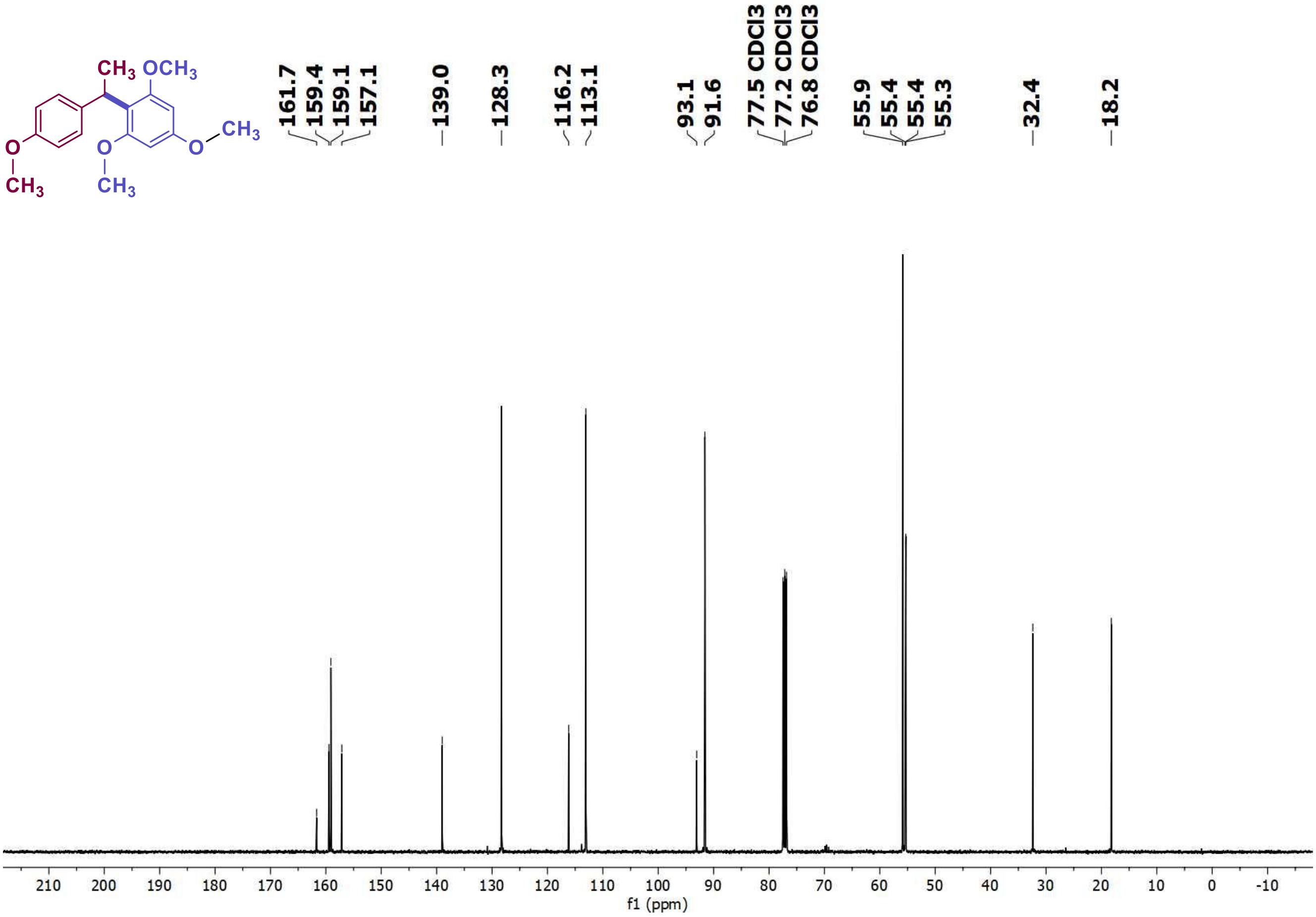

Figure S71. ^{13}C{^{1}H} NMR (101 MHz, Chloroform-d, 25°C) of 1,3,5-trimethoxy-2-(1-(4-methoxyphenyl)ethyl)benzene (**6e**).

5-Bromo-3-(1-(4-methoxyphenyl)ethyl)-*1H*-indole (**6f**)

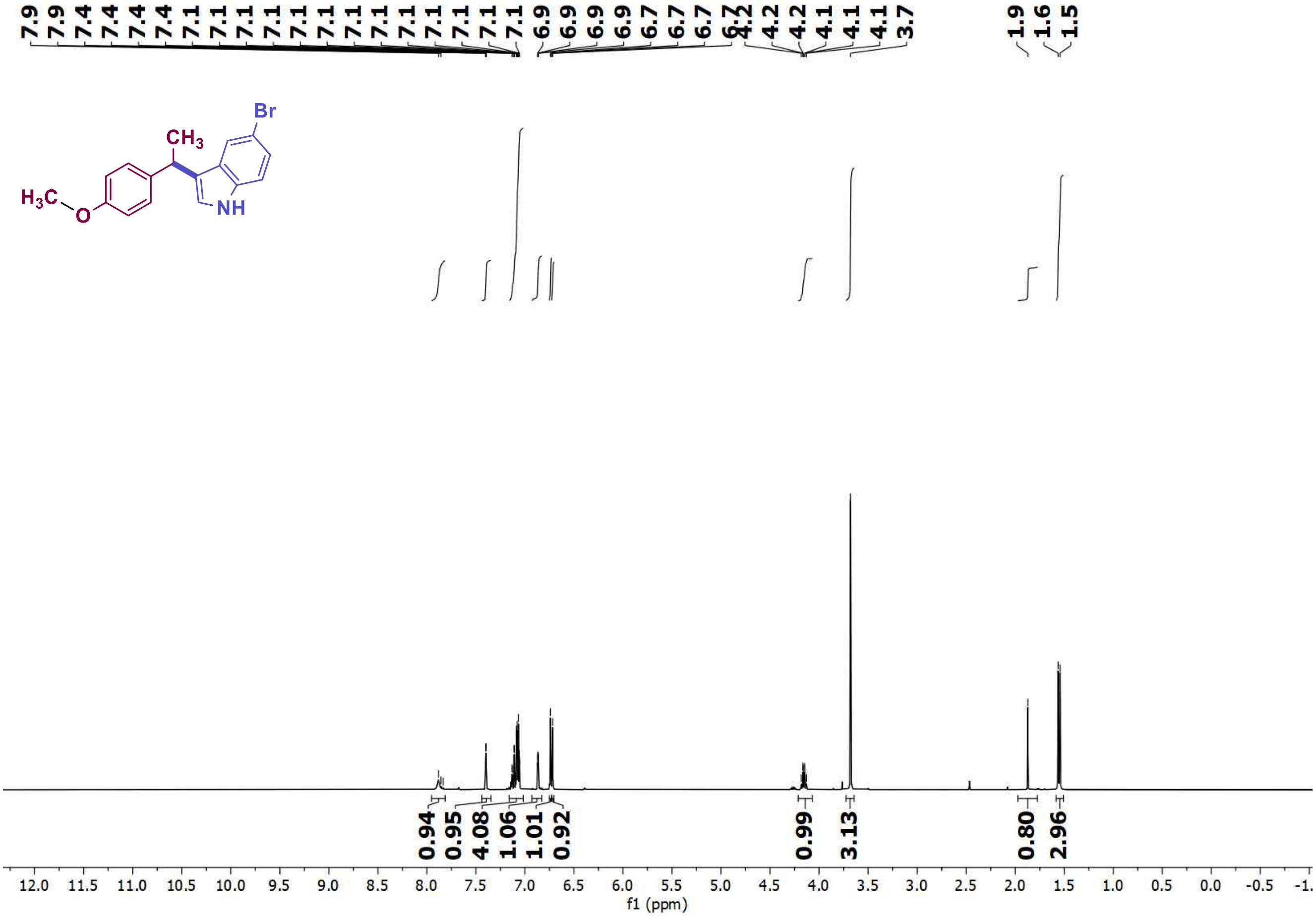

Figure S72. ^{1}H NMR (400 MHz, Chloroform-d, 25°C) of 5-bromo-3-(1-(4-methoxyphenyl)ethyl)-1H-indole (**6f**).

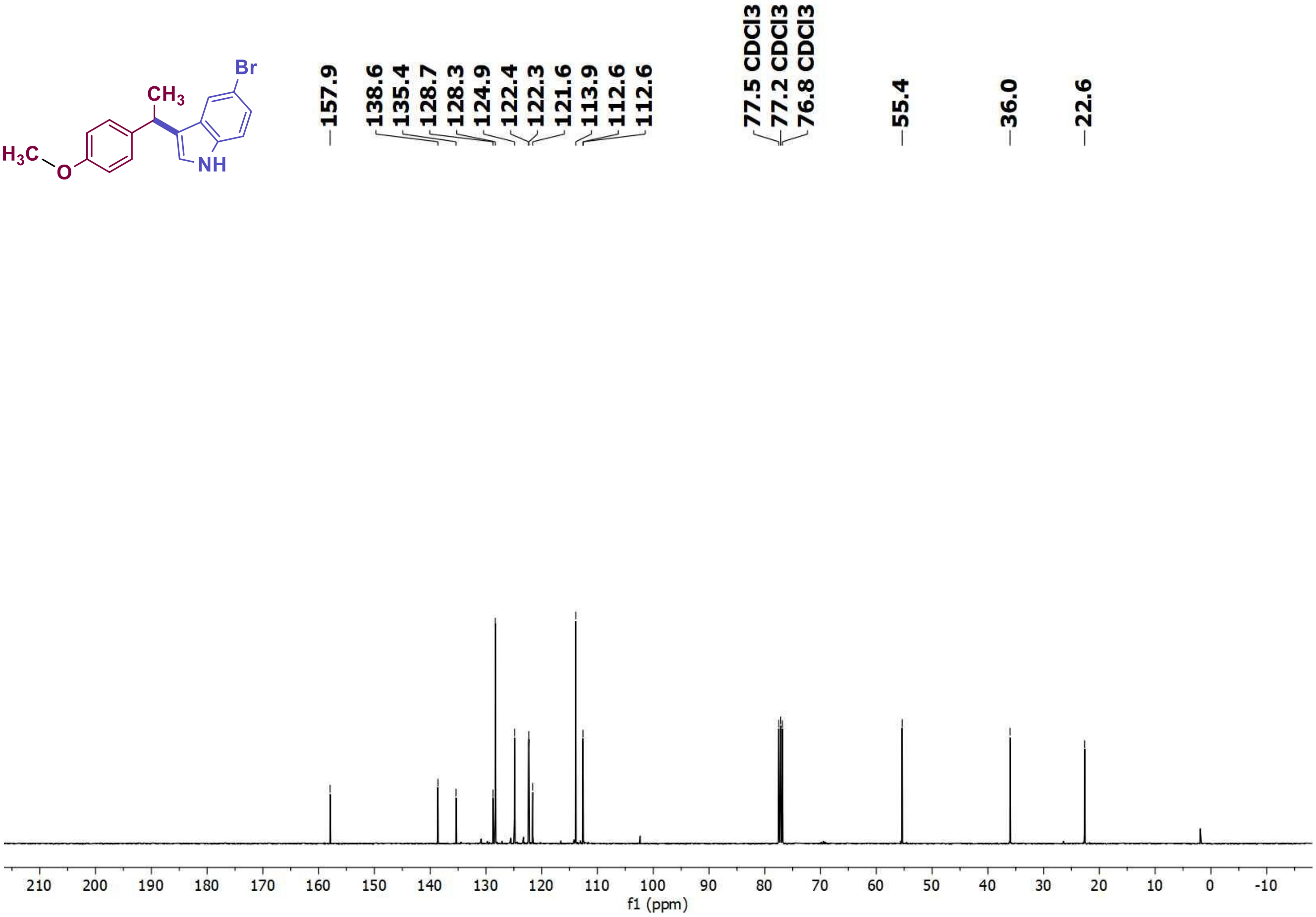

Figure S73. ^{13}C{^{1}H} NMR (101 MHz, Chloroform-d, 25°C) of 5-bromo-3-(1-(4-methoxyphenyl)ethyl)-1H-indole (**6f**).

3-(1-(4-Methoxyphenyl)ethyl)-1-methyl-*1H*-indole (**6g**)

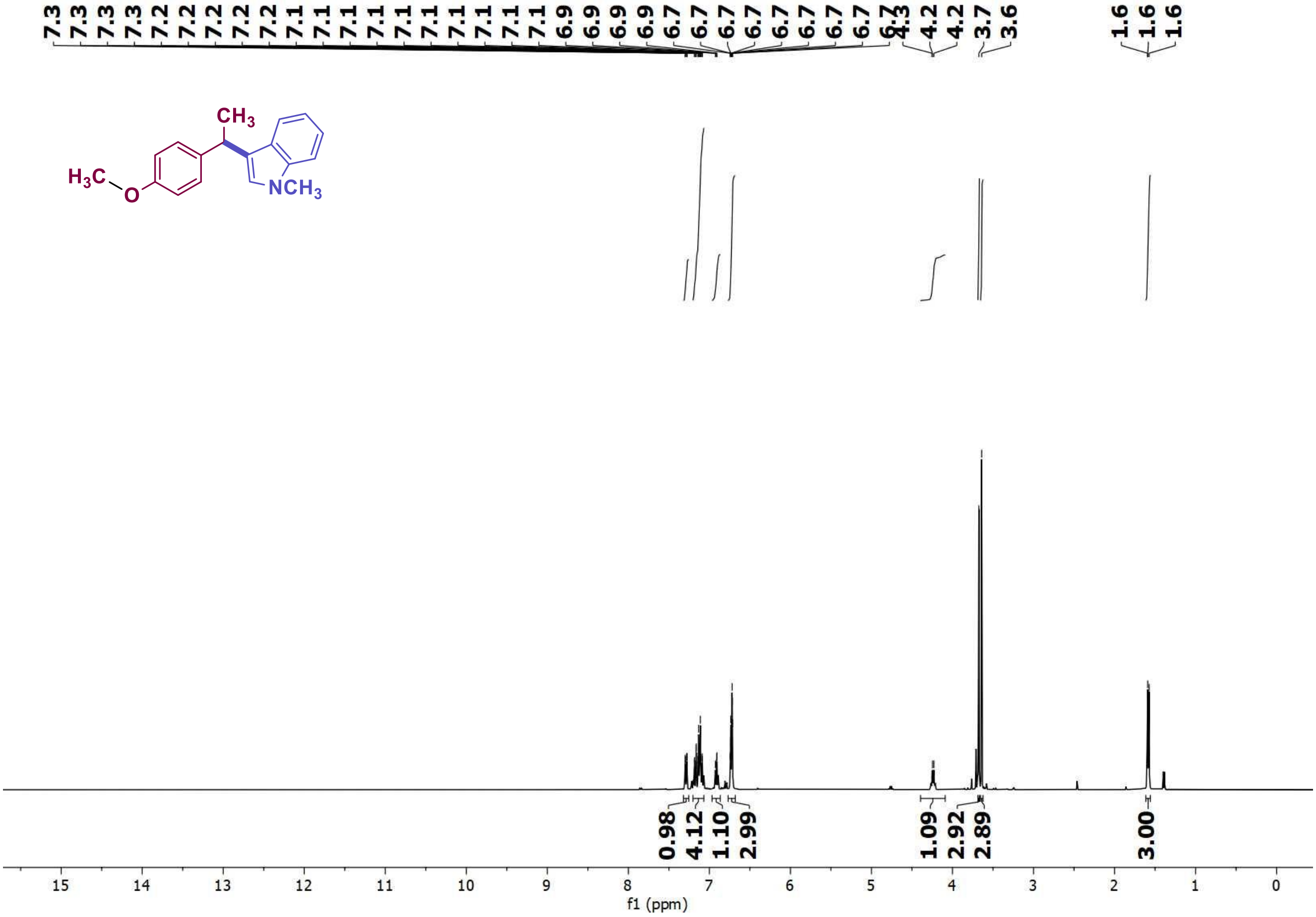

Figure S74. ^{1}H NMR (400 MHz, Chloroform-d, 25°C) of 3-(1-(4-methoxyphenyl)ethyl)-1-methyl-1H-indole (**5b**).

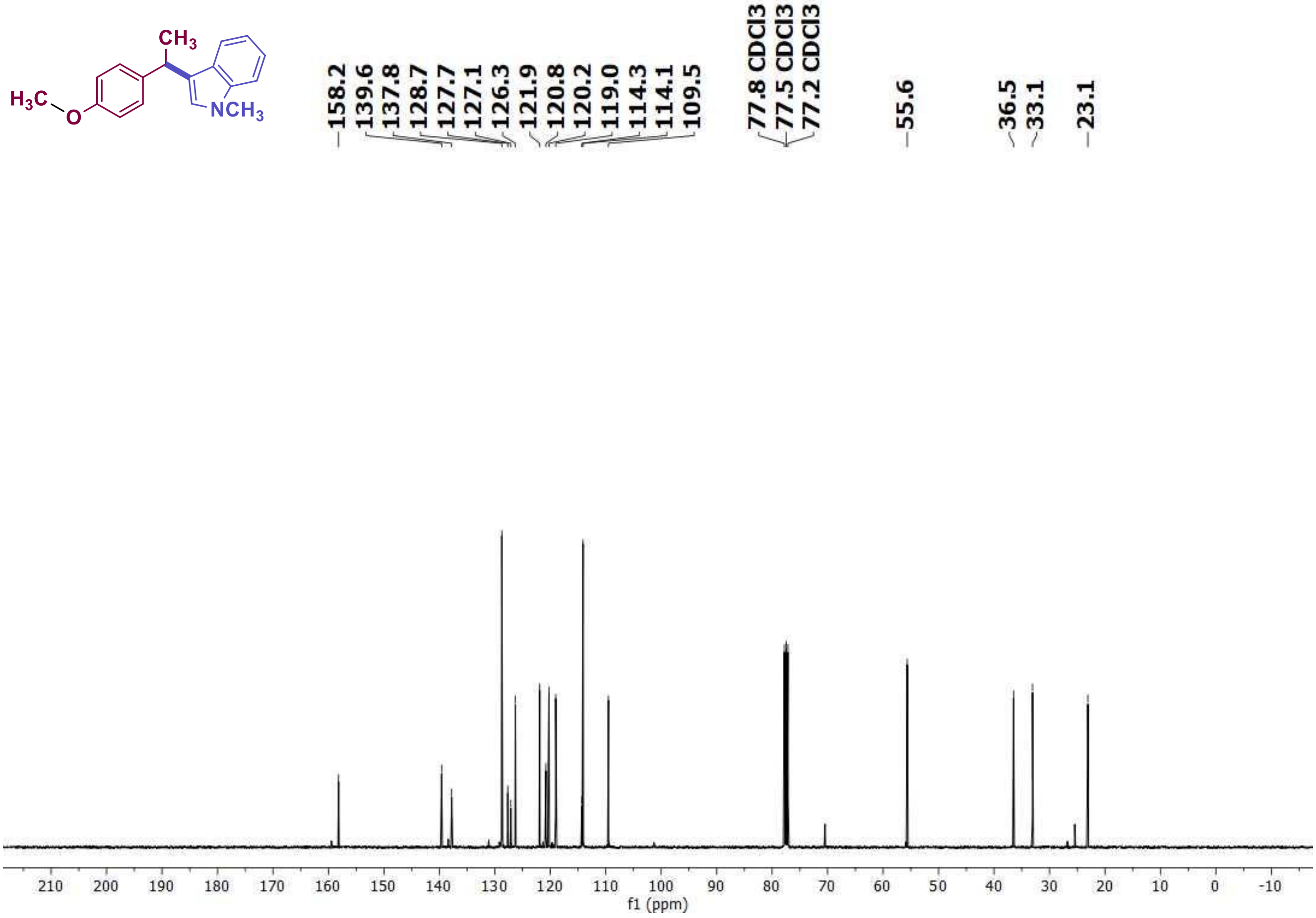

Figure S75. $^{13}C\{^{1}H\}$ NMR (101 MHz, Chloroform-d, 25°C) of 3-(1-(4-methoxyphenyl)ethyl)-1-methyl-1H-indole (**5b**).

1-Methoxy-4-(1-(prop-2-yn-1-yloxy)ethyl)benzene (**7a**)

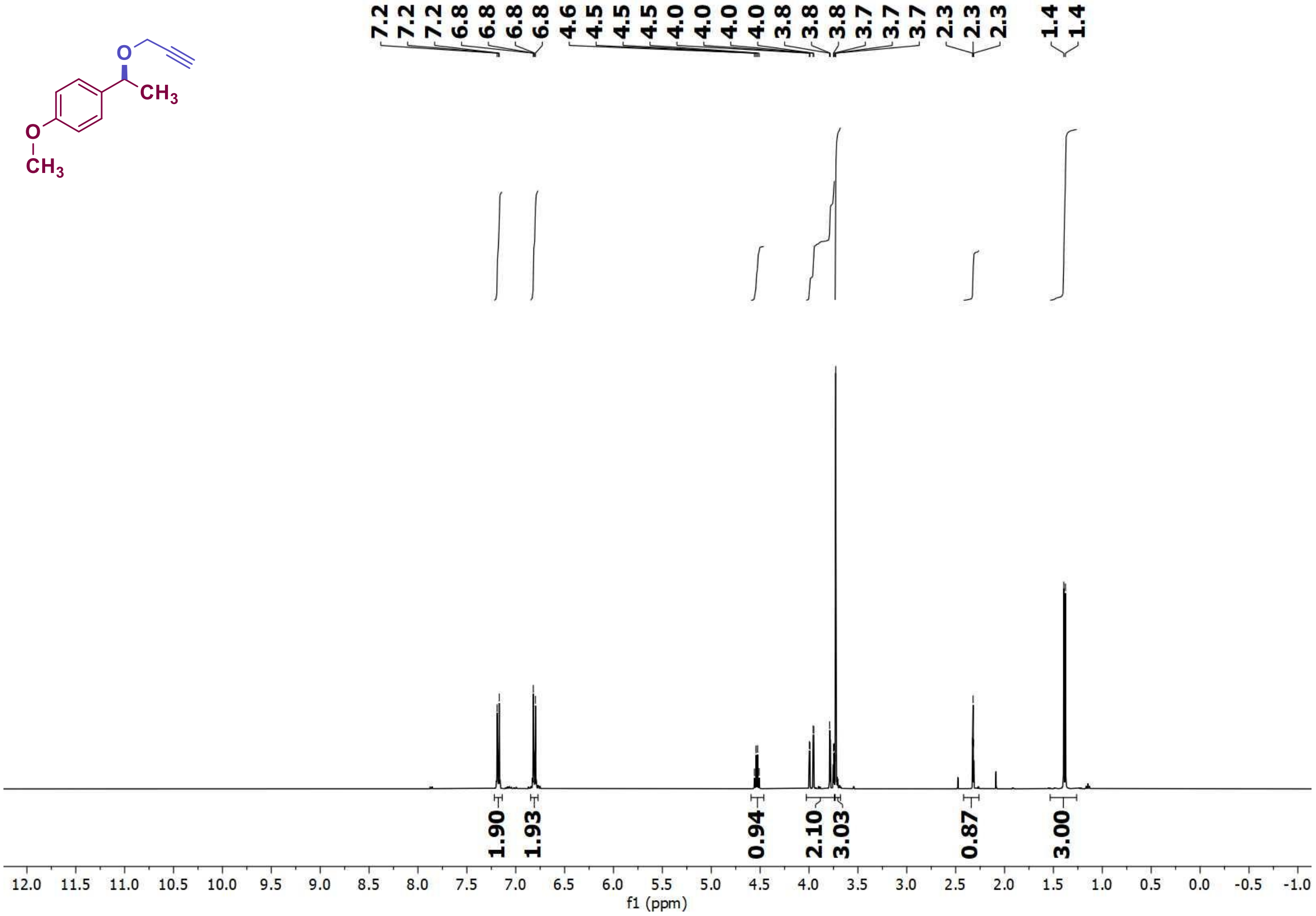

Figure S76. ^{1}H NMR (400 MHz, Chloroform-d, 25°C) of 1-methoxy-4-(1-(prop-2-yn-1-yloxy)ethyl)benzene (**7a**).

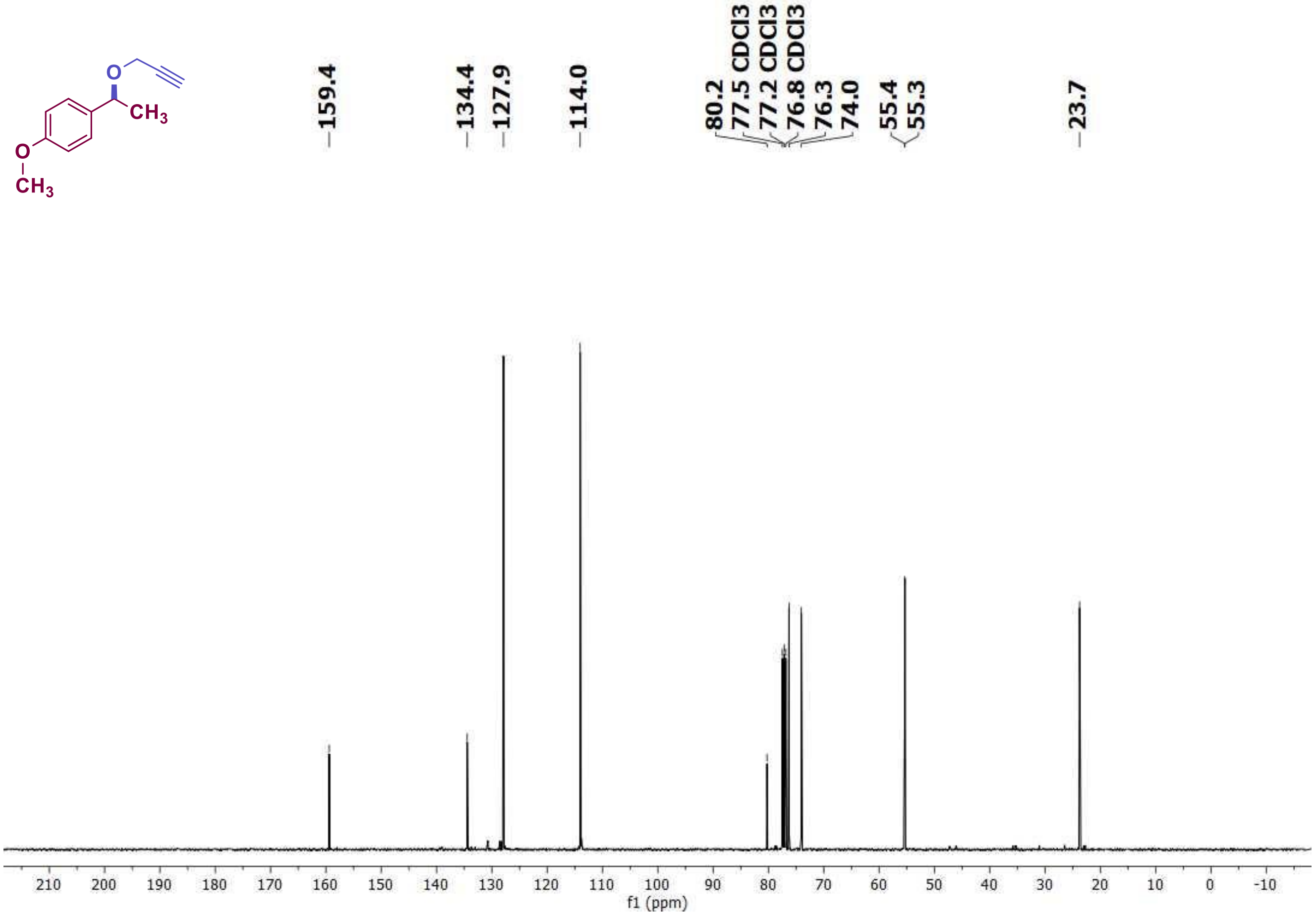

Figure S77. $^{13}C\{^{1}H\}$ NMR (101 MHz, Chloroform-d, 25°C) of 1-methoxy-4-(1-(prop-2-yn-1-yloxy)ethyl)benzene (**7a**).

REFERENCES

1 K. Kuciński and G. Hreczycho, *Eur. J. Org. Chem.*, 2017, **2017**, 5572–5581.

2 H. Yi, C. Song, Y. Li, C.-W. Pao, J.-F. Lee and A. Lei, *Chem Eur J*, 2016, **22**, 18331–18334.

3 T. Nishio, S. Yoshioka, K. Hasegawa, K. Yahata, K. Kanomata and S. Akai, *Eur. J. Org. Chem.*, 2021, **2021**, 4417–4422.

4 F. Santoro, M. Mariani, F. Zaccheria, R. Psaro and N. Ravasio, *Beilstein J. Org. Chem.*, 2016, **12**, 2627–2635.

5 Q. Ding, B. Cao, J. Yuan, X. Liu and Y. Peng, *Org. Biomol. Chem.*, 2011, **9**, 748–751.

6 J. P. Richard and W. P. Jencks, *J. Am. Chem. Soc.*, 1984, **106**, 1373–1383.

7 M. Markwitz, K. Labrzycki, L. Azcune, A. Landa and K. Kuciński, *Sci. Rep.*, 2023, **13**, 20624.

8 R. A. Croft, J. J. Mousseau, C. Choi and J. A. Bull, *Chem. Eur. J.*, 2018, **24**, 818–821.

9 H. Lehmkuhl and R. McLane, *Liebigs Ann. Chem.*, 1980, **1980**, 736–743.

10 T. Saito, Y. Nishimoto, M. Yasuda and A. Baba, *J. Org. Chem.*, 2006, **71**, 8516–8522.

11 M. A. Radtke and T. H. Lambert, *Chem. Sci.*, 2018, **9**, 6406–6410.

12 S. K. Talluri and A. Sudalai, *Org. Lett.*, 2005, **7**, 855–857.
